# A requirement for *STAG2* in replication fork progression creates a targetable synthetic lethality in cohesin-mutant cancers

**DOI:** 10.1038/s41467-019-09659-z

**Published:** 2019-04-11

**Authors:** Gourish Mondal, Meredith Stevers, Benjamin Goode, Alan Ashworth, David A. Solomon

**Affiliations:** 1 Department of Pathology, University of California, San Francisco, CA 94143 USA; 2 UCSF Helen Diller Family Comprehensive Cancer Center, San Francisco, CA 94158 USA; 3 Division of Hematology and Oncology, Department of Medicine, University of California, San Francisco, CA 94158 USA

## Abstract

Cohesin is a multiprotein ring that is responsible for cohesion of sister chromatids and formation of DNA loops to regulate gene expression. Genomic analyses have identified that the cohesin subunit *STAG2* is frequently inactivated by mutations in cancer. However, the reason *STAG2* mutations are selected during tumorigenesis and strategies for therapeutically targeting mutant cancer cells are largely unknown. Here we show that *STAG2* is essential for DNA replication fork progression, whereby *STAG2* inactivation in non-transformed cells leads to replication fork stalling and collapse with disruption of interaction between the cohesin ring and the replication machinery as well as failure to establish SMC3 acetylation. As a consequence, *STAG2* mutation confers synthetic lethality with DNA double-strand break repair genes and increased sensitivity to select cytotoxic chemotherapeutic agents and PARP or ATR inhibitors. These studies identify a critical role for STAG2 in replication fork procession and elucidate a potential therapeutic strategy for cohesin-mutant cancers.

## Abstract (web summary)

Cohesin complex mediates cohesion of sister chromatids and DNA loop formation. Here the authors show another role of cohesin in replication fork progression, suggesting a potential therapeutic strategy for cohesin-mutant cancers.

## Introduction

Cohesin is a multi-protein complex composed of four core subunits (SMC1A, SMC3, RAD21, and either STAG1 or STAG2) that is responsible for the cohesion of sister chromatids. Cohesin genes were originally identified in yeast as mutants that displayed premature separation of sister chromatids, and were later identified as being highly conserved from yeast to mammals^1^. The cohesin subunits form a ring-shaped structure that encircles chromatin, which is loaded onto chromatin in early G1 phase of the cell cycle immediately following cytokinesis and concatenates sister chromatids during DNA replication in S phase. Cohesin remains chromatin bound specifically at centromeres in prophase of mitosis while the majority of cohesin along chromatid arms is released, and then the remainder of chromatin-bound cohesin is cleaved at the metaphase to anaphase transition to enable segregation of the sister chromatids into two daughter cells. Recent studies have found that cohesin containing the more abundant STAG2 subunit is essential for chromatid cohesion at centromeres and along chromosome arms, while cohesin containing the less abundant STAG1 subunit is essential for chromatid cohesion specifically at telomeres^2,3^.

In addition to its canonical role in sister chromatid cohesion, studies have indicated that cohesin is essential for a multitude of other cellular functions. Notably, cohesin was recently shown to be required for the formation of chromatin loops, such as those that bring together distant superenhancers with immediate upstream promoter sequences to regulate gene expression^4–6^. While cohesin forms a ring-like structure that encircles chromatin, no DNA binding motifs with nucleotide sequence specificity have been identified within the core cohesin subunits. However, emerging studies have shown that cohesin is enriched at specific chromatin loci including active transcriptional sites and pericentric heterochromatin, suggesting cohesin localization is directed by specific DNA-binding regulatory proteins. The CCCTC-binding factor (CTCF) has been identified as a direct binding partner of STAG2 that is dispensable for cohesin loading onto chromatin but is required for cohesin enrichment at specific enhancer regulatory loci throughout the genome^7,8^.

While cohesin is known to be loaded onto chromatin immediately following cytokinesis at the completion of mitosis, it is during DNA replication in S-phase when this pool of cohesin concatenates sister chromatids to establish cohesion^9–11^. Recent studies have demonstrated that the MCM replicative helicase complex is critical for this cohesion establishment during S-phase^12,13^. However, the extent to which cohesin is essential for DNA replication is largely unknown, as is the effect that cohesin gene mutations in human cancers might have on stability and procession of replication forks. Notably, recent studies in yeast have hypothesized a role for cohesin in replication fork dynamics^14–16^.

Germline mutations in the cohesin subunits or in genes responsible for cohesin loading (e.g., *NIPBL*) or regulation (e.g., *HDAC8* and *ESCO2*) cause a spectrum of severe developmental syndromes characterized by facial dysmorphism, growth retardation, mental retardation, and limb anomalies. Depending on the gene affected, these have been termed Cornelia de Lange syndrome, Roberts syndrome, and other eponyms, which are now all considered to be “cohesinopathies”^17–20^. Analysis of cells from affected patients found precocious separation of sister chromatids before anaphase in mitosis, which was hypothesized to be a causative mechanism in these syndromes^21^. However, subsequent studies have not observed similar defects in sister chromatid cohesion, calling into question the functional consequence of cohesin mutations in the germline of affected patients. Genome-wide transcriptional profiling in cells derived from Cornelia de Lange syndrome patients with *NIPBL* or *SMC1A* mutations versus normal subjects has revealed a conserved pattern of transcriptional dysregulation^22,23^. As a result, these cohesinopathy syndromes are now widely regarded to result from deregulated gene expression during development.

Recent genomic analyses of human cancer have identified that the cohesin genes, and *STAG2* in particular, are frequent targets of mutational inactivation in a select subset of tumor types that include glioblastoma, urothelial carcinoma, Ewing sarcoma, and myeloid leukemia^24–29^. *STAG2* has been identified as one of only 12 genes that are significantly mutated in four or more human cancer types by The Cancer Genome Atlas^30^, in which *STAG2* mutation defines molecular subgroups of these tumor types with distinct clinical outcomes^24,25,27,28^. Initial studies in glioblastoma cell lines suggested a role for *STAG2* mutations as a cause of chromosomal instability and aneuploidy during tumorigenesis^26^. However, the majority of urothelial carcinomas, Ewing sarcomas, and myeloid leukemias harboring *STAG2* mutations are actually diploid or near-diploid tumors, suggesting that cohesin mutations in cancer likely promote tumorigenesis by mechanisms unrelated to chromosome segregation^25,27–29^. The exact reasons why inactivating cohesin mutations are selected for during cancer development and progression are still uncertain. In one recent study, *STAG2* mutations were found to be acquired after therapy with RAF inhibitors in *BRAF*-mutant melanomas as a mechanism of therapeutic resistance^31^. However, the majority of *STAG2* mutations in glioblastoma, urothelial carcinoma, and Ewing sarcoma are clonal events that likely arise early during tumor development. The therapeutic consequences of cohesin mutations in these cancers are largely unknown at present, as are methodologies for treating cohesin-mutant cancers using a precision medicine approach.

Here we investigated the function of the cohesin subunit *STAG2* in primary non-transformed human cells to discern the basic cellular processes regulated by this gene that serves as a scaffold between the cohesin ring and the nuclear proteome. We find an essential role for STAG2 in the procession of DNA replication forks. Deficiency of STAG2 results in disruption of the interaction of cohesin with the replication machinery, leading to stalling and collapse of replication forks, as well as failure to establish SMC3 acetylation. As a consequence, *STAG2* deficiency confers synthetic lethality with specific DNA repair genes and increased sensitivity to select chemotherapeutic agents. Our data provide the preclinical rationale for targeted therapy-based clinical trials for cohesin mutant cancers.

## Results

### Inactivation of *STAG2* in primary human cells results in intra-S-phase cell cycle arrest and senescence

**Fig. 1:**
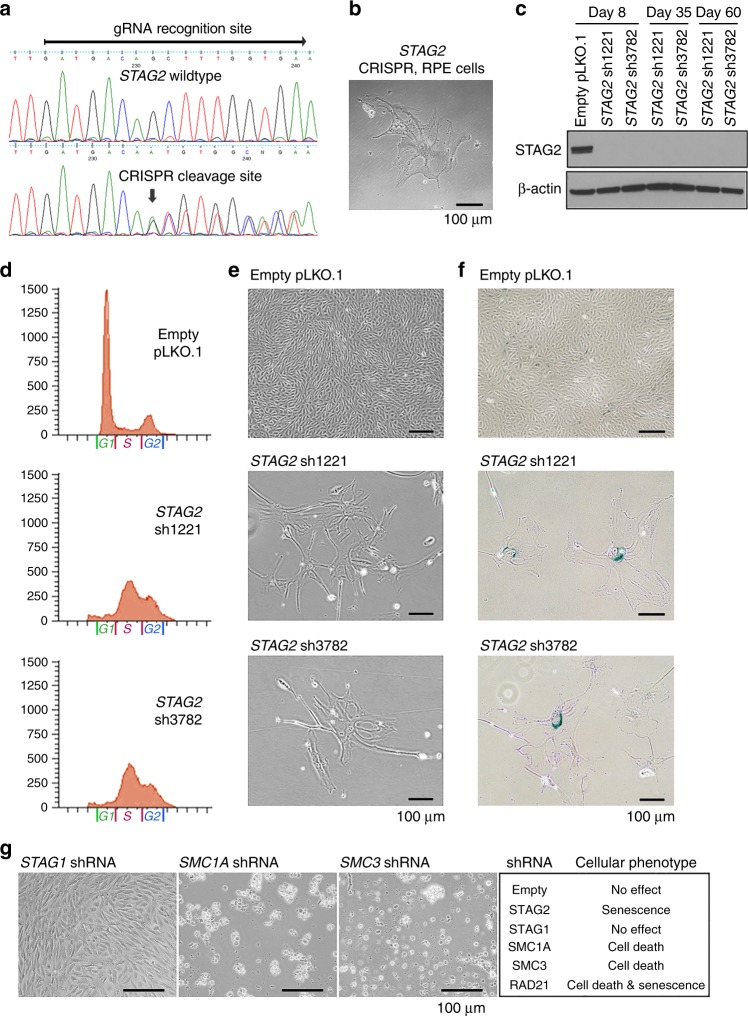
Inactivation of the cohesin regulatory subunit *STAG2* in primary human cells results in intra-S-phase cell cycle arrest and senescence, whereas the core cohesin subunits *SMC1A*, *SMC3*, and *RAD21* are essential for cell viability. **a** Sequence alignment of *STAG2* genomic DNA isolated from RPE cells (top) with wildtype *STAG2* and after Cas9 cleavage with gRNA targeting the *STAG2* locus (bottom). **b** Phase contrast image of RPE cells following ectopic expression of Cas9 and *STAG2* gRNA, which uniformly showed morphologic features of senescence and failure to proliferate. **c** Immunoblot of total lysate from RPE cells following shRNA depletion of STAG2 expression using two independent shRNA sequences (sh1221 and sh3782). **d** Flow cytometry plots of RPE cells following lentiviral transduction with empty pLKO.1 or *STAG2* shRNA demonstrating an abnormal accumulation of cells within S-phase after STAG2 depletion. **e** Phase contrast image of RPE cells following lentiviral transduction with empty pLKO.1 or *STAG2* shRNA demonstrating morphologic features of senescence and failure to proliferate. **f** RPE cells at 14 days following lentiviral transduction with *STAG2* shRNA show β-galactosidase activity characteristic of senescence. **g** Phase contrast image of RPE cells following lentiviral transduction with shRNAs against cohesin components (left), and summary of observed cellular phenotype (right). Source data are provided as a Source Data file

To study the function of *STAG2* in non-transformed human cells, we used the CRISPR/Cas9 system to inactivate the *STAG2* gene in hTERT-immortalized retinal pigmented epithelial (RPE) cells. RPE cells were transfected with an expression vector that encodes Cas9, a guide RNA specific to *STAG2*, and green fluorescent protein (GFP). Following sorting for GFP-positive cells at 48 h after transfection, evidence of Cas9 cleavage was detected by Sanger sequencing (Fig. 1a). All GFP-positive cells failed to proliferate after sorting, and instead displayed morphologic changes associated with cellular senescence (Fig. 1b). This suggested that *STAG2* might be a required gene in primary, non-transformed human cells.

To mechanistically study the requirement for *STAG2* in non-transformed human cells, we performed lentiviral transduction with two independent shRNA sequences against the *STAG2* mRNA on three cell lines: RPE, BJ (an hTERT-immortalized human fibroblast cell line), and SVG p12 (an immortalized human fetal astrocyte cell line). Both shRNA sequences resulted in a sustained depletion of >99% of STAG2 protein (Fig. 1c). Both shRNAs resulted in an accumulation of cells stalled within S-phase of the cell cycle apparent in all three cell lines as early as 48 h after infection, with failure to enter G2/M over an extended time interval (Fig. 1d, Supplementary Fig. 1a, 1b). Assessment of the three cell lines after STAG2 depletion revealed failure to proliferate and morphologic features of cellular senescence including beta-galactosidase activity (Fig. 1e, f, Supplementary Fig. 1c–1e). To further confirm this finding, lentiviral transduction of three additional independent shRNA sequences against the *STAG2* mRNA using the pGIPZ vector system was performed on RPE cells, which resulted in an identical phenotype of intra-S-phase arrest, failure to proliferate, and morphologic features of senescence (Supplementary Fig. 1f–1h). Together, these data indicate that acute *STAG2* inactivation in non-transformed human cells leads to an intra-S-phase cell cycle arrest and senescence.

### Core cohesin ring subunits are essential for cell viability

To assess the cellular phenotypes caused by inactivation of the other cohesin components, we performed lentiviral transduction with two independent shRNAs against each of the core cohesin genes. Both shRNA sequences resulted in >99% depletion of the target proteins (Supplementary Fig. 1i). Depletion of the cohesin ring subunits *SMC1A*, *SMC3*, and *RAD21* in primary human cells resulted in death of the vast majority of cells, with the few surviving *RAD21* shRNA transduced cells showing morphologic features of senescence (Fig. 1g, Supplementary Fig. 1j). In contrast, no decreased proliferation or death was observed following *STAG1* depletion. These results demonstrate that *STAG2* has unique, non-redundant cellular functions compared to *STAG1*, whereas the core cohesin ring subunits are essential for viability in primary non-transformed human cells.

### Inactivation of *STAG2* in primary human cells causes replication fork stalling

**Fig. 2:**
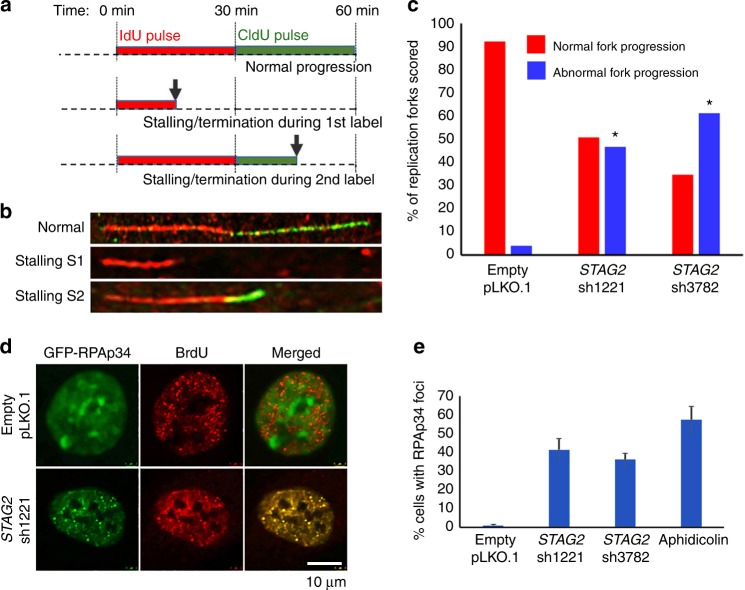
The intra-S-phase cell cycle arrest caused by inactivation of *STAG2* in primary human cells is due to replication fork stalling. **a** Schematic diagram of DNA combing assay to measure DNA replication progression in cultured human cells. Arrows indicate example of DNA fibers with replication stalling or termination. **b** Microscopic images of representative single DNA fibers showing normal replication fork progression (top) and replication fork stalling during IdU pulse (middle) and CldU pulse (bottom). **c** Quantitation of normal and abnormal replication forks scored from DNA combing assay of RPE cells following lentiviral transduction with empty pLKO.1 or two independent *STAG2* shRNAs. More than 200 individual replication forks were evaluated from two independent experiments for each condition. *p < 0.0001, Mann–Whitney unpaired nonparametric *t*-test analysis. **d** Representative images of RPE cells showing accumulation of GFP-tagged RPAp34 at stalled replication forks following lentiviral transduction with *STAG2* shRNA but not empty pLKO.1. **e** Quantitation of RPE cells containing discrete foci of GFP-RPAp34 at stalled replication forks following lentiviral transduction with empty pLKO.1 vector, two independent *STAG2* shRNAs, or following treatment with aphidicolin for 16 h. More than 600 cells were evaluated from three independent experiments for each condition, and error bars show standard error of the mean. Source data are provided as a Source Data file

In order to assess the mechanism of the intra-S-phase cell cycle arrest induced by STAG2 depletion, we established a single fiber DNA replication assay in RPE cells that enables assessment of the progression of individual replication forks (Supplementary Fig. 2a, 2b). Using this methodology, we studied replication fork progression upon STAG2 depletion (Fig. 2a, b). We observed a significant increase in the number of stalled replication forks in cells transduced with two independent *STAG2* shRNAs but not empty vector (Fig. 2c, Supplementary Fig. 2c, 2d). We next assessed localization of the p34 isoform of replication protein A (RPAp34), a single-stranded DNA binding protein that does not visibly accumulate at replication sites under normal S-phase conditions, but focally accumulates at stalled replication forks induced by the DNA polymerase inhibitor aphidicolin or the topoisomerase-II inhibitor etoposide (Supplementary Fig. 2e)^32^. Upon STAG2 depletion, RPAp34 accumulated at stalled replication foci, similar to that observed upon replication block with aphidicolin (Fig. 2d, e, Supplementary Fig. 2f).

### Inactivation of *STAG2* in primary human cells causes disruption of cohesin interaction with the replication machinery

**Fig. 3:**
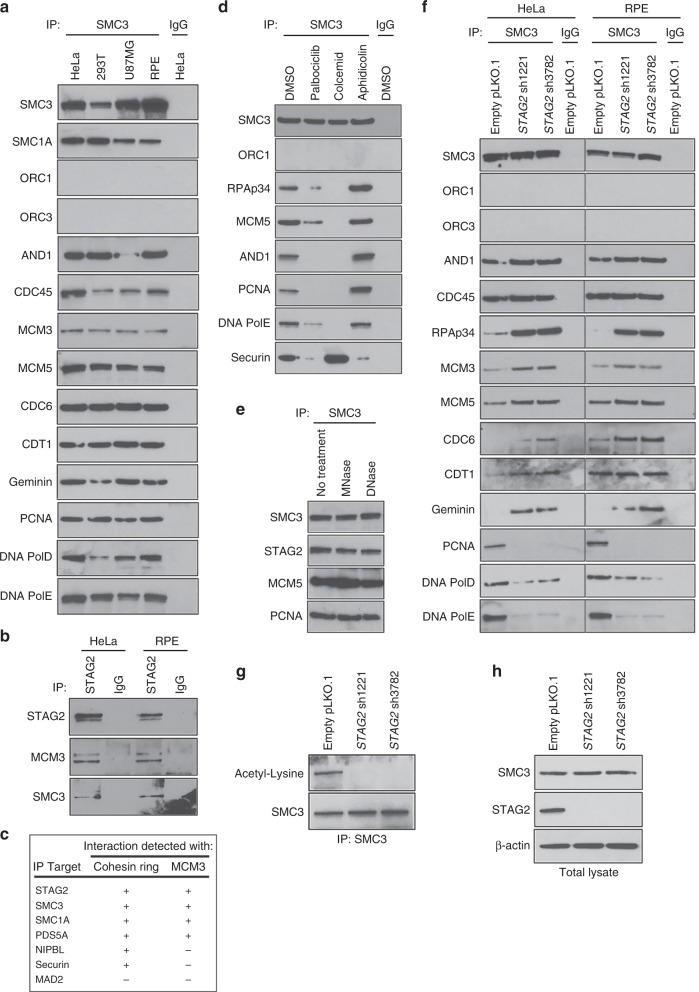
Inactivation of *STAG2* in primary human cells causes disruption of cohesin interaction with the replication machinery along with failure to establish SMC3 acetylation. **a** Immunoprecipitation using antibodies against the cohesin subunit SMC3 pulls down multiple components of the pre-replication complex and replication machinery, but not the origin recognition complex, across a spectrum of human cell lines. **b** Immunoprecipitation using STAG2 antibodies demonstrating interaction of the replication helicase MCM3 with STAG2. **c** Summary of immunoprecipitation results in HeLa and RPE cells demonstrating a specific interaction of MCM3 with the cohesin subunits STAG2, SMC3, SMC1A, and PDS5A, but not the cohesin regulatory factors NIPBL and Securin. **d** Immunoprecipitation using SMC3 antibodies of lysates collected from RPE cells following treatment with DMSO vehicle, the CDK4/6 inhibitor palbociclib, the DNA polymerase inhibitor aphidicolin, and the microtubule polymerization inhibitor colcemid. **e** Immunoprecipitation of lysates from RPE cells using SMC3 antibodies in the presence of DNA nuclease treatment. **f** shRNA depletion of STAG2 disrupts cohesin interaction with the replication factors PCNA, DNA polymerase epsilon, and DNA polymerase delta, while enhancing the interaction with the single-stranded DNA binding protein RPA/p34, the MCM helicase complex, and replication licensing factors (CDC6, CDT1, and Geminin). **g**, **h** Assessment of SMC3 acetylation in RPE cells after lentiviral transduction with empty pLKO.1 or two independent *STAG2* shRNAs. Shown are immunoblots using antibodies against acetylated-lysine following SMC3 immunoprecipitation (**g**), as well as immunoblots of total SMC3 and STAG2 proteins on whole cell lysates (**h**). Source data are provided as a Source Data file

To investigate the function of the cohesin ring at the replication fork, we next assessed for protein-protein interactions between the cohesin subunit SMC3 and the origin recognition complex (ORC1, ORC3), replication licensing factors (CDC45, CDC6, CDT1, and Geminin), the MCM helicase complex (MCM3, MCM5), and the DNA replication machinery (AND1/Ctf4, proliferating cell nuclear antigen [PCNA], DNA polymerase epsilon [PolE], and DNA polymerase delta [PolD]). We identified across a panel of multiple human cell lines that the cohesin ring physically associates with both the pre-replication complex and the replication machinery at the replication fork, but failed to identify an interaction with the origin recognition complex (Fig. 3a, Supplementary Fig. 3a). Next, we performed immunoprecipitation using antibodies against cohesin subunits and cohesin regulatory factors to determine the specificity of interaction with the replication helicase MCM3. We found that MCM3 specifically associates with STAG2, SMC3, SMC1A, and PDS5A, but not the cohesin regulatory factors NIPBL and Securin (Fig. 3b, c, Supplementary Fig. 3b). In order to assess the cell cycle specificity of the interaction between cohesin and replication factors, immunoprecipitation was performed using SMC3 antibodies on RPE cells either asynchronously proliferating (+DMSO vehicle), arrested in G1 phase using the cyclin-dependent kinases 4/6 (CDK4/6) inhibitor palbociclib, arrested in S phase using the DNA polymerase inhibitor aphidicolin, or arrested in mitosis using the microtubule polymerization inhibitor colcemid. The interaction between cohesin and the replication factors was enriched during S-phase arrest, was absent during mitotic arrest, and was diminished during G1 arrest (Fig. 3d). In contrast, SMC3 interaction with Securin, a mitotic regulator of cohesin, was enriched during mitotic arrest and was diminished during G1 and S phase arrest. In order to determine whether the interaction of cohesin with replication factors is indirectly mediated by chromatin association, immunoprecipitation with SMC3 antibodies was performed in the presence of either DNase or MNase treatment (Fig. 3e). These nuclease treatments did not prevent the interaction of SMC3 with either pre-replication factors or the replication machinery (Fig. 3e).

We next studied how *STAG2* inactivation perturbs the association of cohesin with the replication machinery. shRNA depletion of STAG2 caused a complete disruption of cohesin interaction with PCNA, the sliding clamp essential for normal processivity of DNA replication, and also reduced association with DNA polymerases PolD and PolE (Fig. 3f, Supplementary Fig. 3c). In contrast, STAG2 depletion resulted in increased association of cohesin with the single-stranded DNA binding protein RPAp34, and also enhanced binding with the MCM helicase complex and multiple replication licensing factors including Geminin, a negative regulator of DNA replication known to be essential for preventing genome reduplication^33,34^. These data indicate a critical role for the cohesin ring in coordinating the stability and procession of DNA synthesis at the replication fork.

### Inactivation of *STAG2* in primary human cells results in failure to establish SMC3 acetylation

Sister chromatid cohesion is established during S-phase by the cohesin complex, which is known to be governed by acetylation of the cohesin subunit SMC3 by the acetyltransferase enzyme ESCO2^35–39^. Biallelic germline mutations in the *ESCO2* gene are the cause of Roberts syndrome, a cohesinopathy characterized by tetraphocomelia (symmetrical limb reduction), craniofacial anomalies, growth retardation, mental retardation, and cardiac and renal abnormalities (Online Mendelian Inheritance in Man #268300). We therefore assessed whether the DNA replication fork stalling induced by STAG2 deficiency may be due to impaired SMC3 acetylation. shRNA depletion of STAG2 in RPE cells abolished SMC3 acetylation (Fig. 3g, h), indicating that STAG2 is required for this critical post-translational modification of the cohesin ring during DNA replication.

### *STAG2* absence leads to replication fork collapse, DNA double-strand breaks, and DNA damage checkpoint activation

**Fig. 4:**
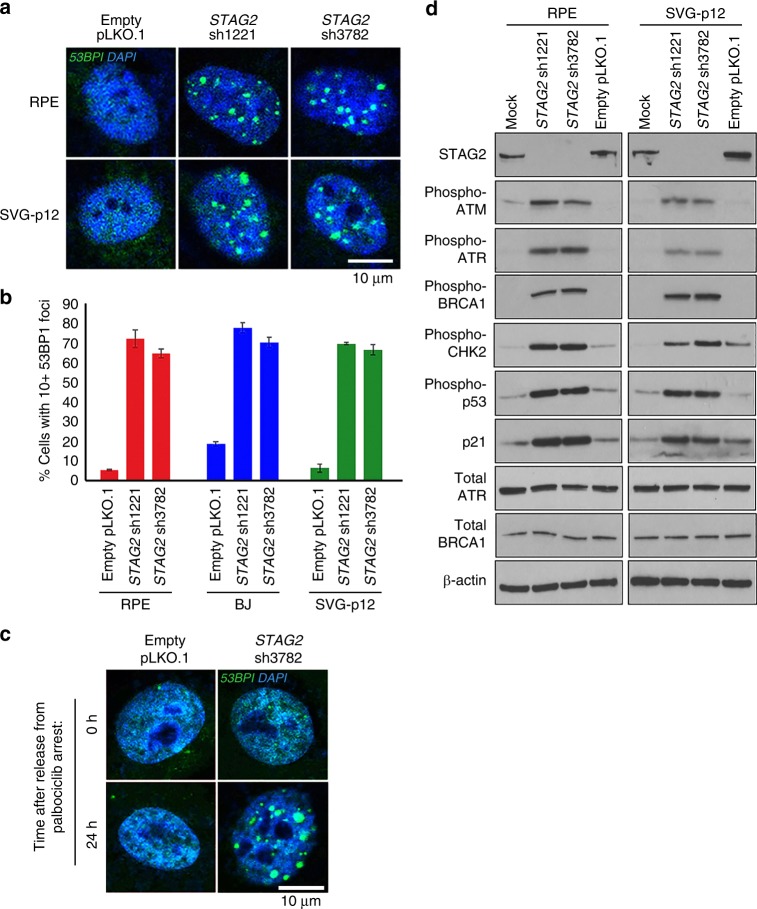
The replication fork stalling caused by *STAG2* depletion leads to fork collapse, DNA double-strand breaks, and DNA damage checkpoint activation. **a** Representative immunofluorescence images of RPE and SVG p12 cells following lentiviral transduction with empty pLKO.1 or two independent *STAG2* shRNAs showing spontaneous accumulation of 53BP1 foci. **b** Quantitation of RPE, BJ, and SVG p12 cells with greater than ten 53BP1 foci following lentiviral transduction with empty pLKO.1 or two independent *STAG2* shRNAs. Approximately 2000 cells were evaluated from two independent experiments for each condition, and error bars show standard error of the mean. **c** Representative immunofluorescence images demonstrating that 53BP1 foci formation after STAG2 depletion is dependent on cell cycle proliferation. Cells were arrested in G1 phase of the cell cycle using palbociclib, followed by lentiviral transduction with empty pLKO.1 or *STAG2* shRNA. No 53BP1 foci formation was observed until release from palbociclib arrest. **d** Immunoblots of total lysate from RPE and SVG p12 cells for DNA damage response proteins following lentiviral transduction with empty pLKO.1 or two independent *STAG2* shRNAs. Source data are provided as a Source Data file

To evaluate the consequence of replication fork stalling induced by *STAG2* inactivation, we investigated whether this may lead to replication fork collapse and DNA double-strand breaks. We observed accumulation of 53BP1 foci, a marker of DNA double-strand breaks but not normal replication sites, in the three non-transformed human cell lines upon shRNA depletion of STAG2 (Fig. 4a, b). In order to assess whether 53BP1 foci were resulting from replication fork collapse, we performed shRNA depletion on cells arrested in G1 phase of the cell cycle using the CDK4/6 inhibitor palbociclib (Supplementary Fig. 4a, 4b). No 53BP1 foci were observed in cells arrested in G1 phase of the cell cycle; however, upon release from palbociclib arrest, STAG2-depleted cells developed 53BP1 foci within a few hours, indicating that these most likely result from collapsed replication forks after entry into S-phase (Fig. 4c, Supplementary Fig. 4c). Commensurate with induction of 53BP1 foci accumulation, STAG2 depletion in multiple non-transformed human cell lines resulted in activation of DNA damage checkpoint signaling, as evidenced by a robust increase in phosphorylated isoforms of ATM, ATR, BRCA1, Chk2, and p53 proteins, as well as upregulation of the cell cycle checkpoint inhibitor p21^WAF1/CIP1^ (Fig. 4d). Together, these data indicate that acute inactivation of STAG2 in non-transformed human cells leads to induction of DNA double-strand breaks likely due to replication fork collapse that results in activation of DNA damage checkpoint signaling.

### The intra-S-phase cell cycle arrest but not the loss of SMC3 acetylation induced by *STAG2* inactivation is p53 dependent

**Fig. 5:**
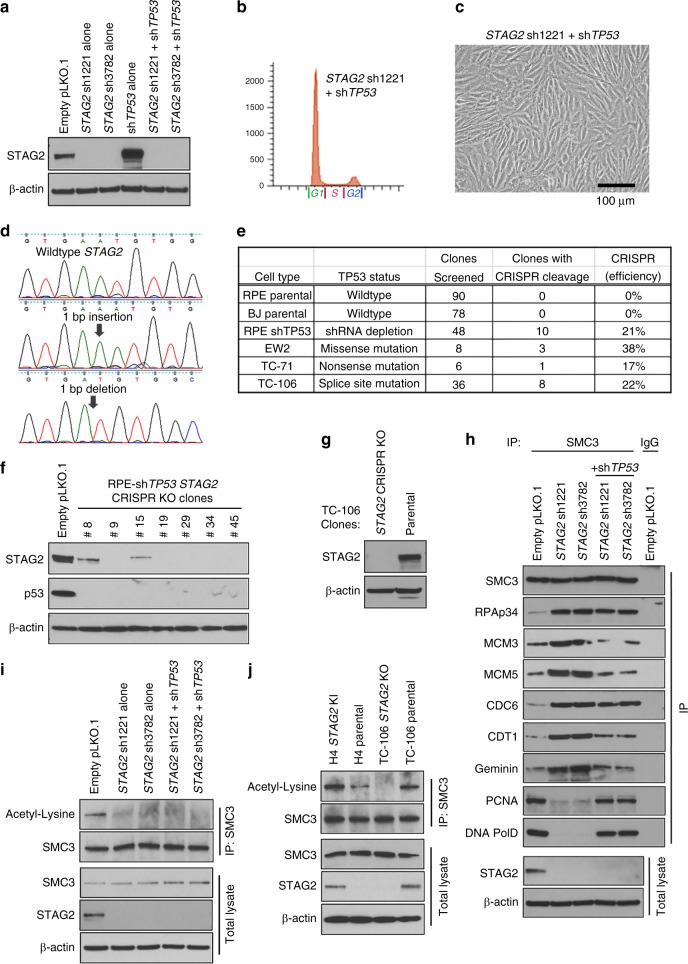
The intra-S-phase cell cycle arrest but not the loss of SMC3 acetylation induced by *STAG2* inactivation is p53 dependent. **a** Immunoblot of total lysate from RPE cells following lentiviral *STAG2* shRNA depletion either alone or in combination with *TP53* shRNA depletion. **b** Flow cytometry plot of RPE cells following lentiviral transduction with *STAG2* shRNA in combination with *TP53* shRNA demonstrating bypass of the S-phase arrest induced by STAG2 depletion alone. **c** Phase contrast image of RPE cells following lentiviral transduction with *STAG2* shRNA in combination with *TP53* shRNA demonstrating cellular proliferation and abrogation of the senescence induced by STAG2 depletion alone. **d** Sequence alignment of representative *STAG2* knockout clones that were readily obtained following ectopic expression of Cas9 and *STAG2* gRNA in RPE cells subsequent to *TP53* shRNA depletion. **e** Results of *STAG2* knockout clone generation following ectopic expression of Cas9 and *STAG2* gRNA in multiple human cell lines with wildtype *TP53*, *TP53* shRNA depletion, or mutant *TP53* alleles. **f** Immunoblots of total lysate from several of the *STAG2* knockout clones generated by Cas9 cleavage in RPE cells with *TP53* shRNA depletion. **g** Immunoblots of total lysate from the *STAG2* knockout clone generated by Cas9 cleavage in TC-106 Ewing sarcoma cells harboring *TP53* splice site mutation. **h** *TP53* inactivation via lentiviral shRNA depletion in STAG2-depleted RPE cells restores the interaction of cohesin with the replication factors PCNA, PolE, and PolD, as well as blocking the enhanced binding of cohesin with MCM3, MCM5, CDT1, and Geminin. However, *TP53* inactivation does not block the enhanced interaction of cohesin with RPAp34 or CDC6. **i** Combined *STAG2* and *TP53* shRNA depletion enables bypass of S-phase cell cycle arrest independent of SMC3 acetylation. Shown are immunoblots using antibodies against acetylated-lysine following SMC3 immunoprecipitation, as well as immunoblots of total SMC3 and STAG2 on whole cell lysates of RPE cells after lentiviral shRNA transduction. **j** Impaired SMC3 acetylation is present in H4 glioblastoma cells with *STAG2* truncating frameshift mutation and TC-106 Ewing sarcoma cells after *STAG2* knockout by targeted Cas9 cleavage. Source data are provided as a Source Data file

While acute inactivation of *STAG2* in non-transformed human cells results in cell cycle arrest, *STAG2* homozygous deletion or inactivating truncating mutations are commonly selected for during the development of glioblastoma, urothelial carcinoma, Ewing sarcoma, acute myeloid leukemia, and other human cancer types, whereby this genetic inactivation likely functions to promote some aspect of tumor survival or growth. Multiple studies have noted a strong association of *STAG2* inactivation and *TP53* mutation in Ewing sarcoma, whereby the combination of these two genetic alterations is significantly associated with poor prognosis relative to *STAG2* and *TP53* wildtype tumors^24,28^. We thus speculated that p53 function may be an important modulator of the cellular senescence phenotype seen in non-transformed cells versus the pro-tumorigenic phenotype seen in human cancers with *STAG2* inactivation. Therefore, we tested the effects of dual depletion of *STAG2* and *TP53* in hTERT-immortalized RPE cells (Fig. 5a). We observed a bypass of the intra-S-phase cell cycle arrest and cellular senescence induced by *STAG2* inactivation alone (Fig. 5b, c, Supplementary Fig. 5). We therefore anticipated being able to successfully knockout *STAG2* in *TP53*-depleted primary human cells, as well as *TP53*-mutant human cancer cell lines. Indeed, using the same CRISPR mediated gene targeting approach as performed on parental RPE cells that failed to produce knockout clones (Fig. 1a, b), we were able to produce *STAG2* knockout clones at high efficiency in p53-depleted RPE cells and multiple Ewing sarcoma cell lines harboring inactivating *TP53* mutations (Fig. 5d–g). These results demonstrate that the intra-S-phase cell cycle arrest induced by *STAG2* inactivation is p53 dependent and help to explain the frequent co-occurrence of *STAG2* and *TP53* mutation in Ewing sarcoma.

In order the explore the mechanism by which *TP53* inactivation allows bypass of the intra-S-phase arrest in *STAG2* mutant cells, we performed immunoprecipitation reactions using SMC3 antibodies to study the binding of cohesin with the DNA replication machinery in STAG2-depleted RPE cells with and without simultaneous shRNA knockdown of p53. Simultaneous knockdown of p53 expression restored the interaction of cohesin with the replication factors PCNA, PolE, and PolD, as well as prevented the enhanced binding of cohesin with MCM3, MCM5, CDT1, and Geminin (Fig. 5h). However, simultaneous *TP53* knockdown did not block the enhanced interaction of cohesin with RPAp34 or CDC6. Thus, while p53 is a critical regulator of replication fork procession in *STAG2* mutant cells, there are perturbations of the interaction between cohesin and replication factors (e.g., RPAp34 and CDC6) that are independent of p53 control.

As we observed disruption of SMC3 acetylation following *STAG2* depletion in *TP53*-wildtype cells that is known to be required for establishment of sister chromatid cohesion during DNA replication, we investigated the status of SMC3 acetylation in RPE cells after dual depletion of *STAG2* and *TP53*, as well as in paired isogenic *STAG2* glioblastoma (H4) and Ewing sarcoma (TC-106) cell lines. We have previously used homologous recombination to correct the endogenous *STAG2* mutant allele in the H4 human glioblastoma cell line^26^. We observed that concomitant depletion of *TP53* was not sufficient to restore the abrogation of SMC3 acetylation in RPE cells (Fig. 5i). Additionally, we found that the levels of SMC3 acetylation were impaired in cancer cells lines harboring *STAG2* inactivation (H4 parental and TC-106 *STAG2* knockout cells) relative to their *STAG2* wildtype counterparts (H4 *STAG2* knock-in and TC-106 parental cells) (Fig. 5j). These results indicate that STAG2 is critical for SMC3 acetylation independent of p53 function, and that cancer cells with concomitant *STAG2* and *TP53* inactivation escape cellular senescence despite abrogation of SMC3 acetylation.

### STAG2 deficiency in human cancer cells creates a synthetic lethality with *STAG1*, whereas the other core cohesin subunits are required independent of *STAG2* status

**Fig. 6:**
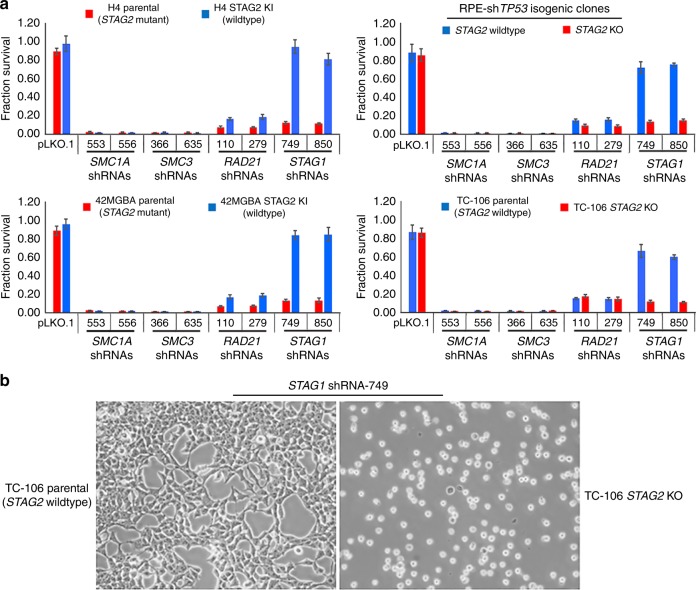
*STAG2* deficiency in human cancer cells creates a synthetic lethality with *STAG1*, whereas the core cohesin subunits *SMC3*, *SMC1A*, and *RAD21* are required genes in human cancer cells independent of *STAG2* status. **a** Quantitation of survival from lentiviral shRNA depletion of core cohesin subunits in four pairs of *STAG2* isogenic human cells (H4 glioblastoma, 42MGBA glioblastoma, TC-106 Ewing sarcoma, and RPE sh*TP53*). Each data point is the mean of 8 replicates from 2 independent experiments, and error bars show standard error of the mean. **b** Representative phase contrast images of *STAG2* isogenic TC-106 Ewing sarcoma cells showing synthetic lethality of *STAG2* inactivation with shRNA depletion of *STAG1*

Two recent studies have both demonstrated that *STAG2* mutant cancer cells have a critical dependence on *STAG1*^40,41^. We observed no significant growth inhibition or cell death upon lentiviral shRNA knockdown of >99% of STAG1 protein in hTERT-immortalized primary human cells with wildtype *STAG2*, whereas shRNA knockdown of >99% of SMC3, SMC1A, and RAD21 proteins resulted in cell death (Fig. 1g, Supplementary Fig. 1i, 1j). In order to investigate the genetic dependencies for these core cohesin genes in cancer cells harboring inactivating *STAG2* mutations, lentiviral shRNA transduction using two independent shRNA sequences for each cohesin subunit was performed in four pairs of isogenic *STAG2* human cell lines (H4 glioblastoma, 42MGBA glioblastoma, TC-106 Ewing sarcoma, and RPE primary epithelial cells with hTERT overexpression and *TP53* depletion). In keeping with the two recent studies, STAG1 depletion had no significant growth inhibition on STAG2 proficient cells, whereas STAG1 depletion uniformly resulted in cell death across all four of the STAG2 deficient cells (Fig. 6a, b, Supplementary Fig. 6). In contrast, SMC3, SMC1A, and RAD21 were essential genes in all four isogenic pairs, with cell death uniformly observed in both STAG2 proficient and deficient cells (Fig. 6a).

### *STAG2* deficiency in human cancer cells creates a synthetic lethality with DNA double-strand break repair factors

**Fig. 7:**
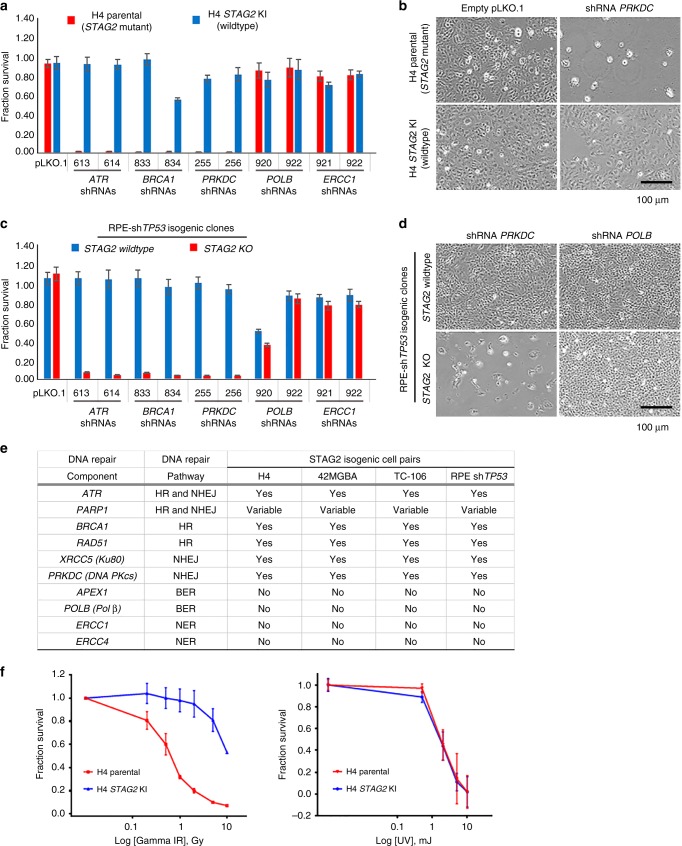
*STAG2* deficiency in human cancer cells creates a synthetic lethality with DNA double-strand break repair factors and increased sensitivity to ionizing radiation. **a** Quantitation of survival from shRNA synthetic lethality screen in *STAG2* isogenic H4 glioblastoma cells (parental = *STAG2* mutant, *STAG2* KI = wildtype). Each data point is the mean of 8 replicates from 2 independent experiments, and error bars show standard error of the mean. **b** Representative phase contrast images of *STAG2* isogenic H4 glioblastoma cells showing synthetic lethality of STAG2 inactivation with shRNA depletion of the non-homologous end joining factor DNA-PKcs. **c** Quantitation of survival from shRNA synthetic lethality screen in *STAG2* isogenic RPE sh*TP53* cells. Each data point is the mean of 8 replicates from 2 independent experiments, and error bars show standard error of the mean. **d** Representative phase contrast images of *STAG2* isogenic RPE sh*TP53* cells showing synthetic lethality of *STAG2* knockout with shRNA depletion of the non-homologous end joining factor DNA-PKcs, but not the base excision repair factor DNA polymerase β. **e** Summary of results from shRNA synthetic lethality screen in four pairs of *STAG2* isogenic human cells (H4 glioblastoma, 42MGBA glioblastoma, TC-106 Ewing sarcoma, and RPE hTERT sh*TP53*). HR, homologous recombination. NHEJ, non-homologous end joining. BER, base excision repair. NER, nucleotide excision repair. **f** Survival plots of *STAG2* isogenic H4 glioblastoma cells (parental = *STAG2* mutant, *STAG2* KI = wildtype) following gamma irradiation or ultraviolet irradiation. Each data point is the mean of 12 replicates from 3 independent experiments, and error bars show standard error of the mean. Source data are provided as a Source Data file

Given the large burden of collapsed replication forks and DNA double-strand breaks in *STAG2* deficient cells, we hypothesized that *STAG2* mutational inactivation might create a synthetic lethality with factors critical for DNA damage repair. We thus performed an shRNA-based synthetic lethality screen in the four pairs of isogenic *STAG2* human cell lines, which included genes known to be essential for homologous recombination (HR), non-homologous end-joining (NHEJ), base excision repair (BER), and nucleotide excision repair (NER). We first validated efficient shRNA depletion of each of the ten DNA repair genes and selected two independent hairpin sequences that produced >90% knockdown for performing the screen (Supplementary Fig. 7a). Across all four isogenic pairs, STAG2-deficiency correlated with a significant decrease in survival following shRNA depletion of genes responsible for DNA double-strand break repair via either HR or NHEJ (e.g., *ATR*, *BRCA1*, *RAD51*, *XRCC5*, and *PRKDC*) relative to their isogenic STAG2-proficient counterparts (Fig. 7a–e, Supplementary Fig. 7b–7f). In contrast, no significant difference in survival was observed in the four isogenic pairs following shRNA depletion of genes responsible for BER and NER (e.g., *APEX1*, *POLB*, *ERCC1*, and *ERCC4*). We observed a variable difference in survival using shRNA against *PARP1*, which encodes a poly-ADP-ribosyltransferase critical for DNA double-strand break repair, that may be due to insufficient shRNA depletion or possibly redundancy with additional PARP isoforms encoded in the human genome. Together, these results highlight a critical dependency for DNA double-strand break repair, but not excision repair, in *STAG2* mutant cells.

Given this finding, we speculated that *STAG2* mutant cells may show increased sensitivity to ionizing radiation via synergizing with the replication fork disruption to cause a catastrophic accumulation of DNA double-strand breaks. Four pairs of isogenic *STAG2* human cells were subjected to varying doses of either gamma-irradiation or ultraviolet irradiation, and the effects on cellular survival were assessed. Across all four isogenic pairs, STAG2-deficiency correlated with a significant decrease in survival following gamma-radiation compared to their isogenic *STAG2*-proficient counterparts (Fig. 7f, Supplementary Fig. 7g). In contrast, no significant difference in survival was observed in the four isogenic pairs following ultraviolet irradiation (Fig. 7f, Supplementary Fig. 7h). These results further highlight the dependency for DNA double-strand break repair, but not excision repair, in *STAG2* mutant cells.

### *STAG2* deficient cancer cells harbor increased sensitivity to cytotoxic chemotherapeutic agents and inhibitors of DNA double-strand break repair

**Fig. 8:**
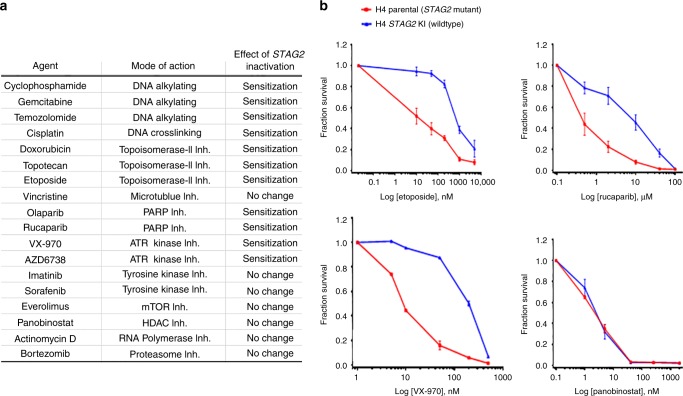
*STAG2* deficient cancer cells harbor increased sensitivity to cytotoxic chemotherapeutic agents and small molecule inhibitors of DNA double-strand break repair. **a** Summary of results from chemotherapeutic screen in four pairs of *STAG2* isogenic human cells (H4 glioblastoma, 42MGBA glioblastoma, TC-106 Ewing sarcoma, and RPE hTERT sh*TP53*). All four pairs showed similar sensitivities for all agents. **b** Survival plots of *STAG2* isogenic H4 glioblastoma cells following treatment with etoposide (topoisomerase II inhibitor), rucaparib (PARP inhibitor), VX-970 (ATR inhibitor), and panobinostat (HDAC inhibitor). Each data point is the mean of 12 replicates from 3 independent experiments, and error bars show standard error of the mean. Source data are provided as a Source Data file

While *STAG2* mutational activation is known to be frequent in glioblastoma, Ewing sarcoma, and urothelial carcinoma, the effect of *STAG2* status on the response to cytotoxic chemotherapy agents routinely used in the treatment of these cancer types is largely unknown. We therefore investigated the response of four isogenic STAG2 human cell pairs (two glioblastoma, one Ewing sarcoma, and one hTERT-immortalized epithelial cell line) to a panel of commonly used cytotoxic chemotherapy agents and small molecule inhibitors with various mechanisms of action. We observed that STAG2-deficiency across all four isogenic pairs correlated with a significant decrease in survival following treatment with DNA alkylating agents, DNA crosslinking agents, topoisomerase inhibitors, PARP inhibitors, and ATR inhibitors (Fig. 8a, b, Supplementary Fig. 8a–8d). In contrast, no significant difference in survival was observed in the four isogenic pairs following treatment with inhibitors of tyrosine kinases, 26S proteasome, RNA polymerase, mTOR, histone deacetylase (HDAC), and microtubule polymerization. To further confirm the differential sensitivity of STAG2-proficient versus deficient cancer cells to DNA damaging chemotherapeutic agents, we performed clonogenic survival assays with the DNA alkylating agent cyclophosphamide. Cyclophosphamide treatment significantly reduced colony formation in *STAG2* mutant cells relative to their *STAG2* wildtype counterparts in all four isogenic pairs (Supplementary Fig. 8e, 8f).

## Discussion

Subunits of the cohesin complex have emerged as frequent targets of somatic mutation in human tumors over the past decade of cancer genomics research. However, methods to therapeutically target cohesin-mutant cancers are currently unknown. Herein we have identified an essential role for the *STAG2* gene in stability of the replication fork that creates a targetable synthetic lethality in *STAG2* mutant cancers.

In order to study the fundamental cellular processes controlled by *STAG2*, we used multiple genetic mechanisms to inactivate *STAG2* function in primary non-transformed human cells. Both CRISPR knockout and shRNA depletion revealed that *STAG2* is required for cellular proliferation but not cell viability across a range of normal human cell types including epithelial cells, fibroblasts, and glial cells. In contrast, inactivation of the cohesin ring subunits *SMC1A*, *SMC3*, and *RAD21* is not compatible with viability in both primary non-transformed human cells and human cancer cell lines. The reason why cohesin mutations in human cancers most commonly affect *STAG2* and less commonly the other cohesin subunits has been unknown. We and others have speculated that this may be in part because the *STAG2* gene is on the X chromosome and therefore requires only a single genetic event in order to achieve loss of function, whereas the other cohesin subunits located on the autosomes require two genetic events. However, our findings indicate that truncating mutations in cohesin ring subunits are uncommon in human cancers because these are required genes for cell viability. In contrast, truncating loss of function mutations in *STAG2* are likely selected for in human cancers because *STAG2* inactivation is not an essential gene, but instead is a critical regulator of cohesin function that when subverted promotes tumorigenesis without perturbing the essential functions of the core cohesin ring.

**Fig. 9:**
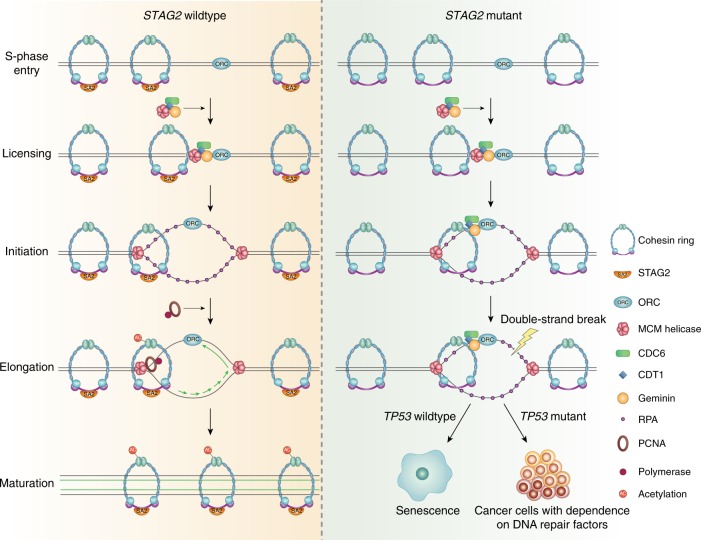
Model depicting the role of the cohesin complex during replication fork procession. In normal cells with intact *STAG2* (left), cohesin dynamically associates with the pre-replication complex and replication machinery to stabilize the replication fork. In cancer cells with *STAG2* inactivation (right), disruption of the cohesin interaction with PCNA and DNA polymerases results in failure to establish SMC3 acetylation, replication fork collapse, DNA double-strand breaks, and enhanced sensitivity to DNA damaging chemotherapeutic agents

Our studies reveal that STAG2 is a critical mediator at the replication fork that governs interaction of the cohesin ring with the replication machinery. STAG2 deficiency in primary non-transformed human cells induces an intra-S-phase arrest due to stalling of replication forks. We demonstrate across a spectrum of human cell lines that the cohesin ring physically associates with a multitude of key replication factors including the MCM helicase complex, replication licensing factors such as Geminin and CDT1, and the replisome machinery including PCNA and DNA polymerases. In the absence of STAG2, the cohesin ring is more robustly bound to the replication licensing inhibitor Geminin, the single-strand DNA binding protein RPA2/p34, and the MCM helicase complex. In contrast, the interaction of the cohesin ring with replisome components PCNA, PolD, and PolE is disrupted, whereas the interaction with other replisome components including CDC45 and AND1 remains stable. These findings indicate that the interaction of the cohesin ring with the pre-replication complex and the active replication machinery during S phase is a complex and dynamic process, which is perturbed by the absence of STAG2. We speculate that the binding of cohesin with Geminin may represent a critical mediator of an intra-S-phase checkpoint to ensure establishment of sister chromatid cohesion during DNA replication. The disruption of cohesin binding with the replication factors PCNA, PolD, and PolE upon STAG2 depletion may represent either lack of recruitment to the active replication fork or instability and dissociation from the stalled replication fork. Regardless of the mechanism, this results in stalling of DNA synthesis, accumulation of single-stranded DNA binding protein RPA2/p34 at replication sites, and eventually fork collapse (Fig. 9).

We find that STAG2 is required to establish acetylation of SMC3 during DNA replication, a post-translational modification that has been reported as being critical for efficient DNA replication^39^. However, we show that SMC3 acetylation is actually dispensable for DNA replication, as concurrent *STAG2* and *TP53* inactivation results in a bypass of the intra-S-phase arrest despite failure to establish SMC3 acetylation. These results show that SMC3 acetylation is not required for DNA replication in cancer cells with *TP53* mutation, but instead suggest that SMC3 acetylation is critical for controlling activation of an S-phase checkpoint that ensures establishment of sister chromatid cohesion during DNA synthesis. These results also help to explain the frequent co-occurrence of *STAG2* and *TP53* mutations in human cancers including Ewing sarcoma^24,28^. *STAG2* mutation disrupts stability of the replication fork leading to fork collapse and DNA double-strand breaks, which in normal cells with intact *TP53* leads to checkpoint activation and senescence. However, in cells with concurrent *TP53* inactivation, the fork collapse and DNA double-strand breaks induced by STAG2 deficiency likely promotes tumorigenesis by facilitating oncogenic structural variants such as gene amplifications, deletions, and rearrangements. Indeed, an increased quantity of chromosomal structural variants is found in *STAG2* mutant versus *STAG2* wildtype Ewing sarcomas^28,42^.

We find that a consequence of the replication fork instability caused by *STAG2* inactivation in human cells is a critical dependence on DNA double-strand break repair factors. STAG2 deficient cells show profound sensitivity to depletion of genes involved in homologous recombination and non-homologous end-joining, but not excision repair pathways. Additionally, we find that *STAG2* mutant cancer cells have increased sensitivity to ionizing radiation and cytotoxic chemotherapeutic agents (e.g., cyclophosphamide and etoposide) that function by inducing DNA double-strand breaks. Furthermore, PARP1 or ATR inhibition constitutes a STAG2 synthetic lethal interaction that can be elicited with clinical inhibitors such as olaparib or AZD6738, which are currently FDA-approved and in phase II clinical trials, respectively. These effects were robust across isogenic cell models of different tumor types, both glioblastoma and Ewing sarcoma, as well as an immortalized epithelial cell line. These findings have significant treatment implications for *STAG2* mutant cancers, suggesting a greater benefit from radiation therapy and chemotherapeutic agents that induce DNA double-strand breaks.

In summary, *STAG2* mutations are a frequent characteristic of a few of the most common human cancers including glioblastoma, urothelial carcinoma, and Ewing sarcoma. Here we show that cohesin has critical functions at the DNA replication fork regulated by STAG2, and that the replication fork instability caused by *STAG2* inactivation creates a targetable synthetic lethality in cohesin-mutant cancers. Together with other recent studies showing enhanced PARP inhibitor sensitivity in cohesin-deficient cells^43,44^, these studies lay the foundation for clinical trials of targeted agents in *STAG2* mutant cancers.

## Methods

### Human cell lines and culture conditions

The list of human cell lines used in this study and their source is provided in Supplementary Table 1. All cell lines were cultured in DMEM supplemented with 10% fetal bovine serum and 1% penicillin/streptomycin. Cell cultures were maintained at 37 °C in a humidified incubator with 5% carbon dioxide. H4 and 42MGBA human glioblastoma cell lines harboring endogenous truncating mutations in the *STAG2* gene and their isogenic *STAG2* knock-in counterparts in which the mutant alleles have been corrected to wildtype status by homologous recombination were previously described^26^.

### Lentiviral shRNA constructs and CRISPR guide RNA

All lentiviral shRNA expression plasmids were obtained from Dharmacon that had been generated by The RNAi Consortium (TRC) in the pLKO.1-Puro lentiviral expression vector or in the pGIPZ lentiviral expression vector. Sequence of the short hairpin RNAs are listed in Supplementary Table 2. Five independent shRNA clones were used for *STAG2*, five independent shRNA clones were tested for each of the cohesin components, and five independent shRNA clones were tested for each of the ten DNA repair factor genes included in the synthetic lethality screen. shRNA clones were received from Dharmacon as stabs in Luria agar, which were then cultured on Luria agar plates containing 100 μg/mL ampicillin. Single colonies were isolated for plasmid preparation (Qiagen). Sanger sequencing confirmation was performed over the hairpin sequence for all lentiviral shRNA expression constructs. A guide RNA against *STAG2* that had been cloned into the BamHI restriction site of pCas-Guide-EF1a-GFP construct was obtained from OriGene. Sequence of the *STAG2* guide RNA and sequencing primers are listed in Supplementary Table 2. Sanger sequencing confirmation was performed over the guide RNA sequence after plasmid preparation.

### CRISPR induced mutagenesis of the *STAG2* gene in human cell lines

hTERT-immortalized RPE cells, hTERT-immortalized RPE sh*TP53* cells, hTERT-immortalized BJ cells, EW2 Ewing sarcoma cells, TC-71 Ewing sarcoma cells, and TC-106 Ewing sarcoma cells were used for CRISPR induced mutagenesis of the *STAG2* gene. Exponentially proliferating cells were transfected with the pCas-STAG2 gRNA-EF1a-GFP construct using FuGENE 6 according to the manufacturer’s protocol. At 48 h post-transfection, cells were collected by trypsinization and GFP-positive cells were sorted using a Sony SH800 Cell Sorter. Sorted cells were diluted, and single GFP-positive cells were placed into wells of a 96-well plate for isolation of single cell clones. Single cell clones were expanded, and a subset of the cells were used for genomic DNA isolation for assessment of *STAG2* gene status. Genomic DNA was isolated via proteinase K digestion, phenol-chloroform extraction, and subsequent ethanol precipitation. Primers were used to amplify a portion of the *STAG2* gene that included the sequence targeted by the guide RNA using the MyFi DNA Polymerase Kit, and Sanger sequencing using BigDye terminators was performed to visualize Cas9 cleavage. CRISPR efficiency was assessed as the number of clones with evidence of Cas9 cleavage divided by the total number of clones screened. Cell lysate was prepared from individual clones found to have Cas9 induced frameshift mutations predicted to cause premature truncation of the encoded STAG2 protein, and immunoblots were performed using a monoclonal antibody raised against an epitope at the C-terminus of the STAG2 protein (clone J-12, Santa Cruz sc-81852) as described in detail below.

### Lentivirus production and infection for shRNA transduction

Empty pLKO.1 vector or gene specific pLKO.1-shRNA vectors were co-transfected into 293T cells with pVSV-G and pCMV 8.2-deltaR helper plasmids using FuGENE 6 according to the manufacturer’s protocol. Empty pGIPZ vector or pGIPZ-*STAG2* shRNA vectors were co-transfected into 293T cells with the Dharmacon Trans-Lentiviral Packaging Plasmid System using FuGENE 6 according to the manufacturer’s protocol. Virus-containing conditioned medium was harvested 48 h after transfection, filtered, and used to infect recipient cells in the presence of 5 µg/mL polybrene.

### Flow cytometric analysis of cell cycle

RPE, BJ, and SVG p12 cells either synchronized with drugs or treated with lentiviral shRNA conditioned media were collected by trypsinization and washed with PBS. Cells were re-suspended and fixed with 70% ethanol at 4 °C for 1 h. Fixed cells were washed in PBS and stained with propidium iodide (50 μg/mL) containing RNAse (100 μg/mL) for 30 min in the dark. Fifteen thousand stained cells per experimental condition were assessed using a BD LSRFortessa cell analyzer and BD FACSDiva software (BD Biosciences). The following drugs were used: DMSO vehicle, aphidicolin (2 μg/mL, for 48 h), palbociclib (1 μM, for 48 h), colcemid (0.5 μg/mL, for 16 h). Flow cytometric analysis was performed following treatment with lentiviral shRNAs at multiple time points as indicated.

### Immunoprecipitation and Western blotting

Total lysate was harvested from experimental cells in RIPA buffer (Pierce) supplemented with protease inhibitor cocktail (Pierce) at 4 °C. Lysate was centrifuged at 16,000 × *g* to remove insoluble material. Protein concentration of the total soluble lysates were measured using BCA protein assay kit (Pierce). For immunoprecipitation, cells were harvested in IP Lysis buffer (Pierce) supplemented with protease and phosphatase inhibitor cocktail (Pierce) at 4 °C. For evaluation of SMC3 acetylation, the IP lysis buffer was supplemented with 5 mM sodium butyrate (a deacetylase inhibitor). Protein-A sepharose beads (Abcam) were blocked in 0.1% BSA for 1 h and then incubated with the indicated primary antibodies for 1 hour at 4 °C with gentle mixing. Antibody-conjugated beads were then centrifuged and washed with cell lysis buffer before adding to the cell lysate. Immunoprecipitation was performed overnight with gentle mixing at 4 °C, followed by centrifugation and washing of the beads with lysis buffer. A subset of the immunoprecipitation reactions were treated with DNase I (New England Biolabs, 10 units used per immunoprecipitation reaction) or micrococcal nuclease (Thermo Scientific, 500 units used per immunoprecipitation reaction) for 2 h at room temperature prior to washing of the beads with lysis buffer.

Total cell lysates and immunoprecipitates were denatured with Bolt Sample Reducing Agent in NuPAGE LDS Sample Buffer at 94 ^o^C for 10 min. Poly-acrylamide gel electrophoresis was performed using Bolt 4–12% Bis-Tris Plus gradient gels. Resolved proteins were transferred onto nitrocellulose membranes using iBlot2 NC Transfer Stacks and iBlot2 Gel Transfer Device according to the manufacturer’s protocol (Life Technologies). Primary and HRP-conjugated secondary antibodies were diluted with iBind Flex Solution. The iBind Flex chromatography system (Life Technologies) was used for all Western blots. Chemiluminescence was performed using SuperSignal West Pico PLUS Chemiluminescent Substrate for 2 min. Chemiluminescence signal was recorded on Xposure film (Thermo Scientific) followed by development in X-ray film processer (JP-33, JPI Healthcare). The list of antibodies used for immunoprecipitation and Western blotting and their source is provided in Supplementary Table 3.

### Immunofluorescence and microscopy

For 53BP1 immunofluorescence, cells were seeded on glass coverslips and irradiated or transduced with lentiviral shRNAs after 24 h. Cells were fixed at the indicated time points following gamma-radiation and at 48 h after lentiviral shRNA transduction. Cells were fixed with 10% neutral-buffered formalin, permeabilized with 0.2% Triton X-100, pre-incubated with 10% BSA in PBS, and then incubated with 53BP1 primary antibody overnight at 4 °C. Cover slips were washed, incubated with fluorescent-conjugated secondary antibodies (Molecular Probes) for 2 h at room temperature, followed by counterstaining and mounting on glass slides with Vectashield Mounting Medium containing DAPI (Vector Labs). The list of antibodies used for immunofluorescence and their source is provided in Supplementary Table 3.

Nuclear localization of the p34 subunit of replication protein A (RPAp34) was assessed using a GFP-tagged human RPA2 cDNA construct that has been previously described^32,45^. The GFP-RPAp34 construct was transfected into RPE cells using FuGENE 6 according to the manufacturer’s protocol. Treatment with 1 μg/mL aphidicolin or 0.5 μM etoposide was initiated at 48 h after transfection. Alternatively, transduction with two independent *STAG2* lentiviral shRNAs was performed at 24 h after transfection. Cells were pulsed with 5-bromo-2′-deoxyuridine (BrdU) for 30 min prior to fixation with 10% neutral-buffered formalin. BrdU pulse and fixation was at 16 h after aphidicolin or etoposide treatment and 48 h after lentiviral shRNA transduction. Cells were permeabilized with 0.2% Triton X-100, treated with 20 U/mL DNase I in PBS, and then incubated with anti-BrdU primary antibody overnight at 4 ^o^C. Cover slips were washed, incubated with fluorescent-conjugated secondary antibodies (Molecular Probes) for 2 h at room temperature, followed by counterstaining and mounting on glass slides with Vectashield Mounting Medium containing DAPI (Vector Labs).

Beta-galactosidase activity was assessed on RPE, BJ, and SVG p12 cells at 14 days following lentiviral transduction with empty pLKO.1 or two independent *STAG2* shRNAs. Cells were washed with PBS, fixed in 1× Fixative Solution, and incubated with the freshly prepared β-Galactosidase Staining Solution overnight at 37 °C according to manufacturer’s protocol (Senescence b-Galactosidase Staining Kit, Cell Signaling).

Fluorescent imaging was performed on a Leica TCS SP8 laser scanning confocal microscope (DMi8 platform) using Leica Application Suite X software and Leica PCO Edge 5.5 sCMOS camera. Imaging was performed using either 100×/1.44 oil-immersion HC Plan Apo objective lens or 60×/1.40 oil-immersion HC Plan Apo CS2 objective lens. Excitation used Leica Express UI white light lasers, and multidimensional image acquisition was performed using a combination of PMT and HyD detectors for multiple fluorescent dyes. Sequential scans were performed between frames to minimize fluorescence bleed through among multiple fluorophores. A pinhole value of 1.0 was used for 53BP1 imaging, while a pinhole of 3.0 was used for single fiber DNA replication fork imaging. Images were acquired in 1024 × 1024 pixel format, and 3–8 line averages or frame averages were recorded at moderate speed (100–200). For quantification of 53BP1 foci, approximately 2,000 cells were evaluated from two independent experiments for each condition. For quantification of cells with GFP-RPAp34 foci, approximately 600 cells were evaluated from three independent experiments for each condition.

Phase contrast imaging was performed on an Olympus CKX53 inverted microscope equipped with an Olympus DP27 CCD camera using Olympus LCAch N objectives and Olympus cellSens imaging software.

### Single fiber DNA replication fork progression analysis

Analysis of single fiber DNA replication fork progression was performed in hTERT-immortalized human RPE cells. See experimental schematic in Fig. 2a. Lentiviral transduction with empty pLKO.1 or two independent *STAG2* shRNAs was performed on exponentially growing cells. At 48 h following infection, culture media was supplemented with the DNA nucleotide analog 5-iodo-2′-deoxyuridine (IdU) to a final concentration of 25 µM. Following pulse with IdU at 37 °C for 30 min, cells were washed with media. Cells were then incubated in cell culture media supplemented with the DNA nucleotide analog 5-chloro-2′-deoxyuridine (CldU) to a final concentration of 250 µM at 37 °C for another 30 min. Following this second pulse with CldU, cells were trypsinized and washed with chilled PBS. Cells were then counted and re-suspended at a concentration of 5 × 10^5^ cells/mL in chilled PBS. Two microliter of the cell suspension was spotted at the end of glass slides. Cells were allowed to air dry for approximately 5 min to reduce the volume significantly, but not to completely dry. Subsequently, 7 µL of the cell lysis solution (200 mM Tris-HCl, pH 7.5, 50 mM EDTA, and 0.5% SDS) was applied on top of the cell suspension, mixed by gently stirring with a pipette tip, and allowed to lyse for 2 min. Slides were then tilted to approximately 15° to allow the DNA fibers to slowly flow across the slide and subsequently allowed to dry. Slides were then fixed with a solution of methanol and acetic acid (3:1) for 10 min at room temperature. Subsequently, slides were washed in distilled water and immersed in 2.5 M hydrochloric acid for 90 min. After DNA denaturation, slides were washed three times with PBS for 5 min each. After removal of excess PBS, the area of the slide containing DNA fibers was circled with an ImmEdge pen (Vector Labs, H-4000). In a humidified chamber at room temperature, slides were covered with blocking solution (5% BSA in PBS) for 15 min followed by the primary antibody solution (mouse anti-BrdU clone B44 at 1:25 dilution and rat anti-BrdU clone BU1/75 (ICR1) at 1:400 dilution in blocking solution) for 3 h. Subsequently, slides were washed three times with PBS for 5 min each, followed by incubation with secondary antibody solution (sheep anti-mouse Cy3-conjugated at 1:500 dilution and goat anti-rat Alexa Fluor 488-conjugated at 1:400 dilution in blocking solution) for 90 min at room temperature. Finally, slides were washed three times with PBS, and allowed to air dry before mounting with Vectashield Mounting Medium. Slides were sealed with coverslip sealant. In order to validate this single fiber DNA replication fork assay, this analysis was also performed on un-infected, exponentially growing RPE cells after introduction of the DNA polymerase inhibitor aphidicolin at 10 μg/mL at various timepoints before and during the IdU and CldU nucleotide pulses. See experimental schematic in Supplementary Fig. 2a.

Imaging of the single fiber DNA replication forks was performed using a Leica TCS SP8 laser scanning confocal microscope and Leica Application Suite X software as described in the microscopy section above. Imaging and assessment was performed in regions where fibers were well separated and not entangled. Greater than 200 individual replication forks were evaluated from more than 20 fields from two independent experiments per condition. Evaluation and quantification was performed in a blinded fashion to prevent bias. A summary of the replication fork categorization is shown in Fig. S2C and is described here. Fibers with equal IdU and CldU labeling were categorized as unidirectional elongating fork (normal fork progression). Fibers with CldU labeling at both ends of IdU labeling were categorized as bi-directional replication (normal fork progression). Fibers with ldU labeling at both ends of CIdU labeling were categorized as completion of replication (normal fork progression). Fibers with CldU labeling only were categorized as new origin of replication during second pulse (indeterminate fork progression, excluded from quantitation shown in Fig. 2c). In contrast, abnormal fork progression was scored for fibers with absence of CldU labeling (stalled fork during first pulse), fibers with unequal IdU and CldU labeling (either stalled fork during second pulse or stalled fork during first pulse with re-initiation), or fibers with both minimal IdU and CldU labeling (stalled fork throughout both pulses). Quantitation of normal fork progression versus abnormal fork progression is shown in Fig. 2c following lentiviral transduction with empty pLKO.1 or two independent *STAG2* shRNAs, while detailed quantitation of the individual categories of replication fork progression are shown in Supplementary Fig. 2d.

### Cell cycle-dependent 53BP1 foci analysis

U87MG human glioblastoma cells were synchronized in G1 phase of the cell cycle by culturing in the presence of 1 µM palbociclib for 48 h. Following synchronization, lentiviral transduction with empty pLKO.1 or *STAG2* shRNA was performed, and selection with 2 μg/mL puromycin was initiated at 24 h after infection. Following completion of puromycin selection, cells were released from palbociclib arrest and fixed for immunofluorescence at the indicated time points. See experimental schematic in Supplementary Fig. 4a. Depletion of STAG2 protein was confirmed by immunoblotting.

### Radiation, drug treatment, and shRNA synthetic lethality screen

The CellTiter-Glo assay (Promega) was used to assess cell survival following radiation, drug treatment, or lentiviral shRNA transduction of four pairs of *STAG2* isogenic human cell lines. 500 exponentially growing cells were seeded per well in clear-bottom white polystyrene 96-well microplates (Corning 3903). At 24 h after seeding, cells were irradiated, treated with various drugs, or lentiviral shRNAs as indicated. At 4 days following treatment, an equal volume of CellTiter-Glo One Solution reagent was added to each of the wells, followed by shaking for five min at 120 rpm at room temperature. Luminescence was measured using the SpectraMax M5 spectrophotometer (Molecular Devices). For gamma irradiation, cultured cells were exposed to radioactive cesium isotope (Cs-137) at the indicated dosage. Ultraviolet irradiation was delivered using the UV Stratalinker-2400 (Stratagene) at the indicated dosage. All drugs used for the cell survival assays were obtained from Selleck Chemicals or Tocris Bioscience. All drugs were dissolved in DMSO, except for cisplatin that was dissolved in *N,N*-dimethylformamide. Drugs were used at the indicated concentrations. Five independent lentiviral shRNAs for each of the 10 DNA repair genes included in the shRNA synthetic lethality screen were first tested on the U87MG human glioblastoma cell line to determine the two shRNAs that produced the most efficient depletion as assessed by Western blot. Two independent shRNAs were then used to conduct the synthetic lethality screen for each of the 10 DNA repair genes in the four *STAG2* isogenic human cell lines. For the gamma and ultraviolet irradiation experiments, 12 replicates from 3 independent experiments were performed for each experimental condition. For the drug screen, 12 replicates from 3 independent experiments were performed for each experimental condition. For the shRNA synthetic lethality screen, 8 replicates from 2 independent experiments were performed for each experimental condition. Percent survival was assessed as the mean luminescence of the experimental condition relative to the mean luminescence of the control cells (either no radiation, DMSO vehicle, or empty pLKO.1).

### Clonogenic survival assay

Four pairs of isogenic *STAG2* human cell lines (H4, 42MGBA, TC-106, and RPE-sh*TP53*) were used. Five hundred cells were seeded per well in 6-well plates. After 24 h, DMSO vehicle or cyclophosphamide at the indicated dosages was added to the culture media. Cultures were maintained for two weeks to allow colony formation, with repletion of media supplemented with fresh DMSO or cyclophosphamide every two days. Plates were stained with 0.5% crystal violet in 25% methanol solution. Colonies were imaged and counted using the GelCount instrument (Oxford Optronix). Three replicates were performed for each experimental condition.

### Quantification and statistical analysis

Statistical analysis was performed using GraphPad Prism or Microsoft Excel. All error bars shown represent standard error of the mean. All statistical details of experiments are included in the Figure legends or specific Methods section.

### Reporting summary

Further information on experimental design is available in the Nature Research Reporting Summary linked to this article.

## Supplementary information

Supplementary Information

Peer Review File

Reporting Summary

Source Data

## Data Availability

The source data underlying Figs. 1–8 and Supplementary Figures 1-8 are provided as a Source Data file. A reporting summary for this Article is available as a Supplementary Information file. All data is available from the authors upon reasonable request.
