# Supplementary material for: A requirement for STAG2 in replication fork progression creates a targetable synthetic lethality in cohesin-mutant cancers
Source: Nat Commun. 2019 Apr 11;10:1686. doi: 10.1038/s41467-019-09659-z (PMC6459917; doi:10.1038/s41467-019-09659-z)
Supplement: Supplementary file 1 — Supplementary Information [file 41467_2019_9659_MOESM1_ESM.pdf]

Supplementary Information for:

Mondal et al.

A requirement for STAG2 in replication fork progression creates a targetable synthetic lethality with DNA repair factors in cohesin-mutant cancers

Nature Communications

Contents:

Supplementary Figures 1-8

Supplementary Tables 1-5

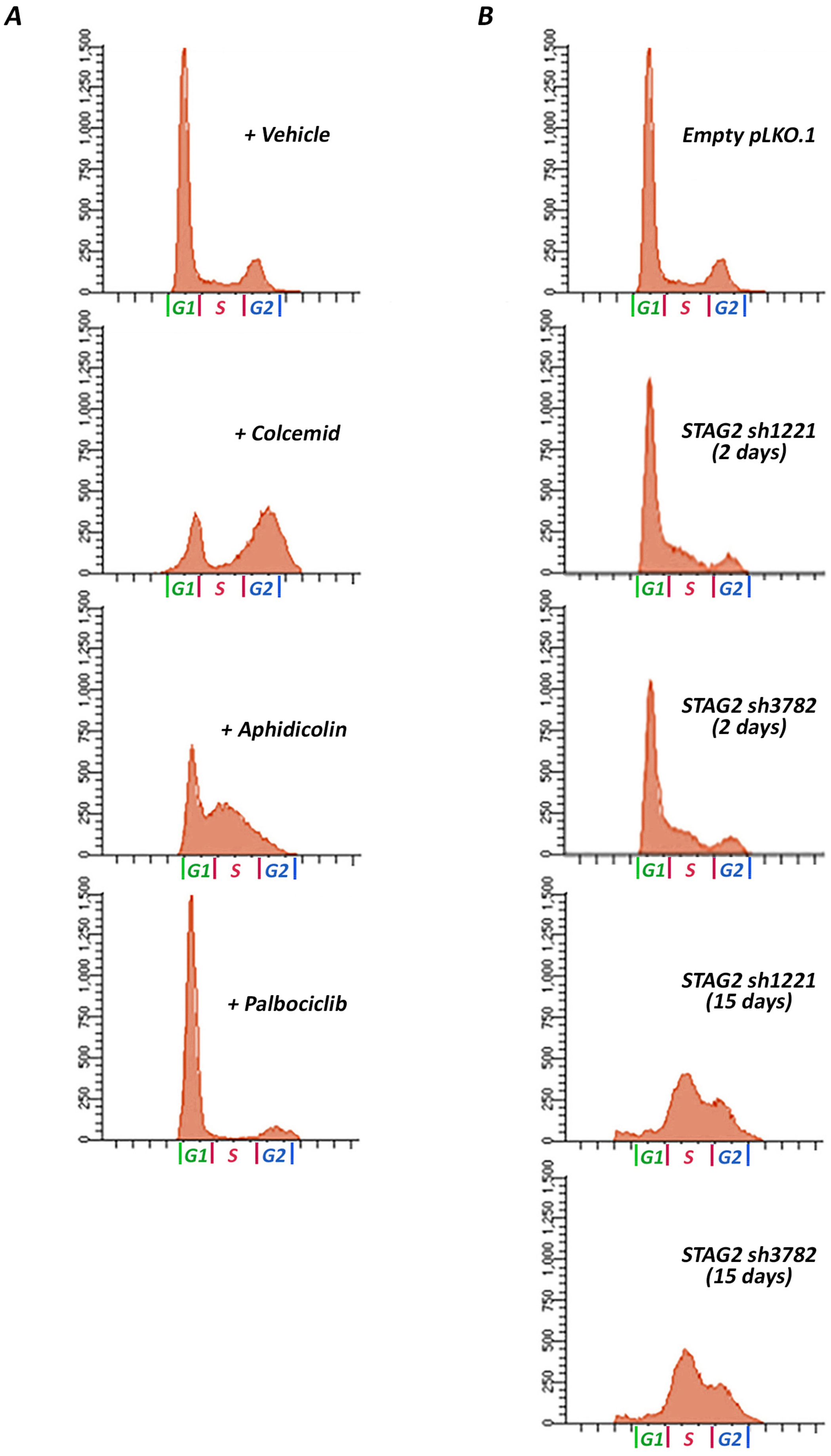

Supplementary Figure 1

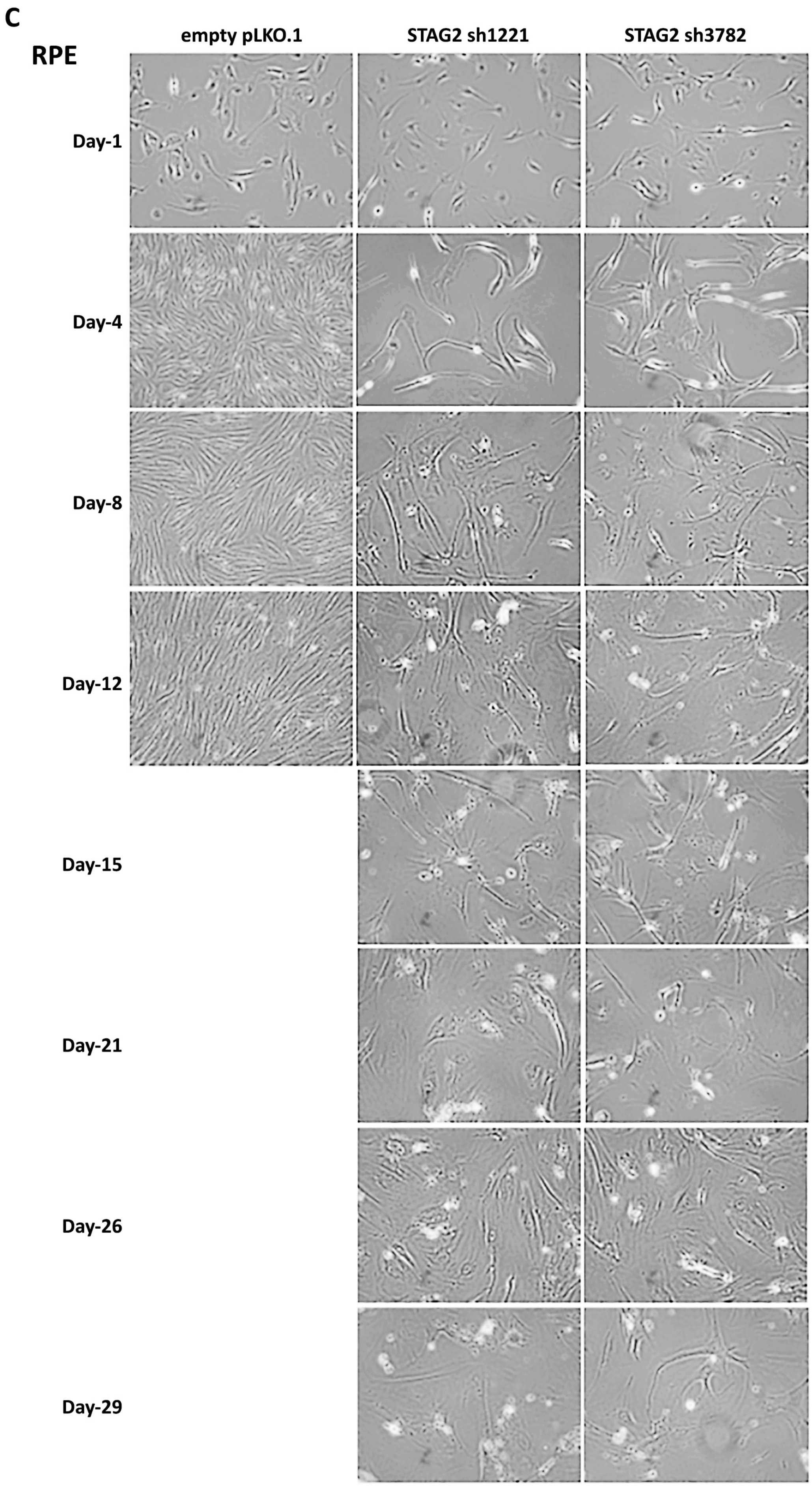
C
RPE
empty pLKO.1
STAG2 sh1221
STAG2 sh3782
Day-1
Day-4
Day-8
Day-12
Day-15
Day-21
Day-26
Day-29

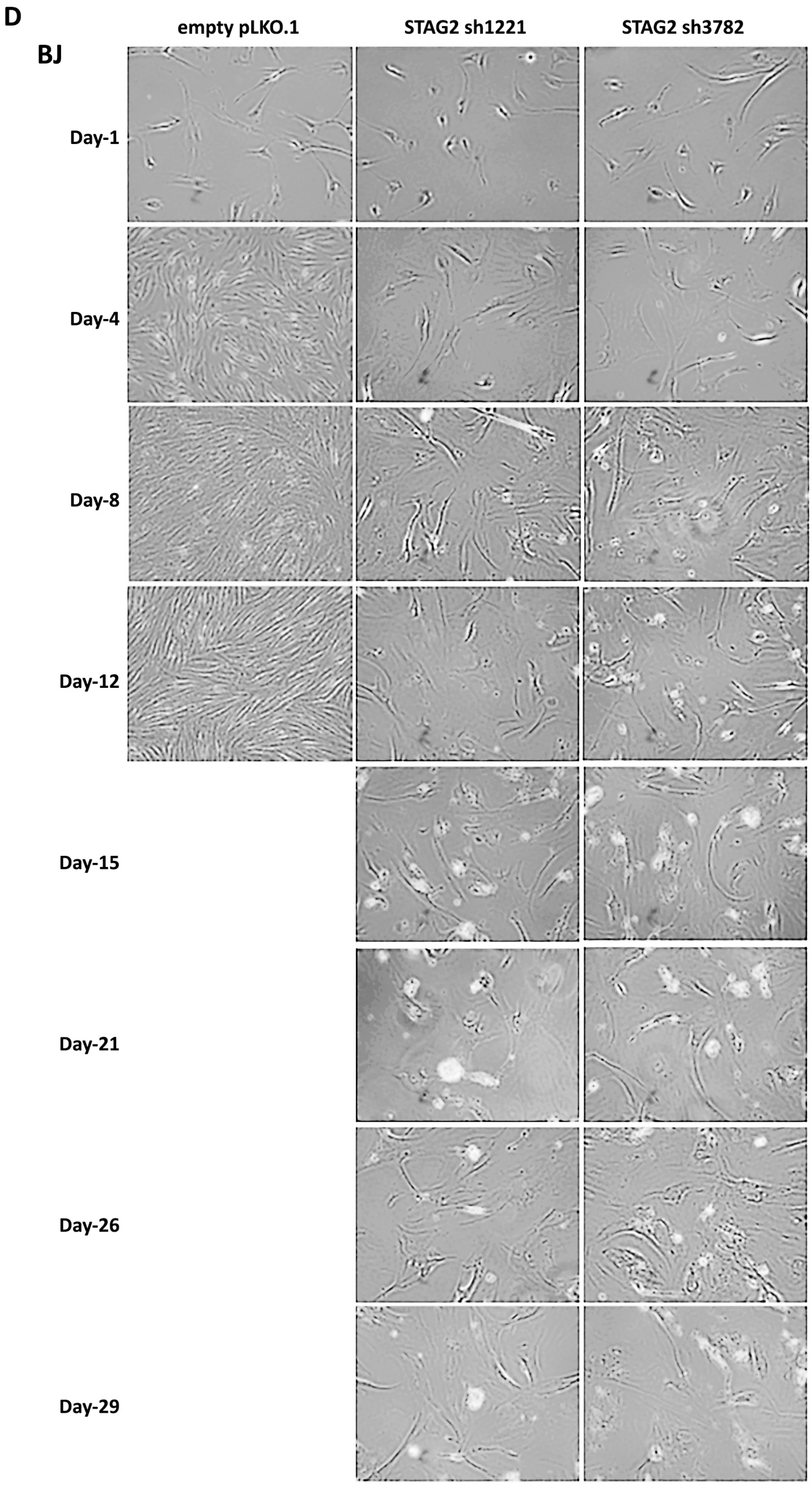

Supplementary Figure 1

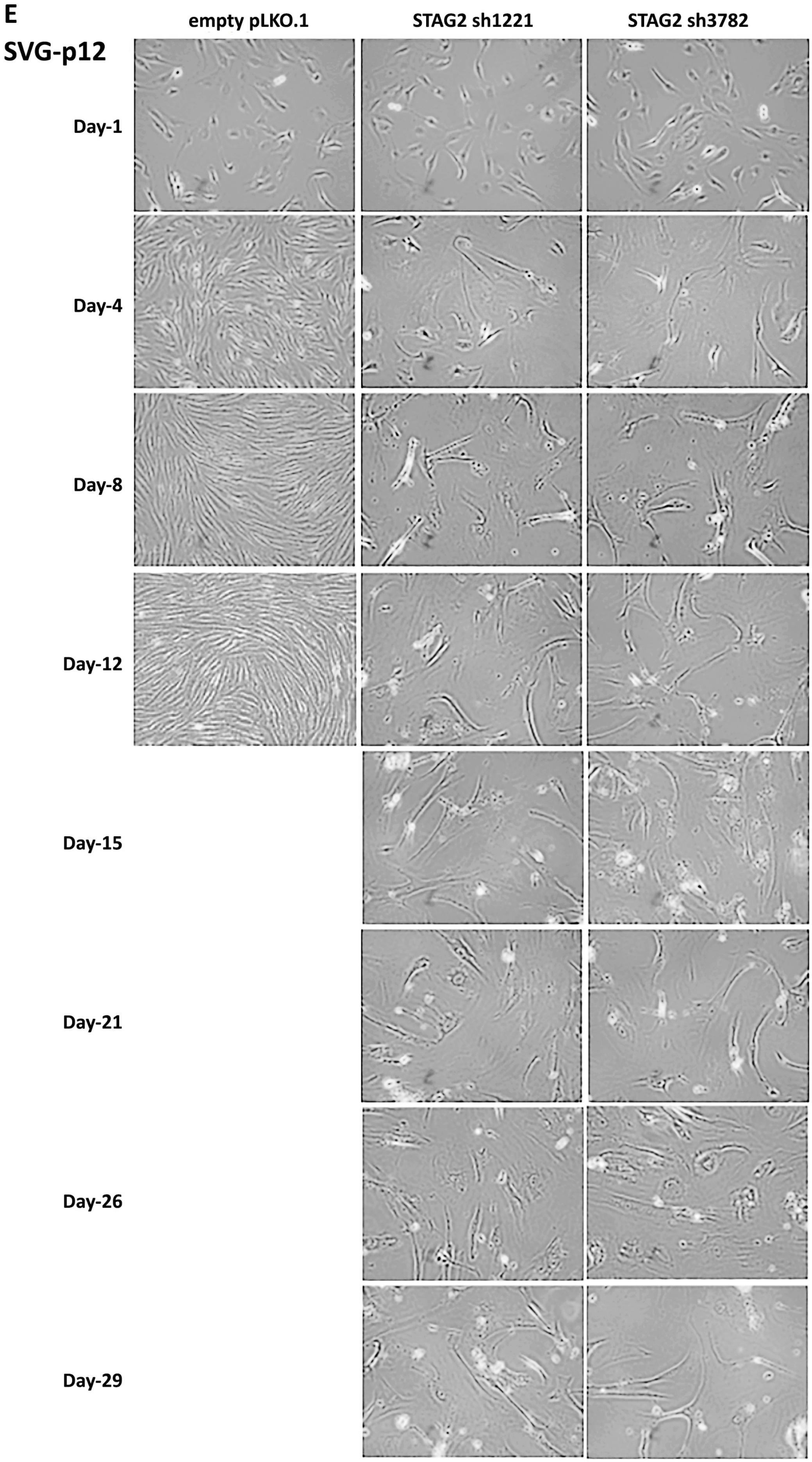
E
SVG-p12
empty pLKO.1
STAG2 sh1221
STAG2 sh3782
Day-1
Day-4
Day-8
Day-12
Day-15
Day-21
Day-26
Day-29

F

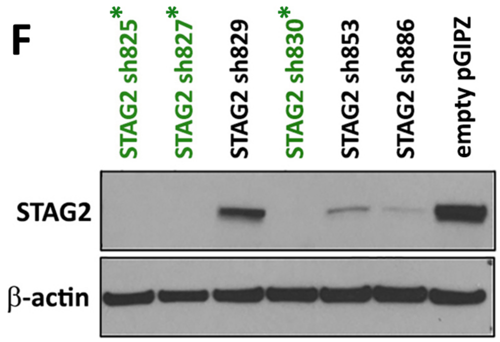

G

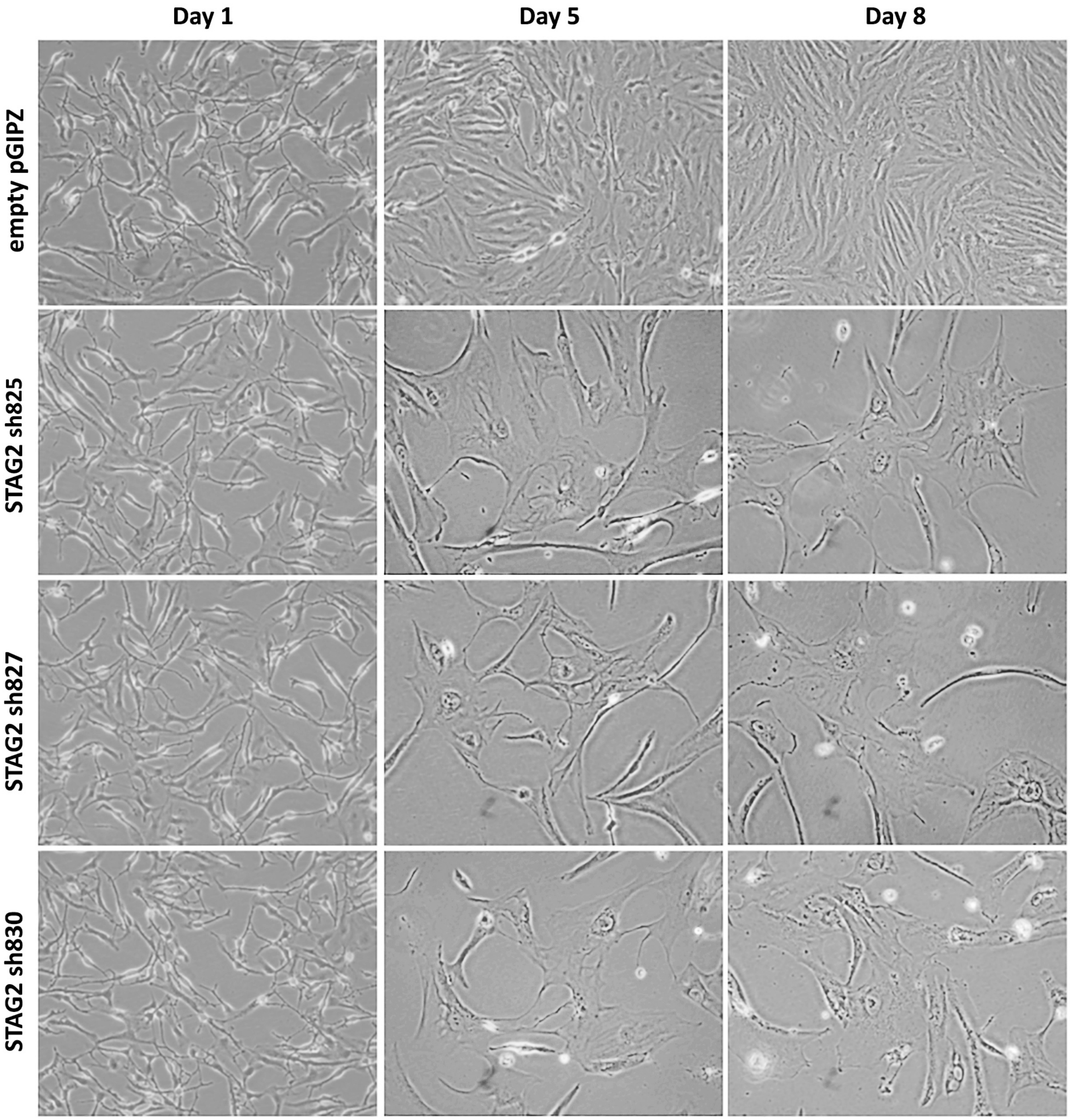

Supplementary Figure 1

H

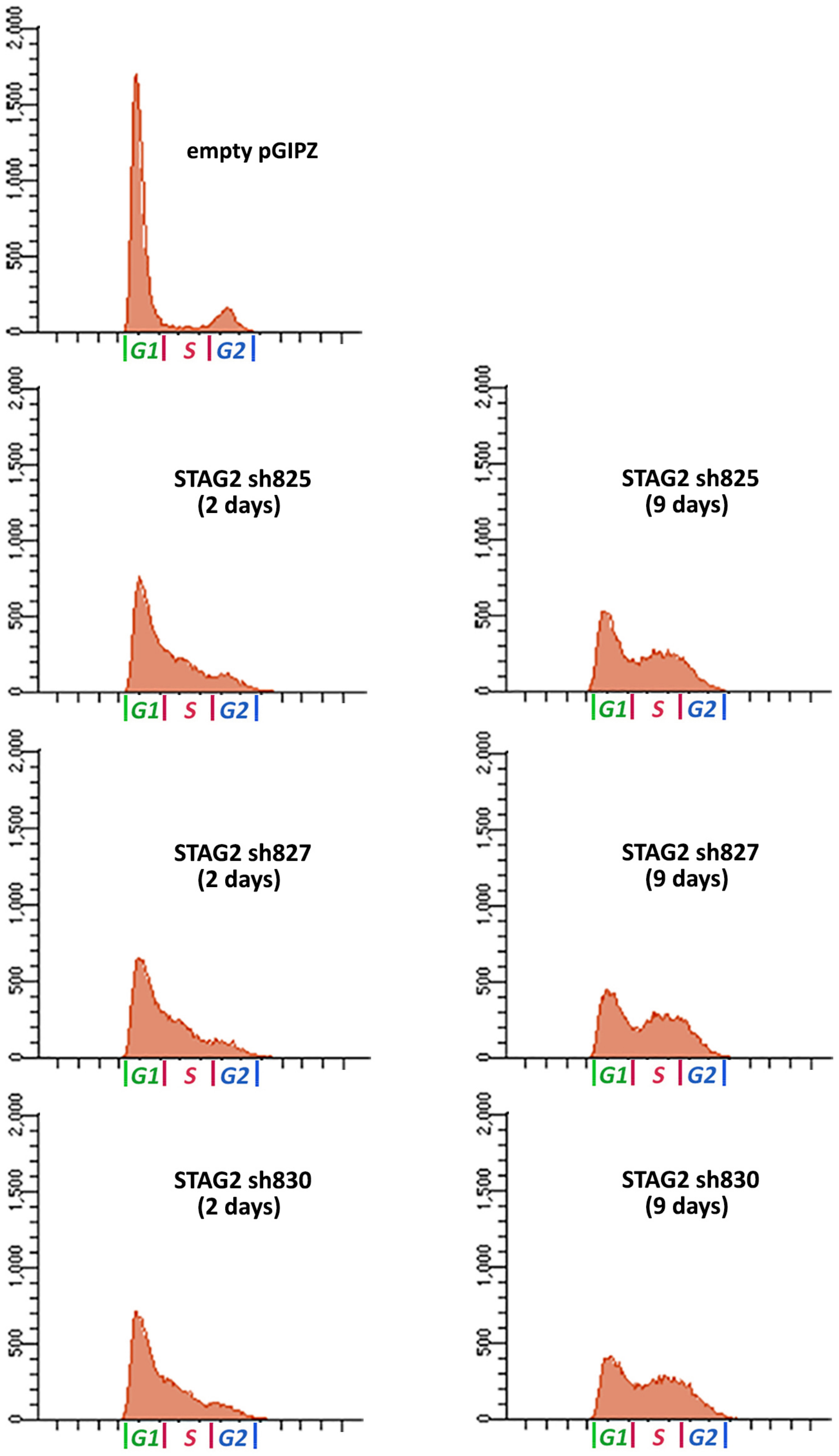
empty pGIPZ
STAG2 sh825 (2 days)
STAG2 sh825 (9 days)
STAG2 sh827 (2 days)
STAG2 sh827 (9 days)
STAG2 sh830 (2 days)
STAG2 sh830 (9 days)
G1
S
G2
0
500
1,000
1,500
2,000

I

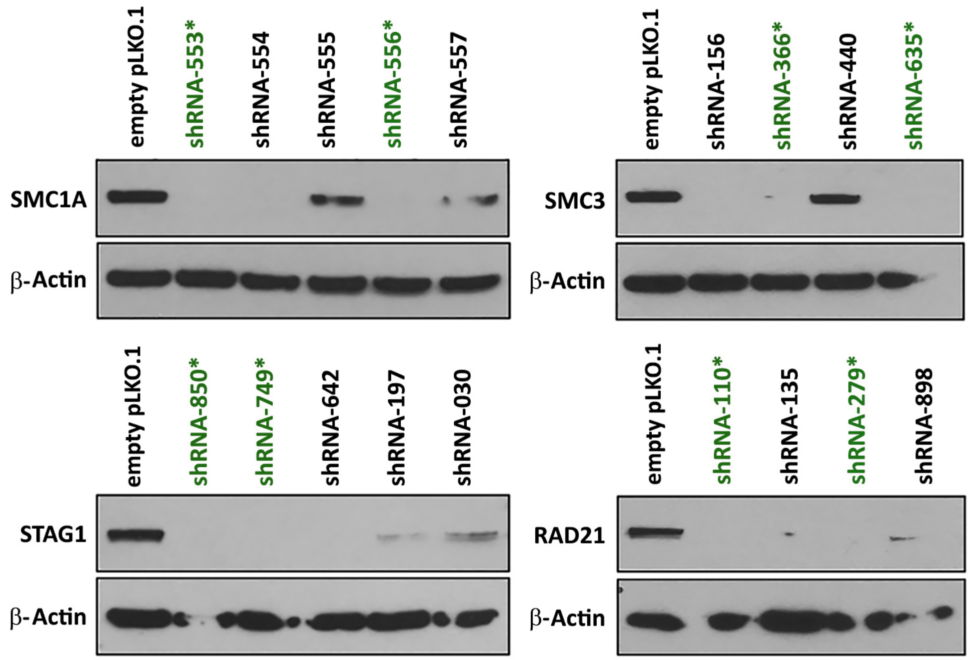
empty pLKO.1
shRNA-553*
shRNA-554
shRNA-555
shRNA-556*
shRNA-557
SMC1A
β-Actin
empty pLKO.1
shRNA-156
shRNA-366*
shRNA-440
shRNA-635*
SMC3
β-Actin
empty pLKO.1
shRNA-850*
shRNA-749*
shRNA-642
shRNA-197
shRNA-030
STAG1
β-Actin
empty pLKO.1
shRNA-110*
shRNA-135
shRNA-279*
shRNA-898
RAD21
β-Actin

J

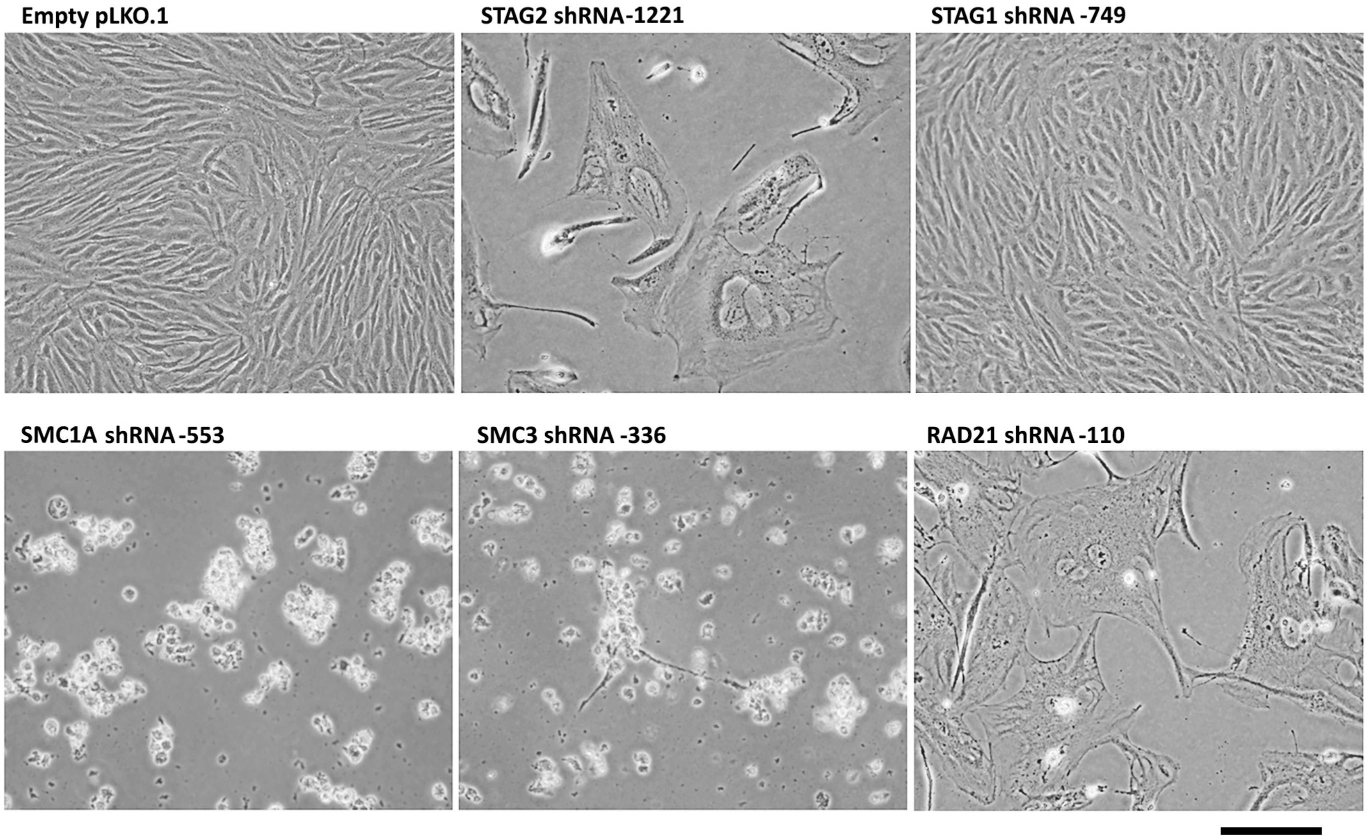
Empty pLKO.1
STAG2 shRNA-1221
STAG1 shRNA -749
SMC1A shRNA -553
SMC3 shRNA -336
RAD21 shRNA -110

Supplementary Figure 1. *STAG2* depletion in primary human cells results in intra-S-phase cell cycle arrest, whereas depletion of the core cohesin subunits *SMC1A*, *SMC3*, and *RAD21* results in cell death. **A,** Flow cytometry plots of RPE cells after treatment with DMSO vehicle, a microtubule polymerization inhibitor (colcemid), a DNA polymerase inhibitor (aphidicolin), and a cyclin-dependent kinase 4/6 inhibitor (palbociclib). Y-axis, cell count. X-axis, propidium iodide intensity. **B,** Flow cytometry plots of RPE cells after lentiviral transduction with empty pLKO.1 and two independent *STAG2* shRNAs, which demonstrate an abnormal accumulation of cells within S-phase after STAG2 depletion. **C-E,** Phase contrast image of RPE cells (**C**), BJ cells (**D**), and SVG p12 cells (**E**) following lentiviral transduction with empty pLKO.1 or *STAG2* shRNA demonstrating failure to proliferate and morphologic features of senescence. **F,** Validation of lentiviral shRNA mediated depletion of STAG2 using the pGIPZ vector system. Shown are immunoblots of total lysate from U87MG cells following lentiviral transduction with empty pGIPZ or multiple independent shRNA sequences against *STAG2*. The three shRNAs that produced the most effective protein depletion (highlighted in green) were used to assess cellular phenotypes in primary human cells. **G,** Phase contrast image of RPE cells following lentiviral transduction with empty pGIPZ or *STAG2* shRNA demonstrating failure to proliferate and morphologic features of senescence. **H,** Flow cytometry plots of RPE cells after lentiviral transduction with empty pGIPZ and three independent *STAG2* shRNAs, which demonstrate an abnormal accumulation of cells within S-phase after STAG2 depletion. **I,** Validation of lentiviral shRNA mediated depletion of cohesin ring components. Shown are immunoblots of total lysate from U87MG cells following lentiviral transduction with empty pLKO.1 or multiple independent shRNA sequences against the cohesin subunits *SMC1A*, *SMC3*, *STAG1*, and *RAD21*. The two shRNAs that produced the most effective protein depletion (highlighted in green) were used to assess cellular phenotypes in primary human cells. **J,** Phase contrast image of RPE cells at seven days following lentiviral transduction with empty pLKO.1 or shRNAs against cohesin components.

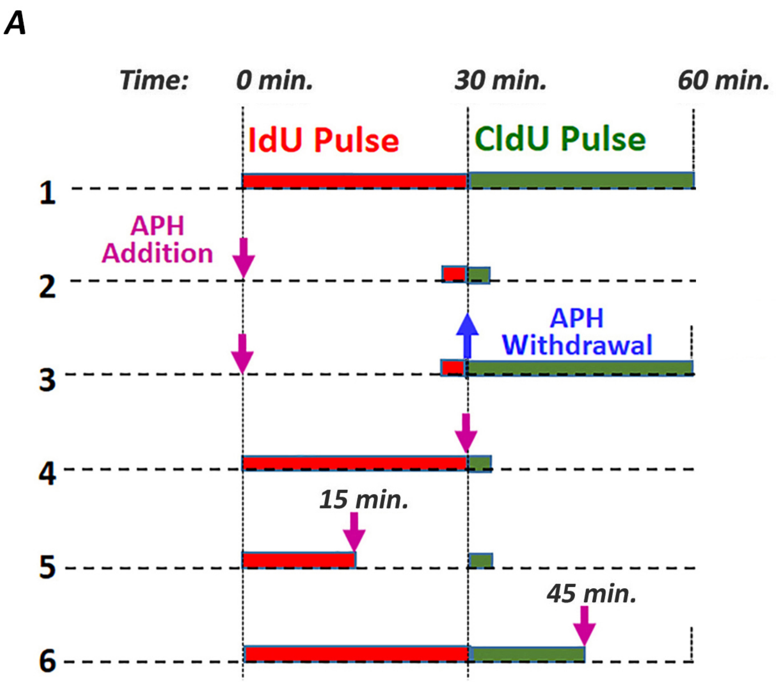

1) No block

2) APH Block throughout both (IdU and CldU) pulses

3) APH Block during first (Idu) pulse only

4) APH Block during second (CldU) pulse only

5) APH Block after first 15 minutes of (IdU) pulse

6) APH Block after first 15 minutes of (CldU) pulse

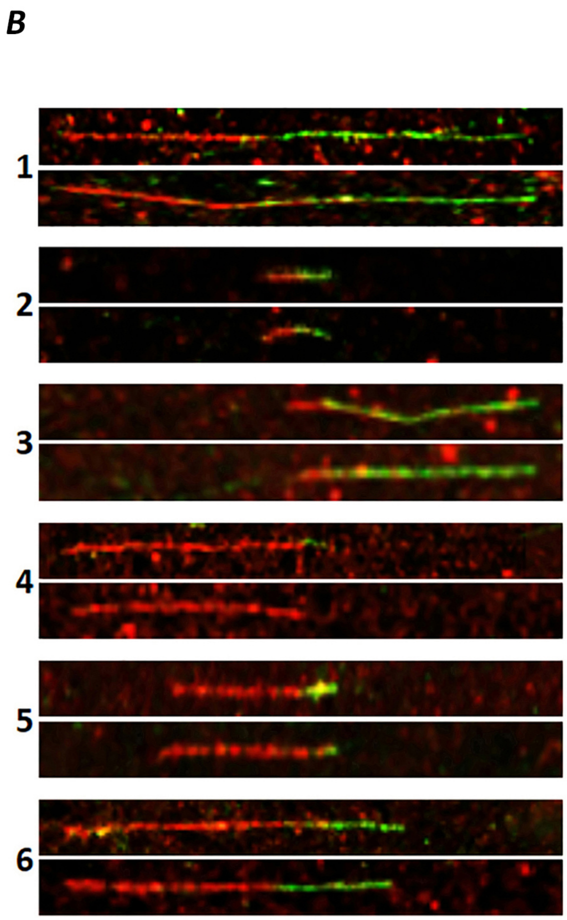

Supplementary Figure 2

C

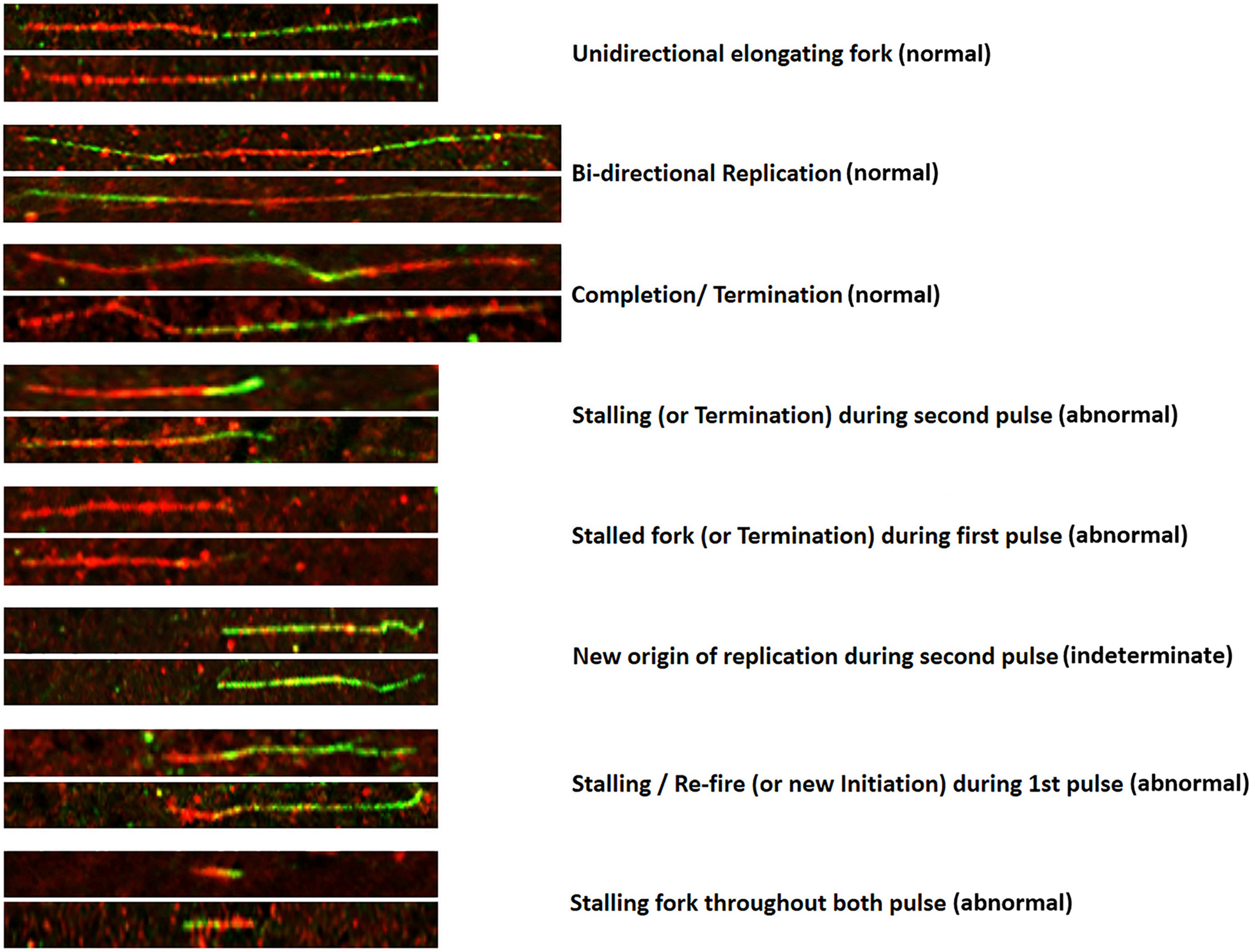

D

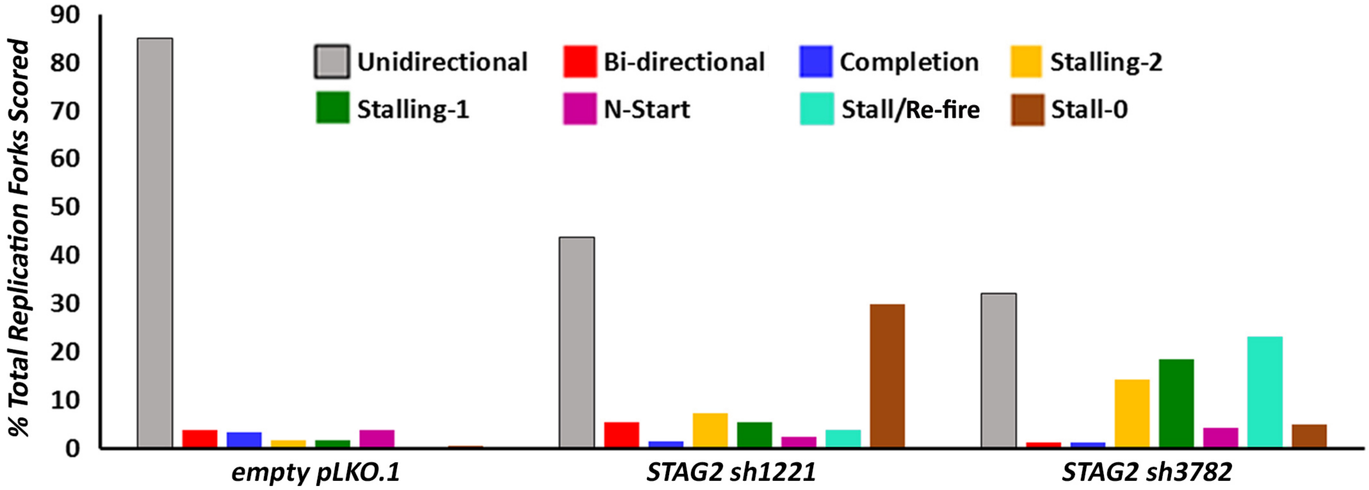

Supplementary Figure 2

E

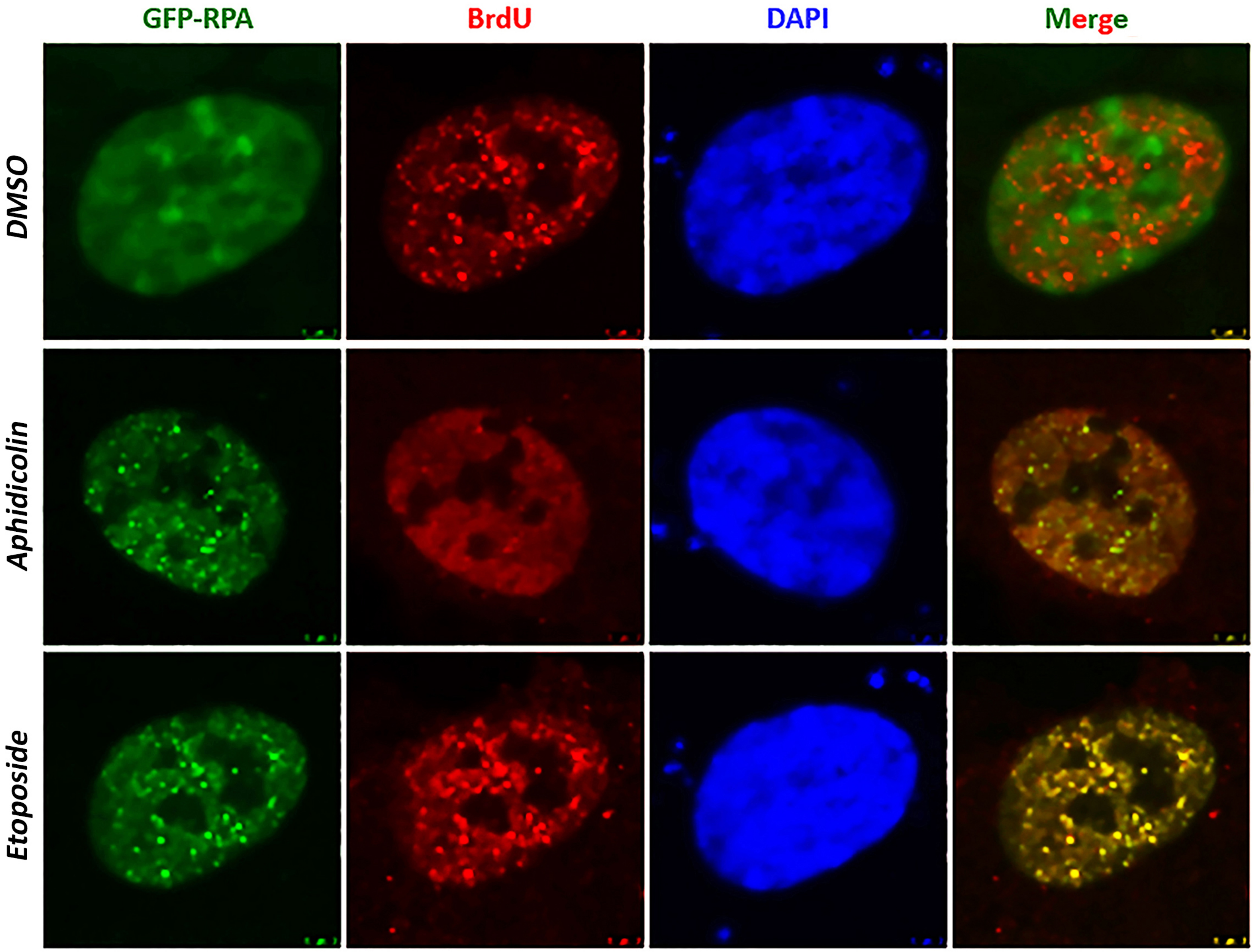

Supplementary Figure 2

F

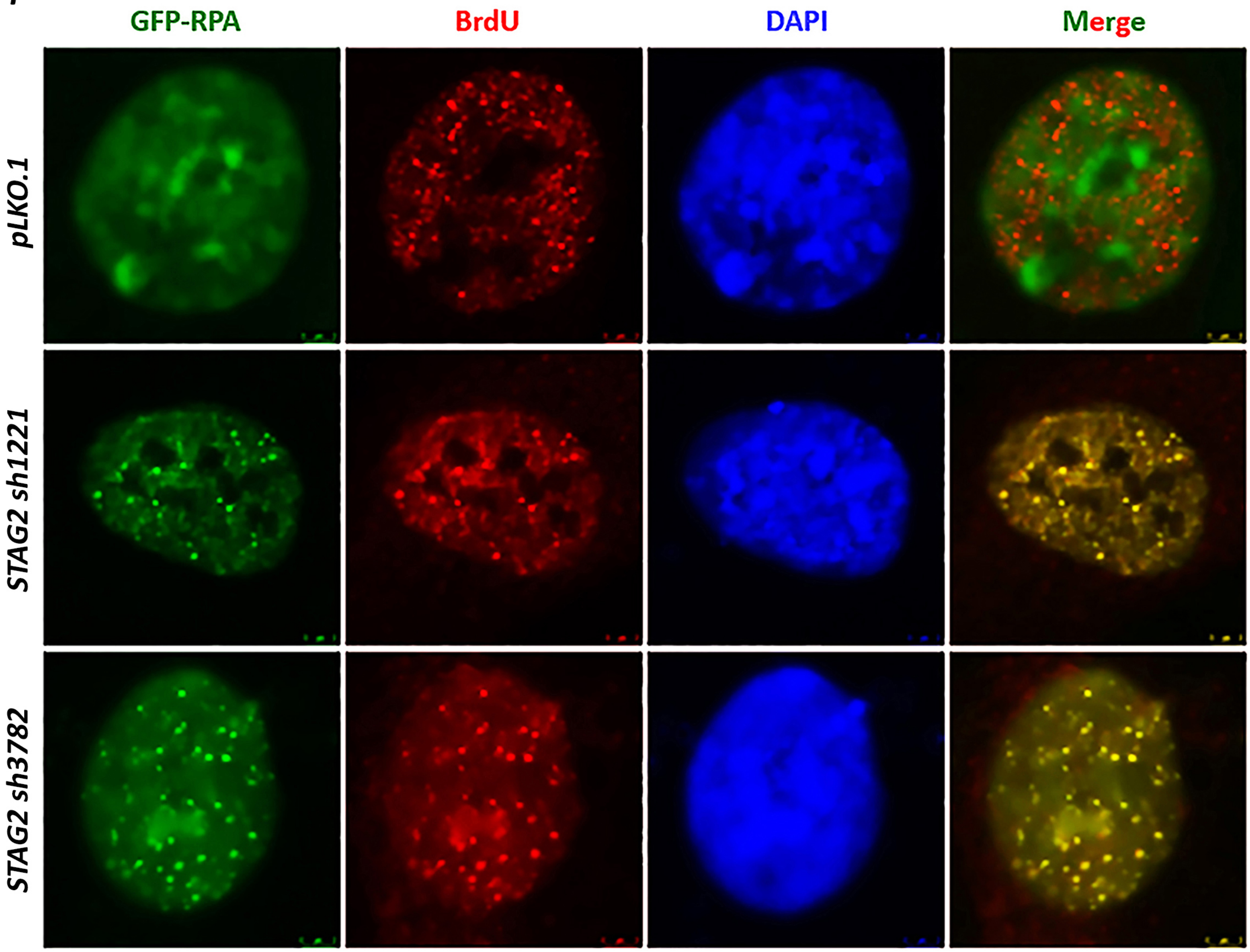

Supplementary Figure 2. Inactivation of *STAG2* in primary human cells causes replication fork stalling. **A,** Schematic diagram of DNA combing assay utilizing the DNA polymerase inhibitor aphidicolin (10 μg/mL) at various time points, which was performed to validate this methodology for assessment of replication fork progression. **B,** Microscopic images of representative single DNA fibers showing normal replication fork progression (1) and replication fork stalling at various timepoints induced by aphidicolin block (2-6). **C,** Microscopic images of representative single DNA fibers showing normal and various abnormal replication fork progression in RPE cells following lentiviral transduction with empty pLKO.1 or two independent *STAG2* shRNAs. **D,** Quantitation of normal and abnormal replication forks scored from DNA combing assay of RPE cells following lentiviral transduction with empty pLKO.1 or two independent *STAG2* shRNAs. More than 200 individual replication forks were evaluated from two independent experiments for each condition. **E,** Representative images of parental RPE cells showing localization of GFP-tagged RPAp34 during unchallenged DNA replication (DMSO vehicle), replication block (aphidicolin), and topoisomerase inhibition (etoposide). RPAp34 foci accumulation is only present upon DNA replication fork stalling. **F,** Representative images of RPE cells showing accumulation of GFP-tagged RPAp34 at stalled replication forks following lentiviral transduction with *STAG2* shRNA but not empty pLKO.1.

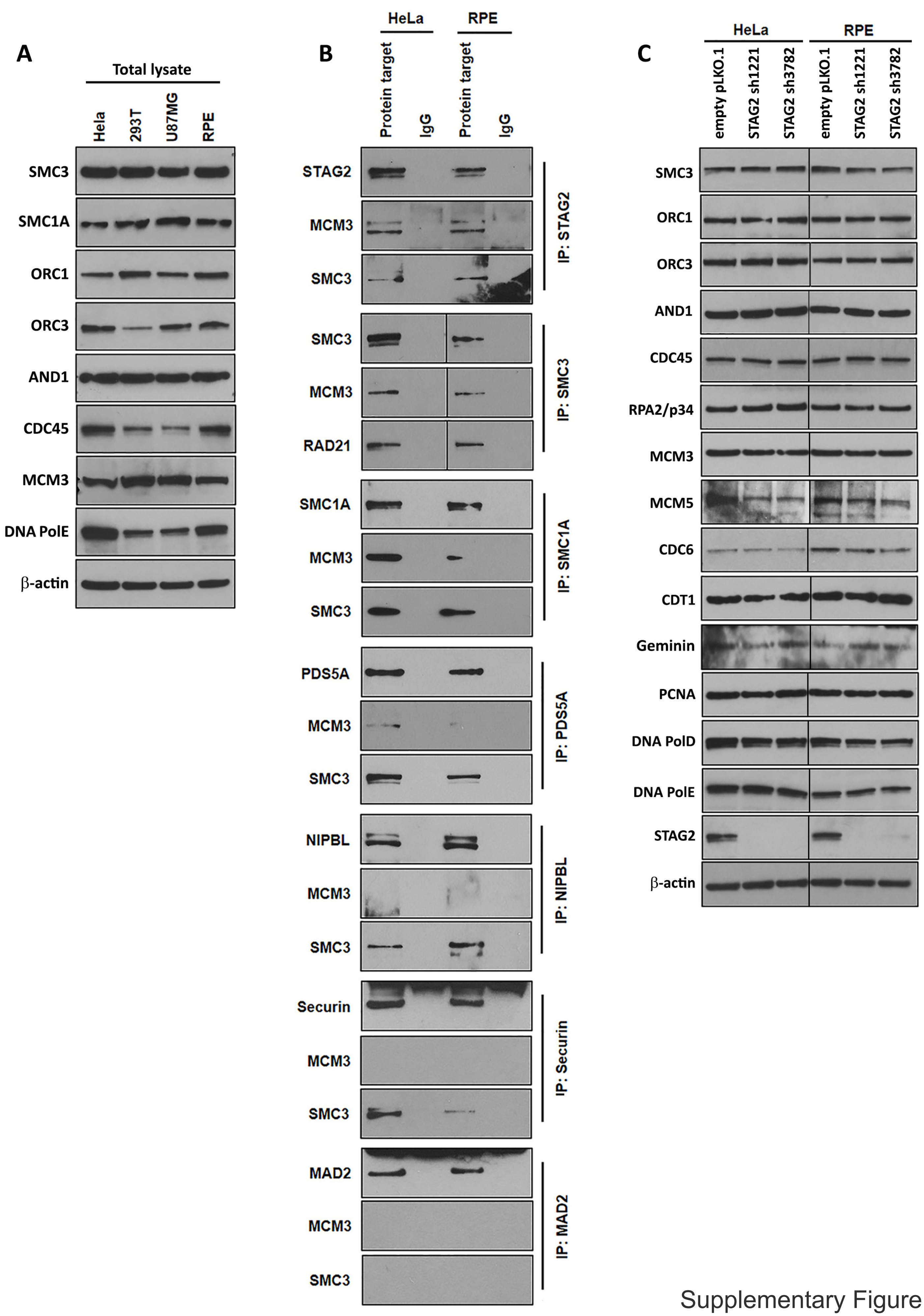

Supplementary Figure 3

Supplementary Figure 3. Inactivation of *STAG2* in primary human cells causes disruption of cohesin interaction with the replication machinery. **A,** Western blots of total cell lysate from HeLa, 293T, U87MG, and RPE cells for cohesin complex, origin recognition complex, pre-replication complex, and replication machinery components. **B,** Immunoprecipitation using antibodies against cohesin and cohesion regulatory factors in HeLa and RPE cells demonstrating a specific interaction of the MCM helicase subunit MCM3 with STAG2, SMC3, SMC1A, and PDS5A, but not the cohesin regulatory factors NIPBL and Securin. **C,** Western blots of total cell lysate from HeLa and RPE cells for origin recognition complex, pre-replication complex, and replication machinery components following depletion of STAG2 with two independent shRNAs.

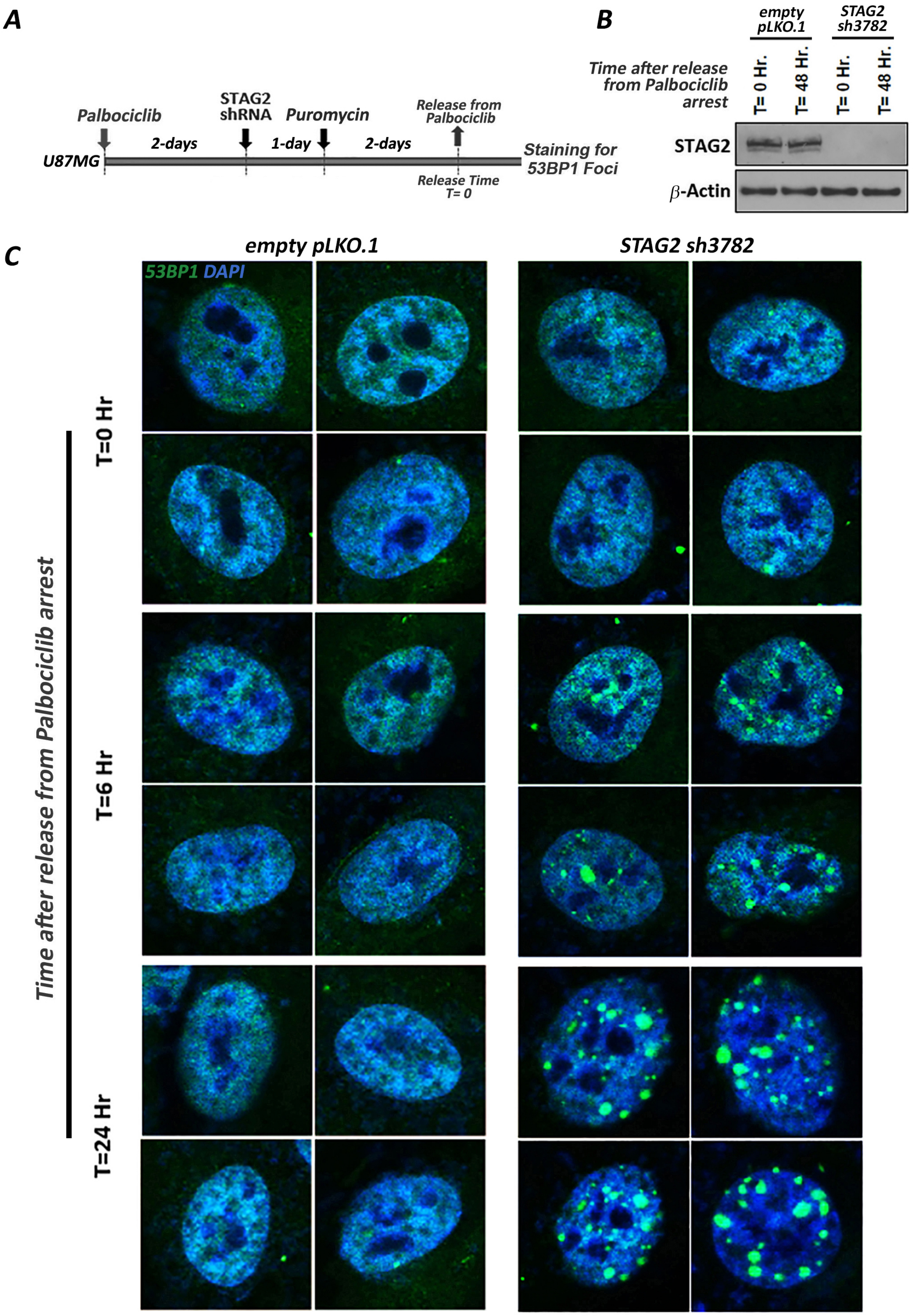

Supplementary Figure 4

Supplementary Figure 4. The replication fork stalling caused by *STAG2* depletion leads to fork collapse and DNA double-strand breaks. **A,** Schematic diagram of palbociclib arrest experiment to determine the requirement of cell cycle progression on the formation of 53BP1 foci following STAG2 depletion. **B,** Immunoblot demonstrating STAG2 depletion in RPE cells upon lentiviral shRNA transduction after palbociclib arrest. **C,** Representative immunofluorescence images of cells following sequential arrest in G1 using palbociclib, lentiviral transduction with empty pLKO.1 or *STAG2* shRNA, and then release from palbociclib arrest.

A

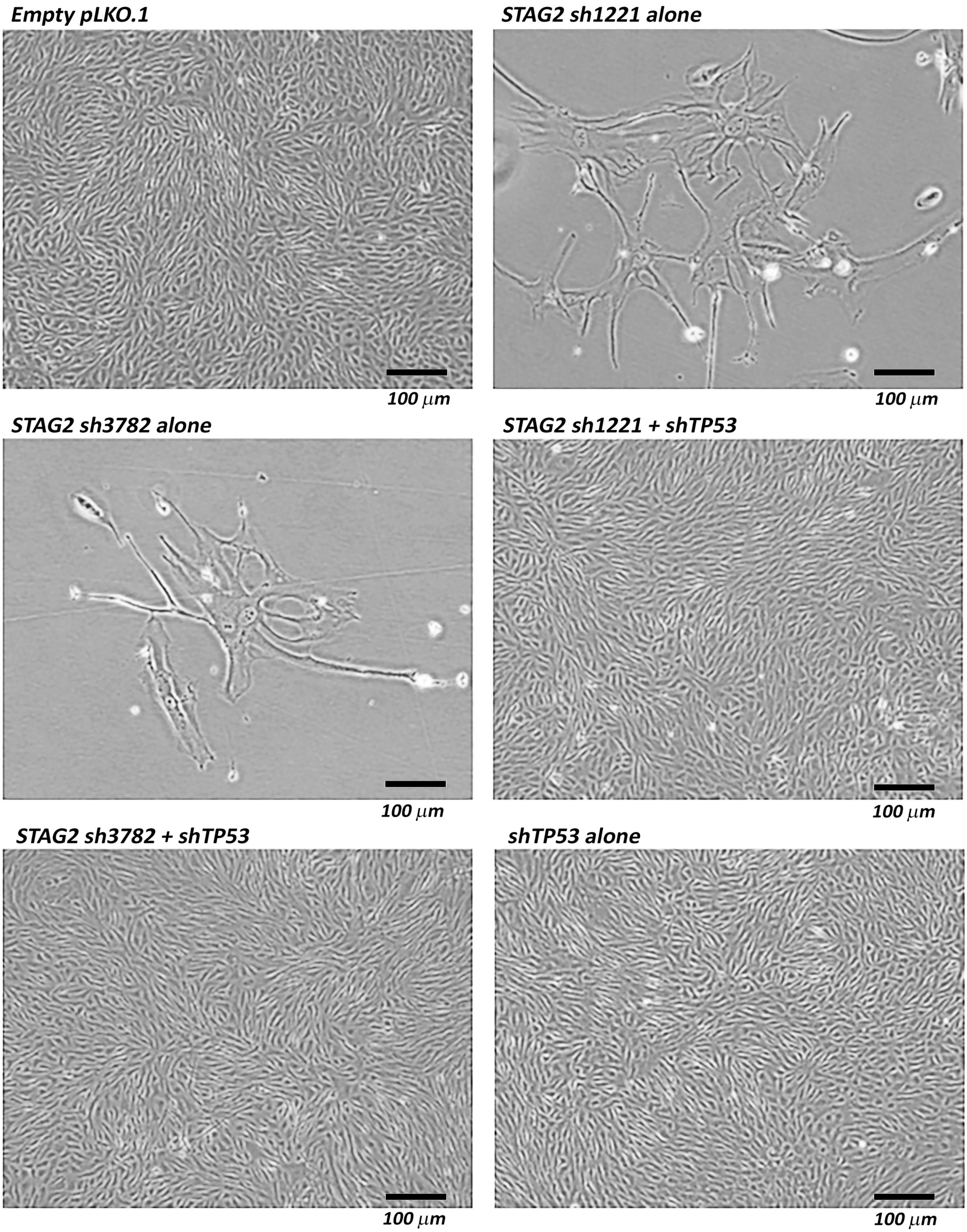

Supplementary Figure 5

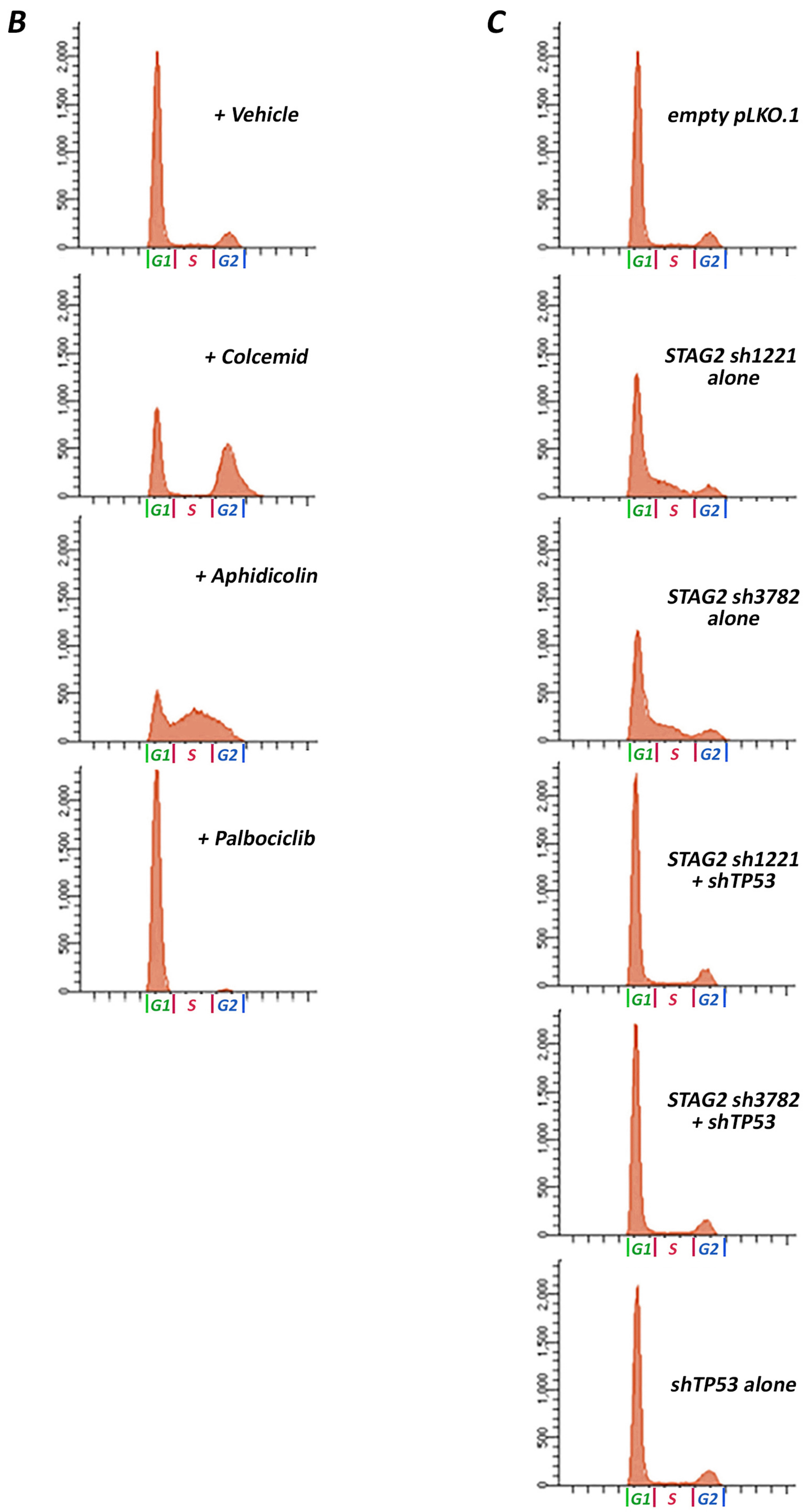

Supplementary Figure 5

Supplementary Figure 5. The intra-S-phase cell cycle arrest induced by *STAG2* inactivation is p53 dependent. **A,** Phase contrast image of RPE cells following lentiviral transduction with *STAG2* shRNA in combination with *TP53* shRNA demonstrating cellular proliferation and abrogation of the senescence induced by STAG2 depletion alone. **B,** Flow cytometry plot of parental RPE cells after treatment with DMSO vehicle, a microtubule polymerization inhibitor (colcemid), a DNA polymerase inhibitor (aphidicolin), and a cyclin-dependent kinase 4/6 inhibitor (palbociclib). Y-axis, cell count. X-axis, propidium iodide intensity. **C,** Flow cytometry plots of parental RPE cells following lentiviral transduction with *STAG2* shRNA in combination with *TP53* shRNA demonstrating bypass of the S-phase arrest induced by STAG2 depletion alone.

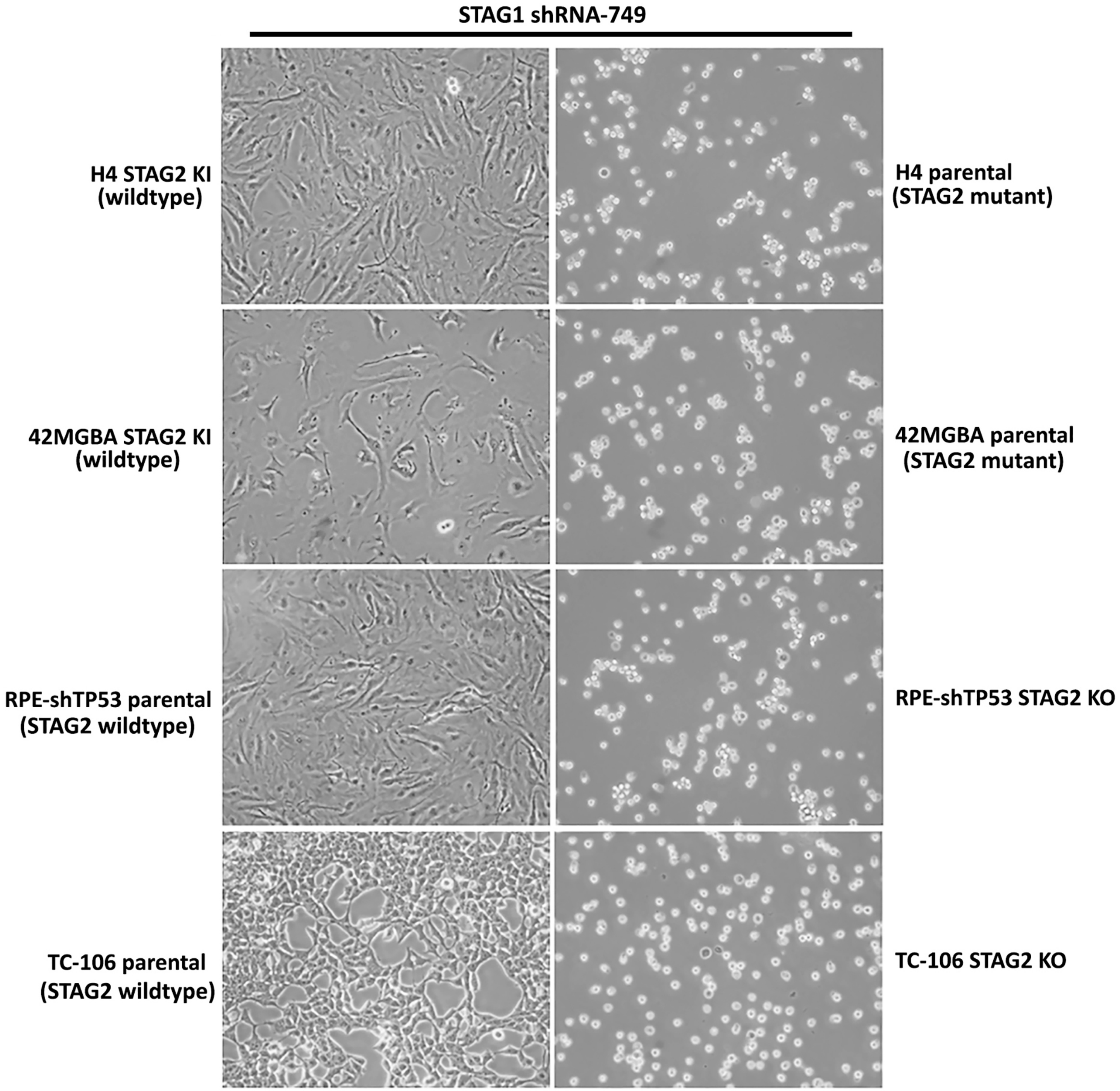

Supplementary Figure 6

Supplementary Figure 6. *STAG2* deficiency in human cancer cells creates a synthetic lethality with the cohesin subunit *STAG1*. Representative phase contrast images of *STAG2* isogenic H4, 42MGBA, TC-106, and RPE-sh*TP53* cells showing synthetic lethality of *STAG2* inactivation with shRNA depletion of *STAG1*.

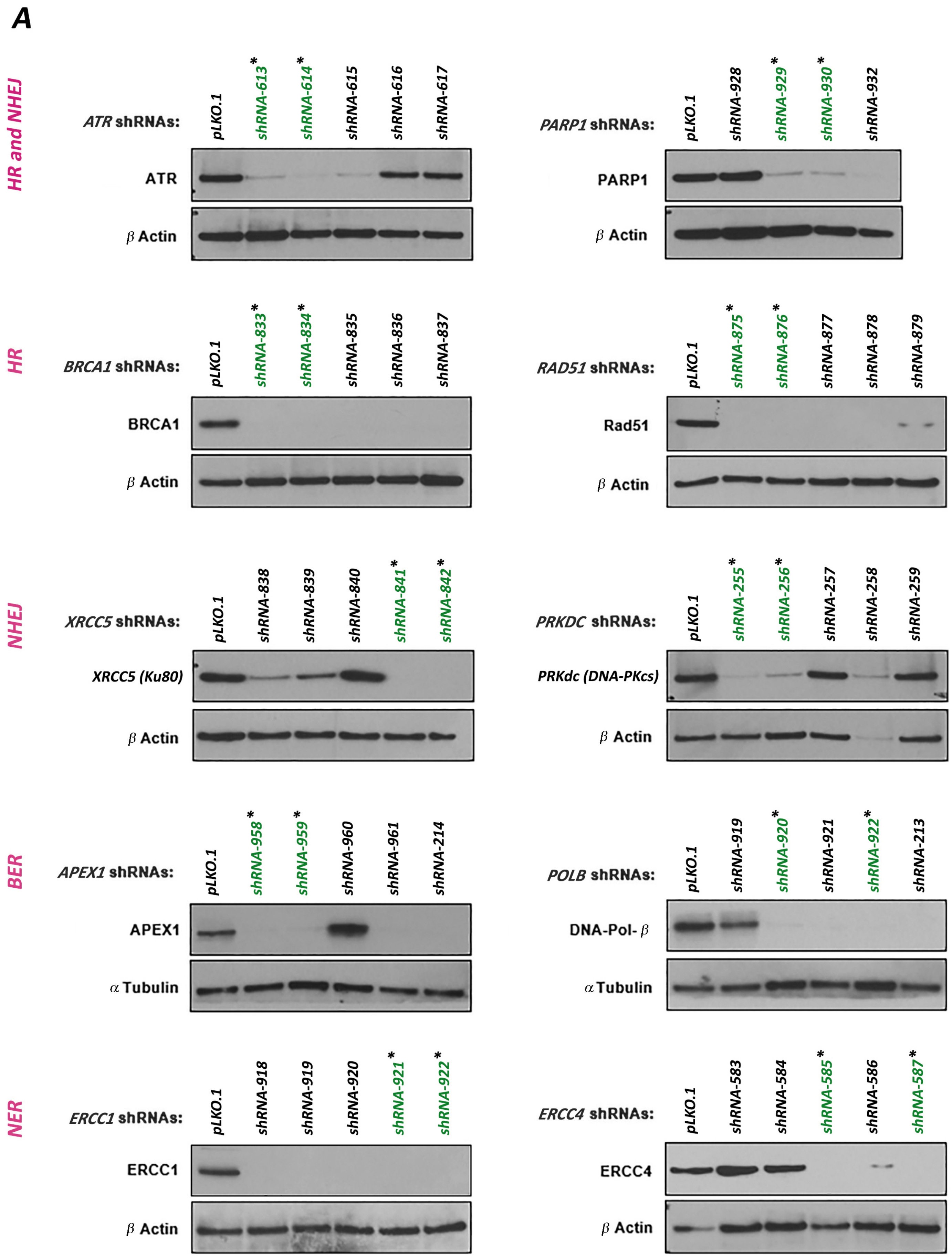

Supplementary Figure 7

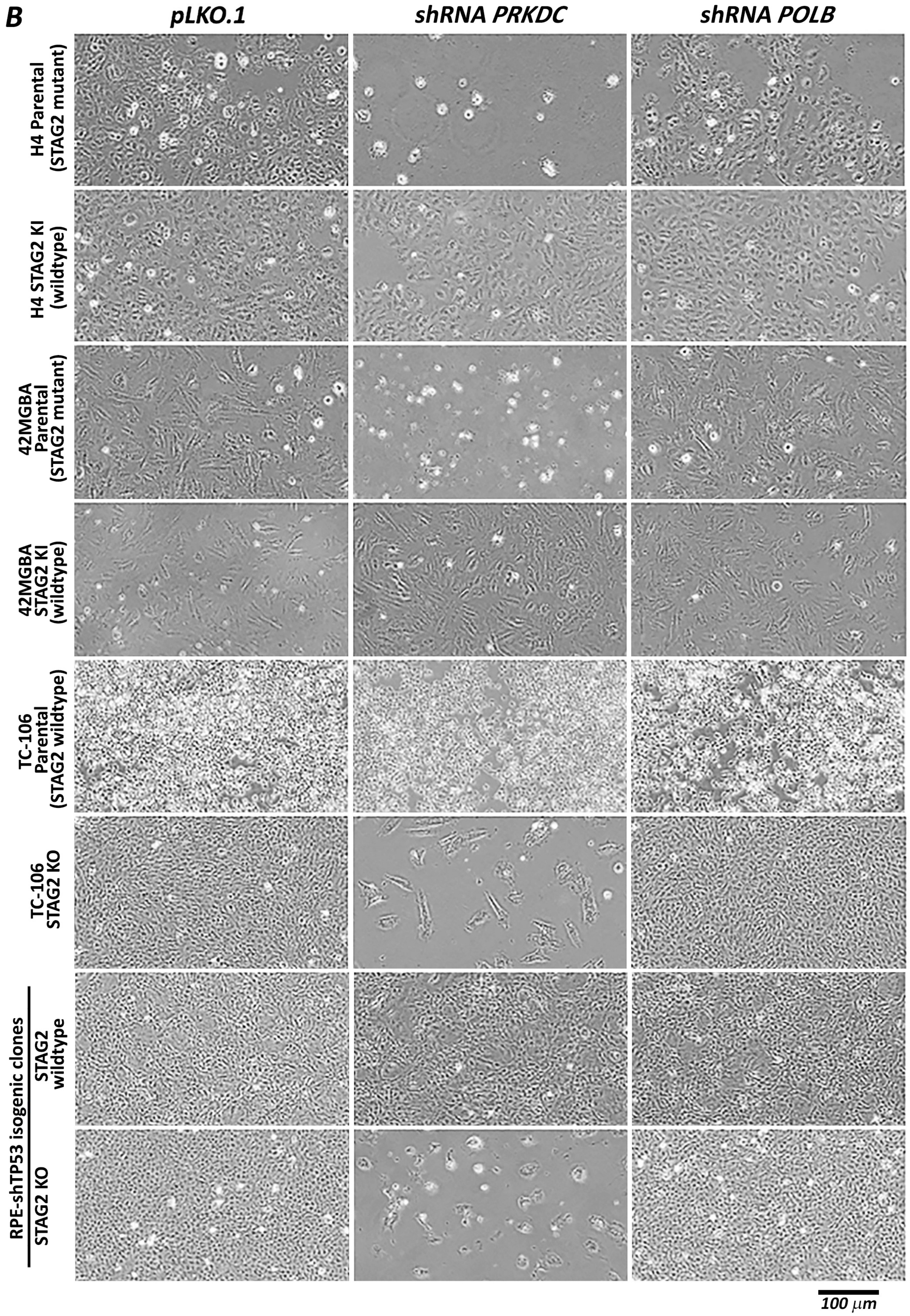

Supplementary Figure 7

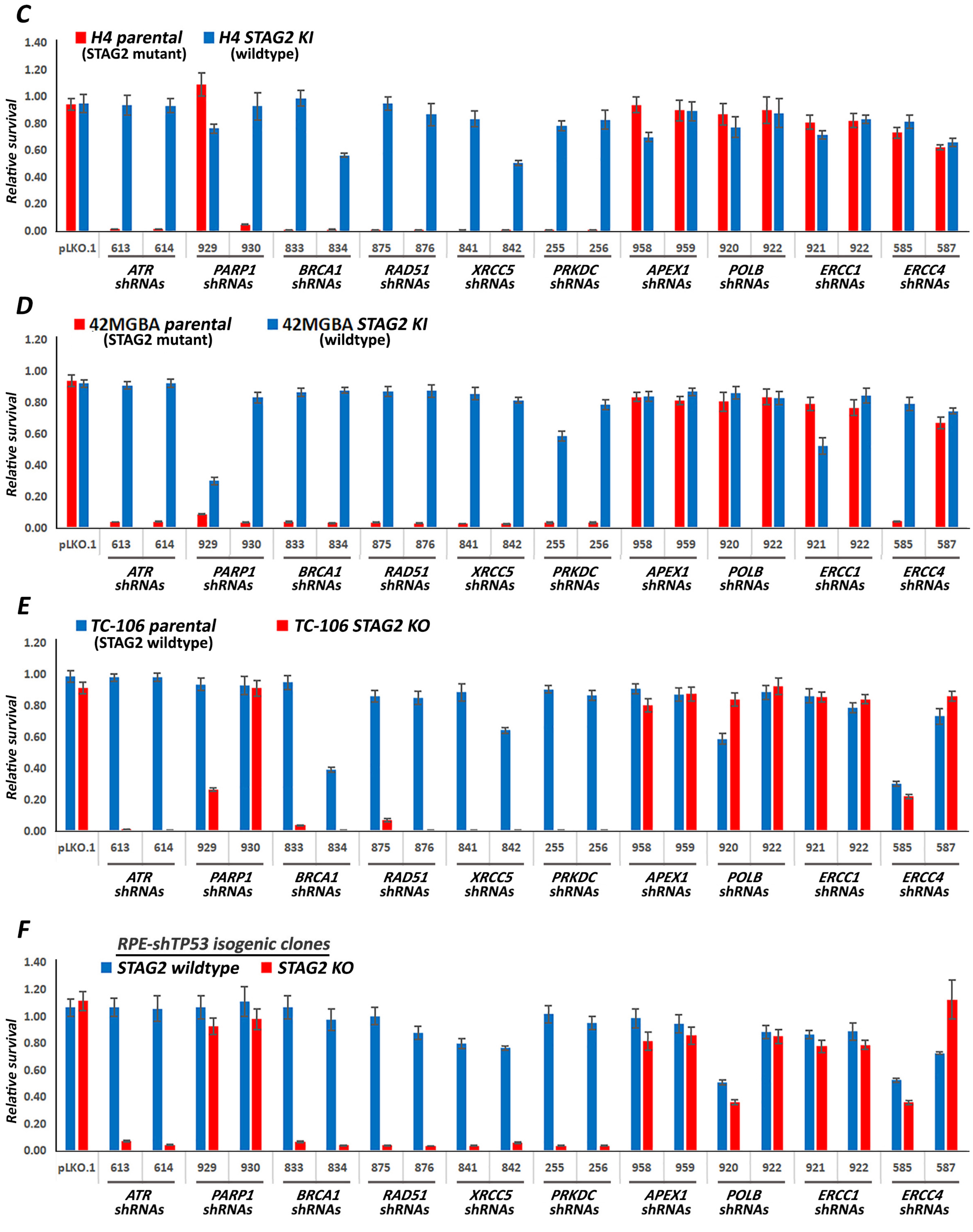

Supplementary Figure 7

G

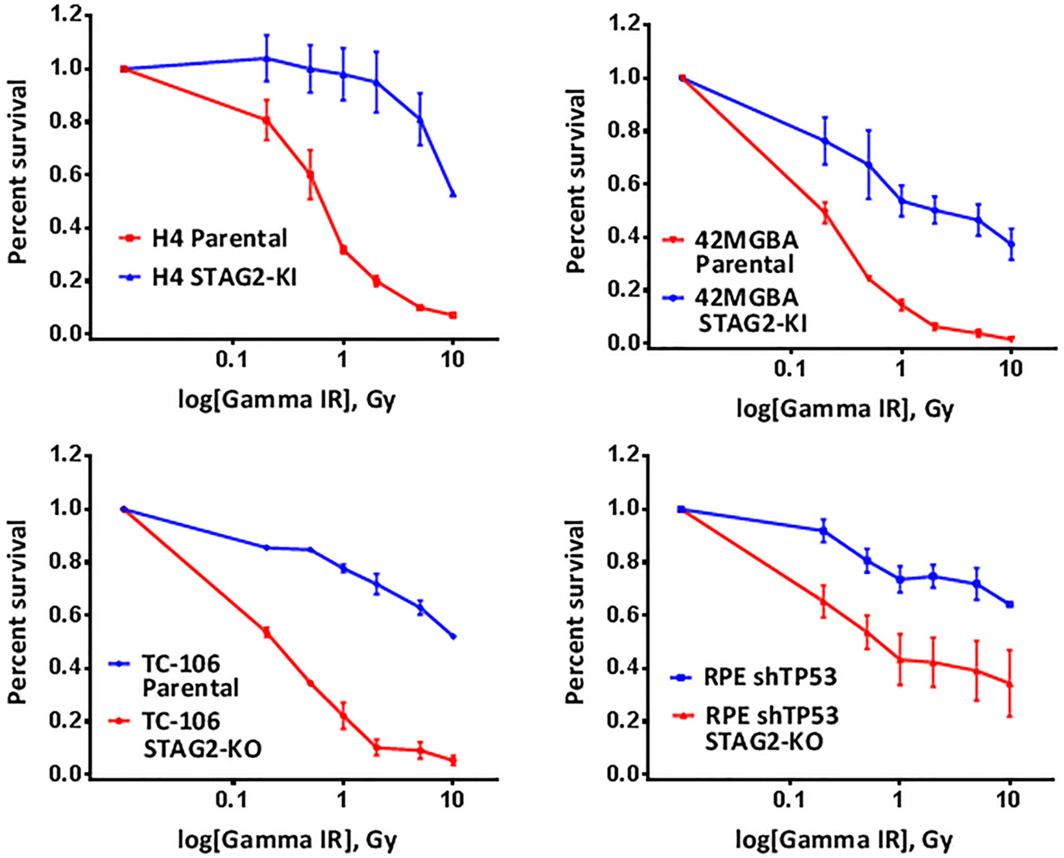

H

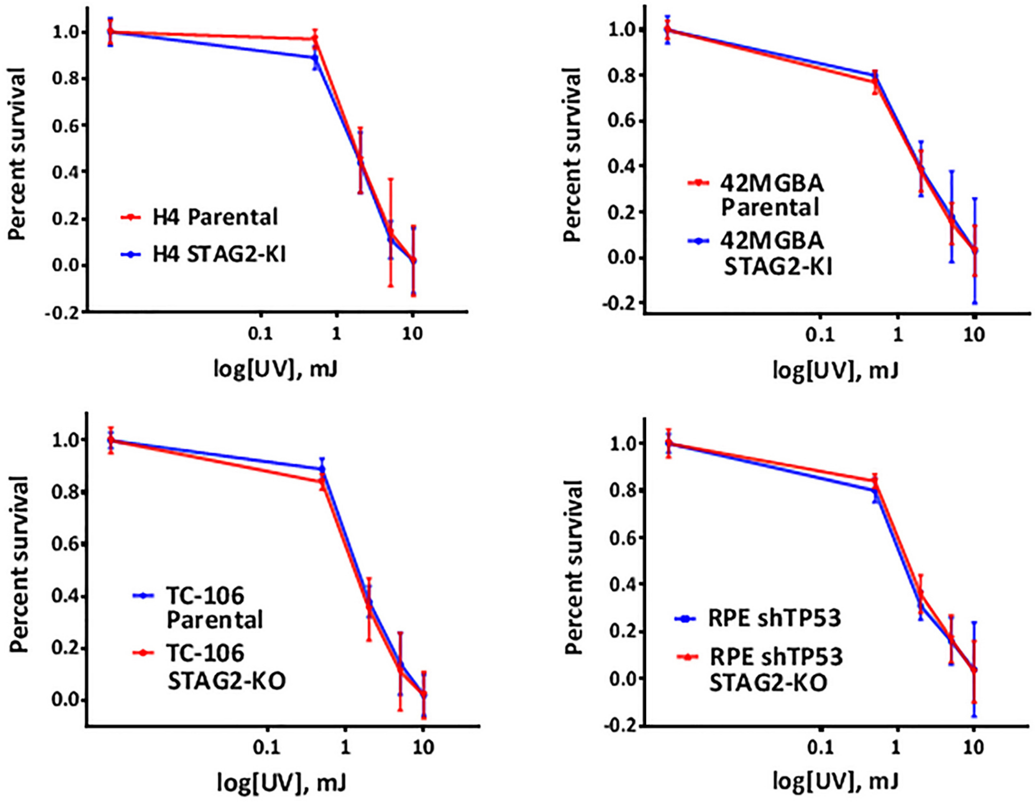

Supplementary Figure 7

Supplementary Figure 7. *STAG2* deficiency in human cancer cells creates a synthetic lethality with DNA double-strand break repair factors and increased sensitivity to ionizing radiation. **A,** Validation of lentiviral shRNA mediated depletion of DNA repair factors. Shown are immunoblots of total lysate from U87MG cells following lentiviral transduction with empty pLKO.1 or five independent shRNA sequences against each of 10 DNA repair factors. The two shRNAs that produced the most effective protein depletion (highlighted in green) were used in the shRNA synthetic lethality screen. **B,** Representative phase contrast images of *STAG2* isogenic cell pairs showing synthetic lethality of *STAG2* deficiency with shRNA depletion of the non-homologous end joining factor DNA-PKcs (*PRKDC*), but not the base excision repair factor DNA polymerase β (*POLB*). **C-F,** Quantitation of survival from shRNA synthetic lethality screen in *STAG2* isogenic H4 glioblastoma cells (**C**), 42MGBA glioblastoma cells (**D**), TC-106 Ewing sarcoma cells (**E**), and RPE sh*TP53* cells (**F**). Each data point is the mean of 8 replicates from 2 independent experiments, and error bars show standard error of the mean. **G-H,** Survival plots of four *STAG2* isogenic cell pairs following gamma-irradiation (**G**) or ultraviolet irradiation (**H**). Each data point is the mean of 12 replicates from 3 independent experiments, and error bars show standard error of the mean.

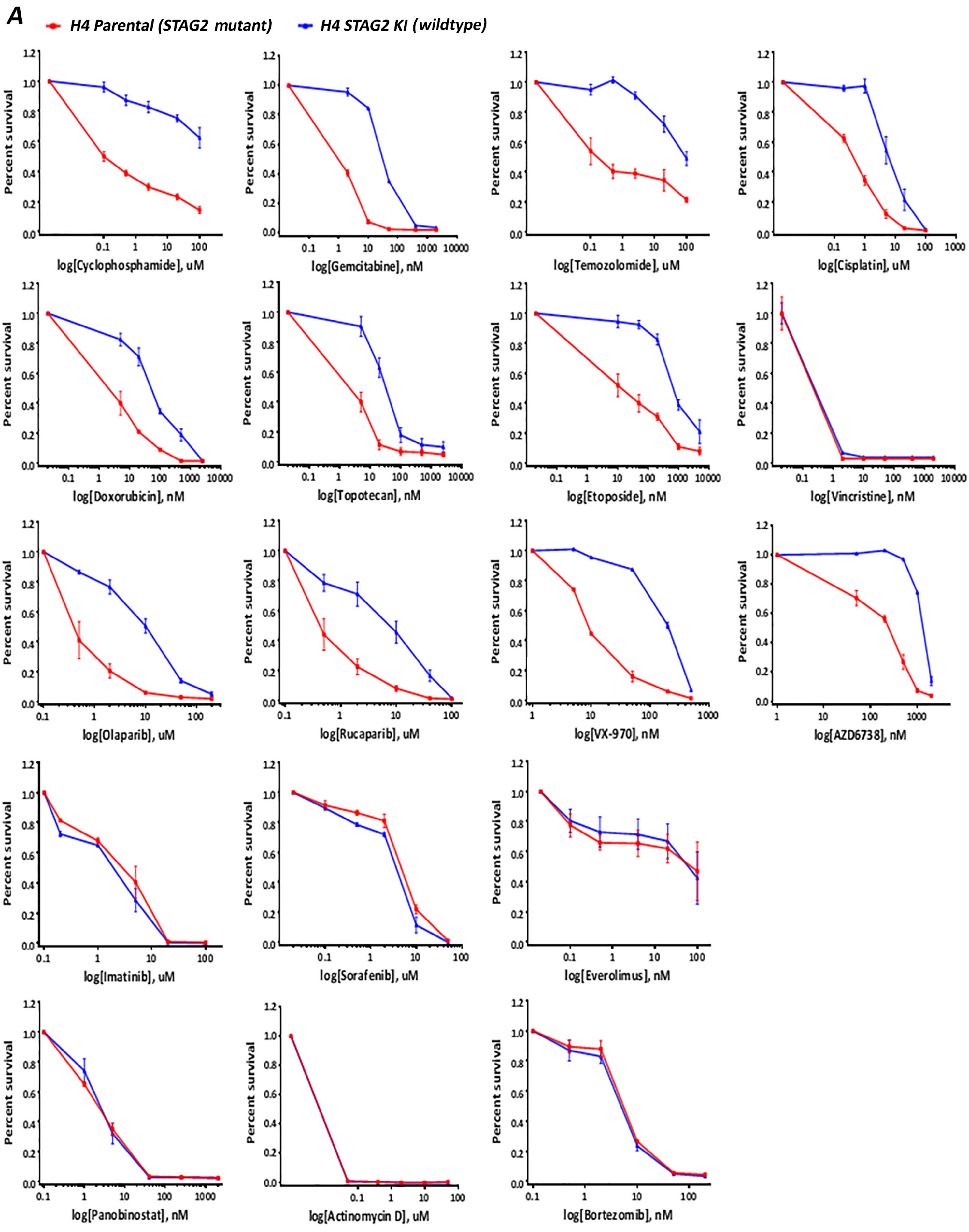

Supplementary Figure 8

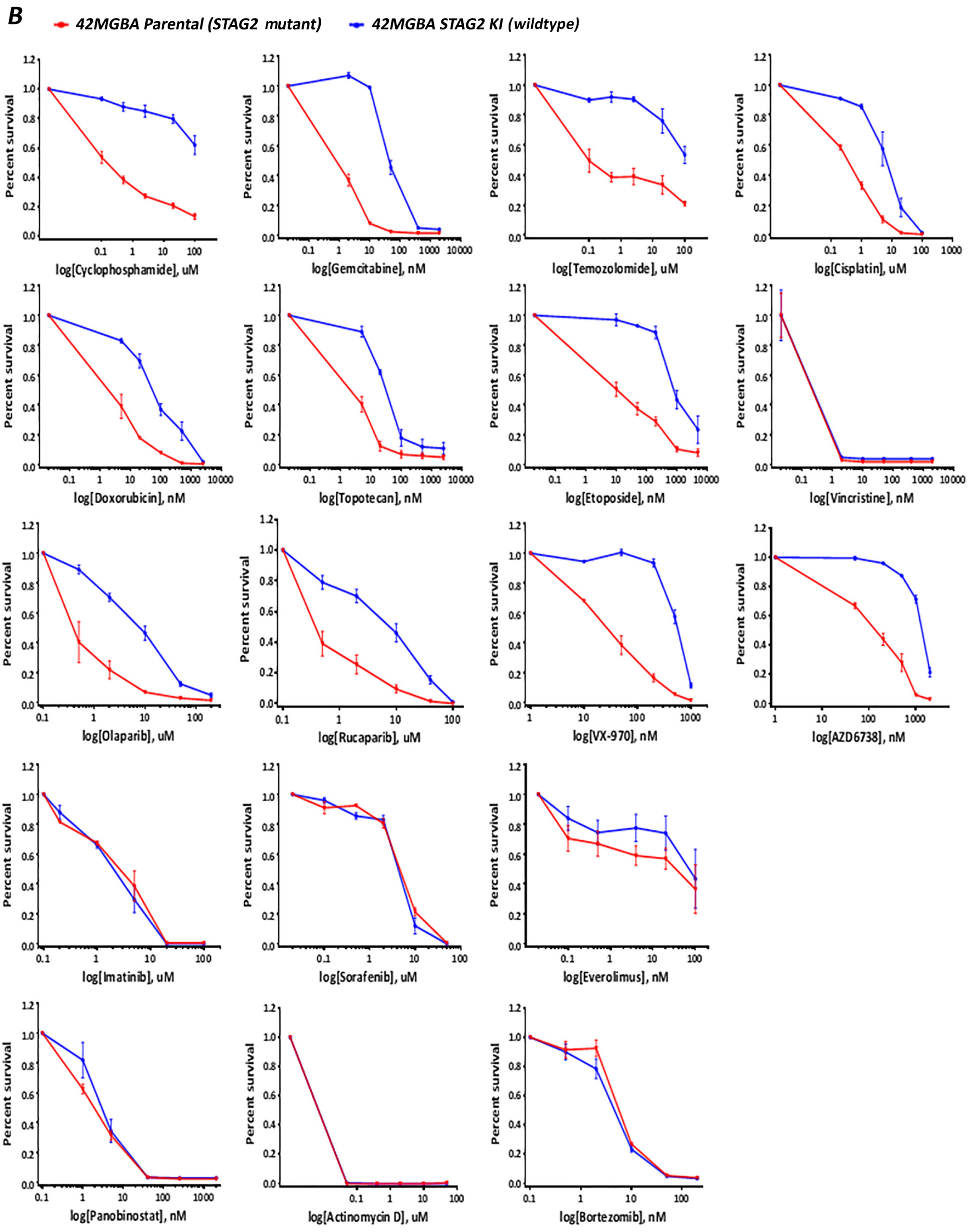

Supplementary Figure 8

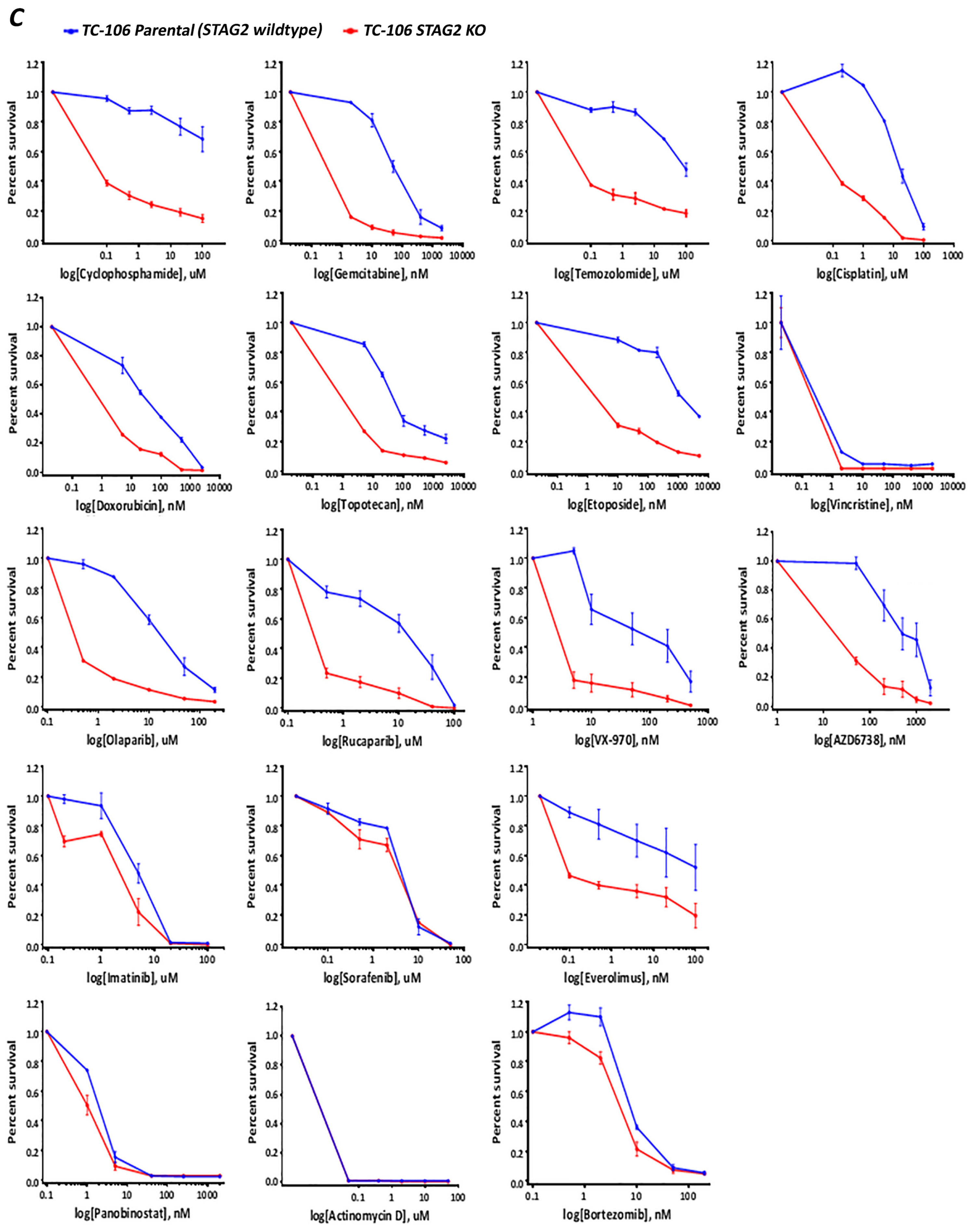

Supplementary Figure 8

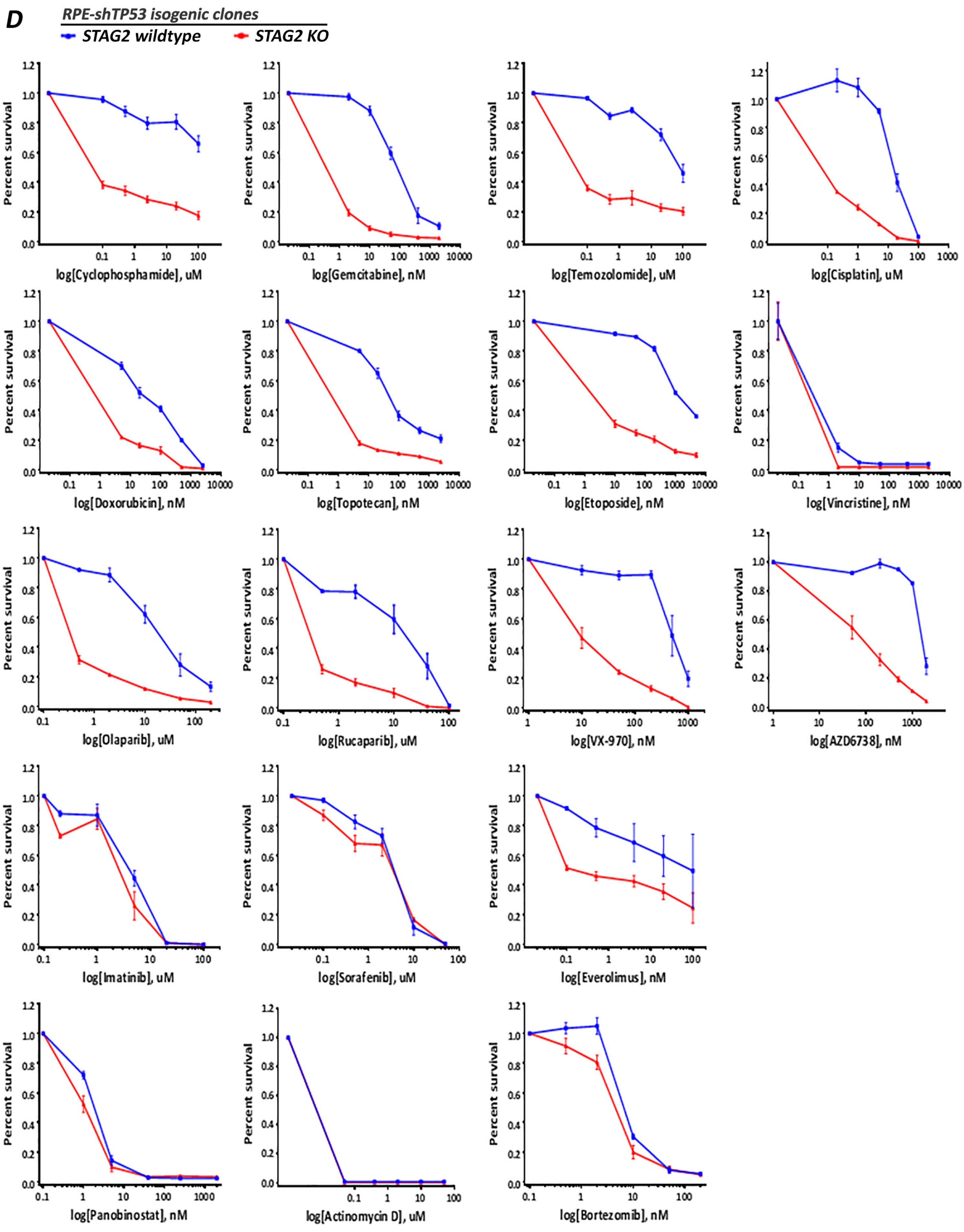

Supplementary Figure 8

E

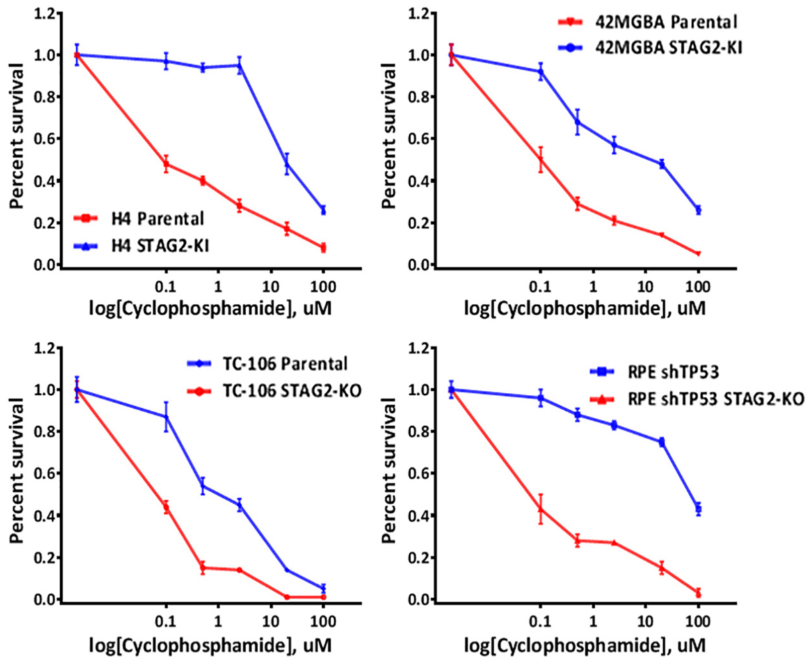

F

H4 STAG2 KI **(wildtype)**

H4 Parental **(STAG2 mutant)**

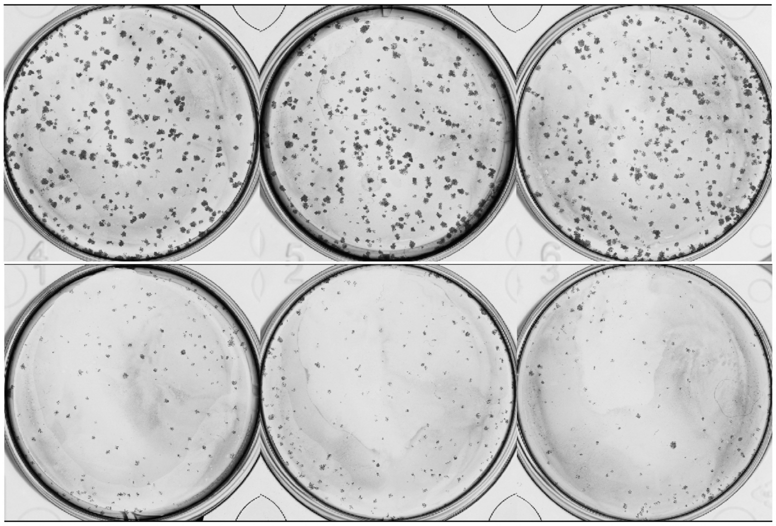

20 μM Cyclophosphamide

Supplementary Figure 8. *STAG2* deficient cancer cells harbor increased sensitivity to cytotoxic chemotherapeutic agents and small molecule inhibitors of DNA double-strand break repair. **A-D,** Survival plots of *STAG2* isogenic H4 glioblastoma cells (**A**), 42MGBA glioblastoma cells (**B**), TC-106 Ewing sarcoma cells (**C**), and RPE sh*TP53* cells (**D**) following treatment with 18 chemotherapeutic agents and small molecule inhibitors. Each data point is the mean of 12 replicates from 3 independent experiments, and error bars show standard error of the mean. **E,** Clonogenic survival assay of four pairs of *STAG2* isogenic human cells upon culture in the presence of the alkylating agent cyclophosphamide. Each data point is the mean of 3 replicates, and error bars show standard error of the mean. **F,** Crystal violet staining of clonogenic survival assay of *STAG2* isogenic H4 glioblastoma cells upon culture in the presence of 20 μM cyclophosphamide.

Supplementary Table 1. Cell lines used in this study.

Cell Line	Description	*STAG2* Status	Source
RPE hTERT	Human hTERT-immortalized retinal pigmented epithelium	Wildtype	ATCC (CRL-4000)
RPE hTERT shTP53	Human hTERT-immortalized retinal pigmented epithelium	Wildtype	This study
RPE hTERT shTP53 STAG2 KO	Human hTERT-immortalized retinal pigmented epithelium	CRISPR-induced frameshift mutation	This study
BJ-5ta	Human hTERT-immortalized skin fibroblasts	Wildtype	ATCC (CRL-4001)
SVG p12	Human SV40-immortalized fetal astroglial cells	Wildtype	ATCC (CRL-8621)
TC-106	Human Ewing sarcoma	Wildtype	Dr. Timothy Triche (CHLA)
TC-106 STAG2 KO	Human Ewing sarcoma	CRISPR-induced frameshift mutation	This study
TC-71	Human Ewing sarcoma	Wildtype	Dr. Jeffrey Toretsky (Georgetown)
EW2	Human Ewing sarcoma	Wildtype	Dr. Suzanne Baker (St. Jude)
H4	Human glioblastoma	Mutant (frameshift)	ATCC (HTB-148)
H4 STAG2 KI	Human glioblastoma	Wildtype (corrected by homologous recombination)	Solomon et al. *Science* 2011
42MGBA	Human glioblastoma	Mutant (nonsense)	DSMZ (ACC 431)
42MGBA STAG2 KI	Human glioblastoma	Wildtype (corrected by homologous recombination)	Solomon et al. *Science* 2011
U87MG	Human glioblastoma	Wildtype	ATCC (HTB-14)
293T	Human embryonic kidney	Wildtype	ATCC (CRL-3216)

Supplementary Table 2. CRISPR guides, primers, and lentiviral shRNAs used in this study.

Target	Backbone	CRISPR Guide #	Guide Sequence	Source
STAG2	pCas-Guide-EF1a-GFP	GE200414D	GATGACAGCTTTGGTGAATG	Origene

Target	Primer Name	for CRISPR Guide #	Primer Sequence	Source
STAG2	STAG2 Fwd	GE200414D	TCAATTTTGCCGTGAATGAA	IDT
	STAG2 Rev	GE200414D	TCCGCTTTTGTAGCAGGAGT	IDT

Target	Backbone	shRNA #	shRNA Sequence	Used in functional studies
Empty Vector	pLKO.1	N/A	N/A	yes
Empty Vector	pGIPZ	N/A	N/A	yes
STAG2	pLKO.1	TRCN000015**1221**	AAACAAAGCTGTAAGAACTGC	-
	pLKO.1	TRCN000015**3782**	ATTTCGGTAAGACATCAGTGG	-
TP53	pLKO.1	TRCN000000**3755**	TTCTGGGAGCTTCATCTGGAC	yes
	pLKO.1	TRCN000000**3756**	ATGTAGTTGTAGTGGATGGTG	yes
ATR	pLKO.1	TRCN0000039**613**	ATTGAACAGATACAACCACAG	yes
	pLKO.1	TRCN0000039**614**	TTTACATCTGTAAGTATCCGG	yes
	pLKO.1	TRCN0000039**615**	TAATGTTAGAAGATTAGCGGC	-
	pLKO.1	TRCN0000039**616**	ATAGGCTAGAAATACTTTGGC	-
	pLKO.1	TRCN0000039**617**	TTTGGGTTGTAAATAATCAGC	-
PARP1	pLKO.1	TRCN0000007**928**	AAGGCAGACATTCTAACGAAG	-
	pLKO.1	TRCN0000007**929**	AATCTTCGGTTATGAAGCTGC	yes
	pLKO.1	TRCN0000007**930**	TTGAGGTAAGAGATTTCTCGG	yes
	pLKO.1	TRCN0000007**932**	TTGATGTTCCAGATCAGGTCG	-
BRCA1	pLKO.1	TRCN0000039**833**	TTTAGAGAAGTAAACTTAGGG	yes
	pLKO.1	TRCN0000039**834**	ATTCAGTACAATTAGGTGGGC	yes
	pLKO.1	TRCN0000039**835**	AATTCAGTACAATTAGGTGGG	-
	pLKO.1	TRCN0000039**836**	AATGATGGGCATTTAGAAGGG	-
	pLKO.1	TRCN0000039**837**	ATCTCGTACTTTCTTGTAGGC	-
RAD51	pLKO.1	TRCN0000018**875**	TTATCTTGAGTTAGTCTTAGC	yes
	pLKO.1	TRCN0000018**876**	TAGTCTGTTCTGTAAAGGGCG	yes
	pLKO.1	TRCN0000018**877**	AATGGCGAACATAGCTTCAGC	-
	pLKO.1	TRCN0000018**878**	TAACCGTGAAATGGGTTGTGG	-
	pLKO.1	TRCN0000018**879**	AATCTGTATGATCTCTGACCG	-
XRCC5 (Ku80)	pLKO.1	TRCN0000039**838**	TAATCACATCACAAGGGCTGC	-
	pLKO.1	TRCN0000039**839**	TTTCAGGAAGTTGTTAAAGCG	-
	pLKO.1	TRCN0000039**840**	AAAGGCAAGAAGAATTGCAGG	-
	pLKO.1	TRCN0000039**841**	ATAGTTATGCTTGATATGAGG	yes
	pLKO.1	TRCN0000039**842**	TTACTCATGGTAAAGCCCACG	yes
PRKDC (DNA-PKcs)	pLKO.1	TRCN0000006**255**	AATGTAATGCTTTCTATCTGC	yes
	pLKO.1	TRCN0000006**256**	TAGAATTAGGATCTTTACCGG	yes
	pLKO.1	TRCN0000006**257**	AATGATTCAGACTTTCACTGG	-
	pLKO.1	TRCN0000006**258**	AATACAAGAGACAAAGGGTGG	-
	pLKO.1	TRCN0000006**259**	ATGTCTTTGTAATAAGGCTGC	-
APEX1	pLKO.1	TRCN0000007**958**	AATAGAATGCAGATTTCTCTG	yes
	pLKO.1	TRCN0000007**959**	TATTGATGAGAGAGTCCAGGC	yes
	pLKO.1	TRCN0000007**960**	AATGCAGGTAACAGAGAGTGG	-
	pLKO.1	TRCN0000007**961**	ATCCTTTCTTCTTAATCCAGG	-
	pLKO.1	TRCN0000011**214**	ATTCAGCCACAATCACCCGGC	-
POLB (DNA Polb)	pLKO.1	TRCN0000007**919**	TACTCCAGGCAATTTCTTAGC	-
	pLKO.1	TRCN0000007**920**	TTGATTCTGAAGTGAAGCTGG	yes
	pLKO.1	TRCN0000007**921**	TTTGTCTCACCCTTTGACAGG	-
	pLKO.1	TRCN0000007**922**	AAAGTTTGCGAGTTCTGTGAG	yes
	pLKO.1	TRCN0000011**213**	TTCATCTACAAACTTCCTTGC	-
	pLKO.1	TRCN0000049**918**	TAGATCGGAATAAGGGCTTGG	-

ERCC1	pLKO.1	TRCN0000049**919**	TTACGTCGCCAAATTCCCAGG	-
	pLKO.1	TRCN0000049**920**	ATAAGGCCAGATCTTCTCTTG	-
	pLKO.1	TRCN0000049**921**	AGGATACACATCTTAGCCAGC	yes
	pLKO.1	TRCN0000049**922**	AATTTCTTCCTTGCTGGCGGC	yes
ERCC4 (XPF)	pLKO.1	TRCN0000078**583**	AAATCACTGATACTCTTGCGC	-
	pLKO.1	TRCN0000078**584**	AAGACTCGATTATTCTGTGGG	-
	pLKO.1	TRCN0000078**585**	ATAGCAGTCTGTATAGCAAGC	yes
	pLKO.1	TRCN0000078**586**	TTGAGTTAAGGTCAACTTCCG	-
	pLKO.1	TRCN0000078**587**	ATAAAGAACCACGTATCTTGG	yes
SMC1A	pLKO.1	TRCN0000062**553**	ATTGATTTCATCAATACGCCG	yes
	pLKO.1	TRCN0000062**554**	AAATGGTCCGATAATCTGTCG	-
	pLKO.1	TRCN0000062**555**	TATACTGAATACAGTCCCGGC	-
	pLKO.1	TRCN0000062**556**	AATGGGTCATTTCCTTGTTGG	yes
	pLKO.1	TRCN0000062**557**	TATAGATCTCATCAATGTTGG	-
SMC3	pLKO.1	TRCN0000160**156**	ATTGAGATAAGTCTCTACTCG	-
	pLKO.1	TRCN0000160**366**	AACTTCTGGAATACTTCACTG	yes
	pLKO.1	TRCN0000160**440**	TTAAGTTCCTGTTCTACTTGG	-
	pLKO.1	TRCN0000159**635**	TTTAATTCTGTGTTGCACTGC	yes
RAD21	pLKO.1	TRCN0000148**110**	TTCTTCAGGTAAAGTAATGGC	yes
	pLKO.1	TRCN0000148**135**	TTGACACTGTCAACAATTAGC	-
	pLKO.1	TRCN0000148**279**	ATGATTTCTGATGCTATCTGG	yes
	pLKO.1	TRCN0000147**898**	AAGCAAACTGATAGATTCAGC	-
STAG1	pLKO.1	TRCN0000140**030**	TAATGAGGTTTCCATTCGGGC	-
	pLKO.1	TRCN0000145**197**	TAATTCCAGCTTCAATTCTGC	-
	pLKO.1	TRCN0000142**642**	TAACTCCAACCTTTCATTGGC	-
	pLKO.1	TRCN0000140**749**	TTTGTGAAGGAACTCTCCAGC	yes
	pLKO.1	TRCN0000144**850**	ATCATCTTCTATGATAGCTGC	yes
STAG2	pGIPZ	V2LHS_198**853**	ATTACAAGCAAATAAATCC	-
	pGIPZ	V2LHS_207**886**	TTGCTAATAACTGAGGAAG	-
	pGIPZ	V2LHS_391**825**	ACATAGTGAGTACACTGCA	yes
	pGIPZ	V2LHS_391**827**	AAATTTCTTCTCTTGTGCA	yes
	pGIPZ	V2LHS_391**829**	TACATAGTGAGTACACTGC	-
	pGIPZ	V2LHS_391**830**	TCAATGAAGACATGATCCA	yes

Supplementary Table 3. Antibodies used in this study.

Primary Antibodies	Estimated Size (kDa)	Host	Antibody #	Vendor	Dilution for immunoblot	Dilution for IP	Dilution for IF
STAG2	141	Mouse	sc-81852 (J-12)	SCBT	(1:100)	(1:25)	-
Beta-Actin	42	Mouse	A5441 (AC-15)	Sigma	(1:2000)	-	-
Alpha-Tubulin	50	Mouse	T5168 (B-5-1-2)	Sigma	(1:2000)	-	-
Alpha-Tubulin	50	Rabbit	ab24246	AbCam	(1:1000)	-	-
53BP1	220	Rabbit	ab21083	AbCam	-	-	(1:1000)
ATR	301	Rabbit	ab2905	AbCam	(1:500)	-	-
PARP1	113	Mouse	sc-8007 (F-2)	SCBT	(1:200)	-	-
BRCA1	208	Mouse	sc-6954 (D-9)	SCBT	(1:200)	-	-
RAD51	37	Rabbit	ab63801	AbCam	(1:500)	-	-
Ku80	83	Rabbit	GTX109935 (N3C2)	GeneTex	(1:500)	-	-
DNA-PKcs	469	Mouse	sc-390698 (E-6)	SCBT	(1:100)	-	-
APEX1	36	Rabbit	ab137708	AbCam	(1:500)	-	-
DNA Pol Beta	38	Rabbit	ab26343	AbCam	(1:500)	-	-
ERCC1	33	Mouse	sc-17809 (D-10)	SCBT	(1:100)	-	-
ERCC4	105	Mouse	sc-136153 (3F2/3)	SCBT	(1:100)	-	-
Phospho-ATM (Ser1981)	350	Rabbit	5883	Cell Signaling	(1:500)	-	-
Phospho-ATR (Ser428)	300	Rabbit	2853	Cell Signaling	(1:500)	-	-
Phospho-BRCA1 (Ser1524)	220	Rabbit	9009	Cell Signaling	(1:500)	-	-
Phospho-Chk2 (Thr68)	62	Rabbit	2197	Cell Signaling	(1:500)	-	-
Phospho-p53 (Ser15)	53	Mouse	9286	Cell Signaling	(1:1000)	-	-
SMC3	142	Rabbit	ab9263	AbCam	(1:500)	(1:50)	-
SMC3	141	Mouse	sc-376352	SCBT	(1:100)	(1:25)	-
RAD21	72	Rabbit	ab992	AbCam	(1:250)	-	-
SMC1A	143	Rabbit	ab9262	AbCam	(1:250)	(1:50)	-
STAG1	144	Mouse	sc-81851	SCBT	(1:100)	-	-
p21	21	Mouse	2946	Cell Signaling	(1:1000)	-	-
p53	53	Rabbit	2527 (7F5)	Cell Signaling	(1:500)	-	-
acetyl-Lysine	-	Rabbit	ab21623	AbCam	(1:1000)	-	-
Anti-BrdU	-	Rat	ab6326 [BU1/75 (ICR1)]	AbCam	-	-	(1:400)
Anti-BrdU	-	Mouse	347580 (B44)	BD Biosciences	-	-	(1:500)
MCM3	91	Mouse	sc-390480	SCBT	(1:100)	-	-
MCM5	82	Rabbit	ab17967	AbCam	(1:200)	-	-
NIPBL	316	Mouse	sc-374625	SCBT	(1:100)	(1:25)	-
PDS5A	151	Rabbit	PA5-57756	Thermo Fisher	(1:200)	(1:50)	-
MAD2L1	23	Rabbit	NBP1-31311	Novus	(1:200)	(1:50)	-
Securin	22	Rabbit	ab3305	AbCam	(1:200)	(1:50)	-
ORC1	97	Mouse	sc-398734 (F-10)	SCBT	(1:100)	-	-
ORC3	82	Mouse	sc-374231 (C-12)	SCBT	(1:100)	-	-
RPA2/p34	29	Mouse	R1280	Sigma	(1:500)	-	-
CDC6	63	Rabbit	3387	Cell Signaling	(1:500)	-	-
CDT1	60	Rabbit	8064	Cell Signaling	(1:500)	-	-
Geminin	25	Rabbit	5165	Cell Signaling	(1:500)	-	-
PCNA	29	Rabbit	13110	Cell Signaling	(1:500)	-	-
DNA Pol Delta	124	Rabbit	ab186407	AbCam	(1:200)	-	-
DNA Pol Epsilon	220	Mouse	sc-12728 (3C5.1)	SCBT	(1:100)	-	-
Cdc45	65	Rabbit	11881	Cell Signaling	(1:500)	-	-
AND1	126	Rabbit	NBP1-89091	Novus	(1:200)	-	-

Secondary Antibodies	Estimated Size (kDa)	Host	Antibody #	Vendor	Dilution for immunoblot	Dilution for IP	Dilution for IF
anti-mouse HRP	-	horse	7076	Cell Signaling	(1:250)	-	-
anti-rabbit HRP	-	goat	7074	Cell Signaling	(1:250)	-	-

anti-mouse Alexa-488	-	goat	A11001	Invitrogen	-	-	(1:500)
anti-mouse Alexa-568	-	goat	A11004	Invitrogen	-	-	(1:500)
anti-rabbit Alexa-488	-	goat	A11008	Invitrogen	-	-	(1:500)
anti-rabbit Alexa-568	-	goat	A11011	Invitrogen	-	-	(1:500)
anti-rat Alexa-488	-	goat	A11006	Invitrogen	-	-	(1:500)
anti-rat Alexa-568	-	goat	A11077	Invitrogen	-	-	(1:500)
anti-mouse-Cy3	-	sheep	C2181	Sigma	-	-	(1:500)

Supplementary Table 4. Drugs used in this study.

Drug Name	Mol Weight	Target/Mechanism	Cat. No.	Vendor.	Solvent
Everolimus	958.22	mTOR Inh.	S1120	Selleckchem	DMSO
VE-822 (VX-970)	463.55	ATR kinase Inh.	S7102	Selleckchem	DMSO
AZD6738	412.51	ATR kinase Inh.	S7693	Selleckchem	DMSO
Cyclophosphamide	279.1	DNA Alkylating	S2057	Selleckchem	DMSO
Temozolomide	194.15	DNA Alkylating	S1237	Selleckchem	DMSO
Etoposide	588.56	Topoisomerase-II Inh.	S1225	Selleckchem	DMSO
Doxorubicin	579.98	Topoisomerase-II Inh.	S1208	Selleckchem	DMSO
Topotecan	457.91	Topoisomerase-I Inh.	S1231	Selleckchem	DMSO
Gemcitabine	263.2	DNA Alkylating	S1714	Selleckchem	DMSO
Cisplatin	300.05	DNA-Crosslinking	S1166	Selleckchem	DMF
Vincristine	923.04	Microtubule Inh.	S1241	Selleckchem	DMSO
Actinomycin D	1255.43	RNA Polymerase Inh.	1229	Tocris	DMSO
Bortezomib	384.24	Proteasome Inh.	S1013	Selleckchem	DMSO
Panobinostat	349.43	HDAC Inh.	S1030	Selleckchem	DMSO
Olaparib	434.46	PARP Inh.	S1060	Selleckchem	DMSO
Rucaparib	421.36	PARP Inh.	S1098	Selleckchem	DMSO
Imatinib	589.71	Tyrosine kinase Inh.	S1026	Selleckchem	DMDO
Sorafenib	637.03	Tyrosine kinase Inh.	S1040	Selleckchem	DMDO
Palbociclib	573.66	CDK4/6 Inh.	S1579	Selleckchem	Water
Aphidicolin	338.48	DNA Polymerase Inh.	A0781	Sigma	Water
Colcemid	371.43	Microtubule Inh.	15210-040	Life Technologies	Water

Supplementary Table 5. Other key reagents used in this study.

Reagent	Product ID	Vendor
Beta-galactosidase assay kit	9860S	Cell Signaling
Cell Titer-Glo reagent	G8462	Promega
iBind Flex Solution Kit	SFL2020	Invitrogen
MES SDS Running Buffer	B0002	Invitrogen
Bolt Bis-Tris Plus gels	NW04120/22	Invitrogen
iBlot2 NC Mini Stacks	IB23002	Invitrogen
iBind Flex Card	SFL2010	Invitrogen
NuPAGE LDS Sample Buffer (4X)	NP0007	Invitrogen
Bolt Sample reducing agent (10X)	B0009	Invitrogen
RIPA Buffer	QJ222447	Pierce
Protease and phosphatase Inhibitor	88668	Pierce
Protease Inhibitor	88666	Pierce
5-Iodo-2'-deoxyuridine (IdU)	I7125	Sigma
ImmEdge pen	H-4000	Vector Labs
5-Chloro-2'-deoxyuridine (CldU)	C6891	Sigma
Propidium Iodide	81845	Sigma
RNAse A	R6148	Sigma
Polybrene	TR-1003-G	Millipore
BCA protein assay kit	23227	Pierce
Super signal west pico (ECL)	34078	Thermo scientific
IP Lysis Buffer	87787	Pierce
Protein A sepharose	ab193256	Abcam
BSA	A4503	Sigma
Puromycin	P8833	Sigma
Ampicilin	A0166	Sigma
Kanamycin	K1377	Sigma
KaryoMAX Colcemid	15210-040	Life Technologies
DMEM	10566-016	Life Technologies
Fetal bovine serum	FP-0500-A	Atlas Biologicals
Penicillin/streptomycin	15140163	Life Technologies
FuGENE 6	E2691	Promega
HiSpeed plasmid midi kit	12643	Qiagen
Phenol-Chloroform	15593-049	Invitrogen
Proteinase K	3115801001	Roche
MyFi DNA polymerase	BIO-21118	BioLine
ExoSAP-IT	AF78201	Affymetrix
Vectashield Mounting Medium with DAPI	H-1200	Vector Labs
Vectashield Mounting Medium	H-1000	Vector Labs
DNase-I	M0303S	NEB
Coverslip sealant	23005	Biotium
Micrococcal Nuclease	88216	Thermo scientific
