# Supplementary material for: A requirement for STAG2 in replication fork progression creates a targetable synthetic lethality in cohesin-mutant cancers
Source: Nat Commun. 2019 Apr 11;10:1686. doi: 10.1038/s41467-019-09659-z (PMC6459917; doi:10.1038/s41467-019-09659-z)
Supplement: Supplementary file 3 — Reporting Summary [file 41467_2019_9659_MOESM3_ESM.pdf]

nature research

Corresponding author(s): David A. Solomon, MD, PhD, Assistant Professor, Department of Pathology, University of California, San Francisco

Reporting Summary

Nature Research wishes to improve the reproducibility of the work that we publish. This form provides structure for consistency and transparency in reporting. For further information on Nature Research policies, see Authors & Referees and the Editorial Policy Checklist.

Statistical parameters

When statistical analyses are reported, confirm that the following items are present in the relevant location (e.g. figure legend, table legend, main text, or Methods section).

n/a	Confirmed	
☐	☒	The exact sample size (n) for each experimental group/condition, given as a discrete number and unit of measurement
☐	☒	An indication of whether measurements were taken from distinct samples or whether the same sample was measured repeatedly
☐	☒	The statistical test(s) used AND whether they are one- or two-sided<br>*Only common tests should be described solely by name; describe more complex techniques in the Methods section.*
☒	☐	A description of all covariates tested
☒	☐	A description of any assumptions or corrections, such as tests of normality and adjustment for multiple comparisons
☒	☐	A full description of the statistics including central tendency (e.g. means) or other basic estimates (e.g. regression coefficient) AND variation (e.g. standard deviation) or associated estimates of uncertainty (e.g. confidence intervals)
☒	☐	For null hypothesis testing, the test statistic (e.g. F, t, r) with confidence intervals, effect sizes, degrees of freedom and P value noted<br>*Give P values as exact values whenever suitable.*
☒	☐	For Bayesian analysis, information on the choice of priors and Markov chain Monte Carlo settings
☒	☐	For hierarchical and complex designs, identification of the appropriate level for tests and full reporting of outcomes
☒	☐	Estimates of effect sizes (e.g. Cohen's d, Pearson's r), indicating how they were calculated
☐	☒	Clearly defined error bars<br>*State explicitly what error bars represent (e.g. SD, SE, CI)*

Our web collection on statistics for biologists may be useful.

Software and code

Policy information about availability of computer code

Data collection	BD FACSDiva software (BD Biosciences) was used to collect flow cytometry data. Leica Application Suite X software (Leica) was used to acquire confocal microscopy imaging data. The software used is described in the Methods.
Data analysis	FlowJo software (BD Biosciences) was used to analyze flow cytometry data. GraphPad Prism and Microsoft Excel were used for statistical analysis and data presentation. The software used is described in the Methods.

For manuscripts utilizing custom algorithms or software that are central to the research but not yet described in published literature, software must be made available to editors/reviewers upon request. We strongly encourage code deposition in a community repository (e.g. GitHub). See the Nature Research guidelines for submitting code & software for further information.

Data

Policy information about availability of data

All manuscripts must include a data availability statement. This statement should provide the following information, where applicable:

- Accession codes, unique identifiers, or web links for publicly available datasets
- A list of figures that have associated raw data
- A description of any restrictions on data availability

Source data files including uncropped Western blots and all underlying data for graphs and tables is included in the Supplementary Online Material.

Field-specific reporting

Please select the best fit for your research. If you are not sure, read the appropriate sections before making your selection.

☒ Life sciences ☐ Behavioural & social sciences ☐ Ecological, evolutionary & environmental sciences

For a reference copy of the document with all sections, see nature.com/authors/policies/ReportingSummary-flat.pdf

Life sciences study design

All studies must disclose on these points even when the disclosure is negative.

Sample size	Two independent shRNA sequences against STAG2 were utilized throughout all experiments in this study to ensure that all cellular phenotypes associated with STAG2 deficiency are attributable to gene specific knockdown effects. Two independent shRNA sequences against each of the cohesin subunits was utilized to assess cellular phenotypes associated with cohesin deficiency presented in Figure 1. Two independent shRNA sequences against of the DNA repair genes was utilized in the synthetic lethality screen presented in Figure 7. The cellular phenotypes of STAG2 depletion via lentiviral shRNA transduction were performed in three independent immortalized, non-transformed human cell lines (RPE, BJ, and SVG). The synthetic lethality study with DNA repair factors presented in Figure 7 was performed in four STAG2 isogenic cell line pairs including multiple different tumor origins (glioblastoma, Ewing sarcoma, and epithelial cells). The radiation and drug sensitivity assays presented in Figures 7 and 8 was performed in four STAG2 isogenic cell line pairs including multiple different tumor origins (glioblastoma, Ewing sarcoma, and epithelial cells).
Data exclusions	No data were excluded from any of the experimental studies presented in the manuscript.
Replication	All experimental data shown in the manuscript is the result of multiple independent experiments, as detailed in the figure legends and Methods. No data were excluded from the analysis.
Randomization	Not relevant to this study. Our study studies the effect of STAG2 inactivation in human cells. It does not include any population based data or other data type for which randomization is relevant.
Blinding	As described in the Methods regarding the single fiber DNA replication fork analysis presented in Figure 2 and Supplementary Figure 2: "Greater than 200 individual replication forks were evaluated from more than 20 fields from two independent experiments per condition. Evaluation and quantification was performed in a blinded fashion to prevent bias."

Reporting for specific materials, systems and methods

Materials & experimental systems

n/a	Involved in the study
☐	☒ Unique biological materials
☐	☒ Antibodies
☐	☒ Eukaryotic cell lines
☒	☐ Palaeontology
☒	☐ Animals and other organisms
☒	☐ Human research participants

Methods

n/a	Involved in the study
☒	☐ ChIP-seq
☐	☒ Flow cytometry
☒	☐ MRI-based neuroimaging

Unique biological materials

Policy information about availability of materials

Obtaining unique materials	All unique biological materials (e.g. STAG2 isogenic human cancer cell lines) are readily available from the authors upon request.

Antibodies

Antibodies used	The complete list of primary and secondary antibodies used including supplier name, catalog number, and clone name are provided in Supplementary Table 1.
Validation	Validation data for each of the primary antibodies used are provided on the manufacturer's website, along with citation references and application specific technical details. No new antibodies were generated as part of this study.

Eukaryotic cell lines

Policy information about cell lines

Cell line source(s)	The source of each of the cell lines used in this study is provided in Supplementary Table 1.
Authentication	None of the cell lines used were authenticated by STR fingerprinting analysis. Western blot confirmation of STAG2 status was confirmed for all STAG2 isogenic cell pairs used in this study.
Mycoplasma contamination	Cell lines were not tested for Mycoplasma contamination.
Commonly misidentified lines (See ICLAC register)	No commonly misidentified cell lines were used in this study.

Flow Cytometry

Plots

Confirm that:

☒ The axis labels state the marker and fluorochrome used (e.g. CD4-FITC).

☒ The axis scales are clearly visible. Include numbers along axes only for bottom left plot of group (a 'group' is an analysis of identical markers).

☒ All plots are contour plots with outliers or pseudocolor plots.

☒ A numerical value for number of cells or percentage (with statistics) is provided.

Methodology

Sample preparation	As listed in the Methods section of the manuscript: "RPE, BJ, and SVG p12 cells either synchronized with drugs or treated with lentiviral shRNA conditioned media were collected by trypsinization and washed with PBS. Cells were re-suspended and fixed with 70% ethanol at 4oC for 1 hour. Fixed cells were washed in PBS and stained with propidium iodide (50 [illegible]g/mL) containing RNAse (100 ug/mL) for 30 minutes in the dark. 15,000 stained cells per experimental condition were assessed using a BD LSRFortessa cell analyzer and BD FACSDiva software (BD Biosciences). The following drugs were used: DMSO vehicle, aphidicolin (2 ug/mL, for 48 hours), palbociclib (1 uM, for 48 hours), colcemid (0.5 ug/mL, for 16 hours). Flow cytometric analysis was performed following treatment with lentiviral shRNAs at multiple time points as indicated."
Instrument	As listed in the Methods section of the manuscript: "15,000 stained cells per experimental condition were assessed using a BD LSRFortessa cell analyzer and BD FACSDiva software (BD Biosciences)."
Software	As listed in the Methods section of the manuscript: "15,000 stained cells per experimental condition were assessed using a BD LSRFortessa cell analyzer and BD FACSDiva software (BD Biosciences)." FlowJo software (BD Biosciences) was used for analysis and presentation of the flow cytometry data.
Cell population abundance	No cell sorting was performed. All flow cytometry analysis presented in the study is assessment of propidium iodide staining intensity only, with no cells gated or excluded from the analysis.
Gating strategy	No gating or cell sorting was performed. All flow cytometry analysis presented in the study is assessment of propidium iodide staining intensity only, with no cells gated or excluded from the analysis.

☒ Tick this box to confirm that a figure exemplifying the gating strategy is provided in the Supplementary Information.
