# Supplementary material for: A requirement for STAG2 in replication fork progression creates a targetable synthetic lethality in cohesin-mutant cancers
Source: Nat Commun. 2019 Apr 11;10:1686. doi: 10.1038/s41467-019-09659-z (PMC6459917; doi:10.1038/s41467-019-09659-z)
Supplement: Supplementary file 2 — Peer Review File [file 41467_2019_9659_MOESM2_ESM.pdf]

Reviewers' comments:

Reviewer #1 (Remarks to the Author):

The manuscript by Mondal et al, identifies a role for STAG2 in replication fork progression. They deplete STAG2 in human primary cells that express telomerase and show a dramatic effect: intra-S phase cell cycle arrest shown by FACS and senescence shown by B-gal activity. They use DNA combing to show that loss of STAG2 causes fork stalling. They show that loss of STAG2 leads to impaired SMC3 acetylation and changes in association of SMC3 with replication proteins. They further demonstrate that STAG2 absence leads to fork collapse and DNA damage activation of the checkpoint. They propose that cohesin associates with the pre-replication complex and replication machinery to stabilize the fork and this is disrupted by STAG2 loss. They then performed an shRNA-based synthetic lethality screen and identify genes in the HR and NHEJ pathway. They confirmed a role for STAG2 in this pathway by demonstrating sensitivity of STAG2 mutant cells to gamma-irradiation. They then expose STAG2 mutant cells to a range of chemotoxic agents and find sensitivity, specifically to those agents that induce DS breaks, thereby laying the ground work for future clinical trials for STAG2 cancers.

This is an interesting and important study and much of the data is well presented. However, the strong phenotype of the STAG2 depleted cells is difficult to reconcile with other parts of the paper and the published literature. Moreover, while the synthetic lethal screen is well done with multiple isogenic cells lines, as is the analysis of STAG2 mutant cell response to genotoxic agents (including PARP inhibitor), a previous study has shown that the same STAG2 cell lines cells are sensitive to PARP inhibitors and other DNA damaging agents, thereby diminishing the novelty of the result.

Major concerns are indicated below

1. My concern is with the non-transformed STAG2 depleted cell lines used throughout the paper. The STAG2 depleted cells shown in Fig. 1D and E have a strong S phase arrest, limited growth, and B-gal positive staining, taken as an indicator of senescence. What day of growth are these cells at? The growth defect seems so severe, it’s hard to imagine how these cells can be passaged for 60 Days as shown in Fig. 1C and how they can provide enough material for the experiments shown? These cells look like they are dying. It would be important to know at what day of growth each experiment is done and how these cells come out of S phase arrest to cycle.

2. These cells are described as senescent. Usually senescence is a DNA damage induced G1 arrest. These cells may be stressed and inducing a DNA damage response, but it is not clear what the B-gal stain signifies. Can the authors provide an explanation?

3. For the IPs in Figure 2F, it seems that all proteins tested co-IP with SMC3. It is possible that IP with SMC3 brings down anything that is associated with DNA. The only protein that does not co-IP is ORC1, but since the blot is blank it is not clear that the antibody is even working (and it is also not shown in the input blots in Fig. S2 H). To confirm that these factors co-IP with SMC3 and the cohesin ring through protein interaction and not DNA, the IPs should be done in the presence of ethidium bromide. This would rule out that the IPs are simply bringing down all proteins associated with stalled forks.

4. It is hard to reconcile why the STAG2-depleted non-transformed cells are so sick. The authors show that knockdown of p53 has a dramatic effect reversing the growth defect in STAG2 depleted cells and suggest that the effect helps to explain co-occurrence of STAG2 and p53 mutation in Ewing sarcoma. However, there are many examples of STAG2 tumors without p53 mutation - how do these cells grow so well?

5. The synthetic lethality screen and the subsequent tests of survival using genotoxic agents is very convincing; it is done with four isogenic cell lines and is very reproducible. However, a previous study

(Bailey et al, Mol Cancer Ther 2014) showed that STAG2 tumor cells are sensitive to PARP inhibitors and other DNA-damaging agents. In fact, they used the same 2 (of 4) cell lines (42MGBA and H4) used in the current study. While the current study is extensive and well done, the previous work detracts from the novelty.

Reviewer #2 (Remarks to the Author):

Mutations in cohesin subunits especially in STAG2 have been recurrently identified in a number of human cancers. The manuscript by Mondal describes the role of STAG2 in replication fork progression and reports vulnerabilities of STAG2 mutant cells that could be exploited therapeutically. Understanding the mechanistic basis of the tumor suppressive function of STAG2 as well as providing new therapeutic options for STAG2 mutated tumors is clearly important. While the sensitivity to DNA repair factors of cohesin mutated cells has been proposed before, the manuscript provides advances in this direction. The link between STAG2 and fork progression is novel and interesting. The experimental approaches lack some important controls given the extensive use of shRNA-mediated gene function perturbation. Furthermore, some conclusions need additional clarifications and in some cases the data are at odds with other recent reports. In the opinion of this reviewer, these points would need to be properly addressed to substantiate the claims made in the manuscript.

Major points:
- shRNAs, in particular pLKO based constructs, are notorious for off-target effects and have often led to wrong conclusions. The lack of experiments demonstrating decisively on-target effect of perturbations is worrying. This could be rectified by expression of shRNA-insensitive transgenes or engineering of shRNA target sites in the genome. These experiments should be conducted for STAG2 in one or two key experiments in replication progression. Similarly, rescue experiments should be conducted for at least some candidates (e.g: ATR, BRCA1, RAD51, XRCC5 and PRKDC) in the viability setting.

- The very strong selective effect of ATR, BRCA1, RAD51, XRCC5 and PRKDC in STAG2 ko vs STAG2 wild-type cells is quite striking (Figure 5A-E and suppl Figs). In addition to the on/off-target issue (see above), these experiments lack key controls: what is the effect of the depletion of pan-essential genes across the cells used? Without these data and demonstrating the impact of gene depletions of pan essential genes in the wild-type setting, it is hard to conclude selective effects in mutants.

- The recent study by Liu et al., did not detect enhanced sensitivity of STAG2 mutated cells to cisplatin or PARP inhibitors without depleting STAG1 in addition. How can this be reconciled?

- The co-immunoprecipitation of cohesin with DNA replication factors in Figure 2F is not well controlled and described. Using unspecific antibodies as controls is not sufficient. Using cells stably expressing tagged cohesin subunits and untagged cells as controls would improve the experiment and impact of the data. What is the relative IP and CoIP efficiency of the individual proteins? Showing lysate, flow through and precipitated fractions would help here. Is the interaction indirectly mediated by DNA (nuclease digestion)?

- If S phase arrest and fork instability in non-transformed that lack STAG2 is dependent on TP53, this would suggest that STAG2 does not play a direct role in fork stability but rather that lack of STAG2 in non-transformed cells causes genome instability events that require TP53 action for impact. How does this relate to the described binding of STAG2 to replication factors and is the title of the paper still accurate? Across TCGA datasets, is there a significant association of deleterious STAG2 mutations with TP53 alterations?

Minor points:
- Abstract: "… STAG2 is essential for DNA replication fork progression …": this statement should be altered as STAG2 is only required for "normal" fork progression and that only in the absence of TP53.

- Page 3 intro: "… no direct DNA binding motifs within the core cohesin subunits …"; this statement should be modified as a recent paper has identified a DNA binding site in cohesin (PMID: 30109982).

- Figure 2D: RPAp34 foci should be quantified

- Figure S6F: H4 STAG2 KI cells are more strongly affected by cyclophosphamide than H4 parental cells. Is this a wrong labeling?

- The role of the lack of SMC3 lysine acetylation in STAG2 mutant cells is unclear given that H4 cells lack STAG2 but show SMC3 lysine acetylation.

- Supplementary figures are spread over multiple pages

- Given the different origin of cell lines (glioblastoma, RPE cells) used the dose-response experiments in Figure S6, the consistent curve shapes obtained for many drugs across the different cell models is remarkable and surprising. Drug response profiles for a given drug across models are usually impacted by drug exporter activities, genetic background alterations present and the metabolic parameters of cell lines and rarely that consistent.

Reviewer #3 (Remarks to the Author):

For the Authors:
In the manuscript "A requirement for STAG2 in replication fork progression creates a targetable synthetic lethality with DNA repair factors in cohesin-mutant cancers", by Gourish Mondal et al the authors set out to investigate the consequences of inactivating the Cohesin subunit STAG2. STAG2 is one of two STAG proteins associating with the core cohesin ring in mammalian cells and is specifically required for formation of cohesion at the centromere and along arms. This is different from STAG 1 which has a specific telomere cohesion function. STAG2 is also frequently found to be mutated in various cancers.

Any new knowledge gained from their work the authors aim to utilise in finding strategies for therapeutically targeting cancer cells harbouring mutations in STAG2.

This work is very ambitious and through inactivation of STAG2 in multiple ways in several different cell lines they also find mechanisms for STAG2 function that can potentially be useful in targeting tumours with STAG2 mutations. Overall the experiments are well performed and controlled. The results are in essence novel and of importnace for the field. I do however have some minor concerns that I will list as they appear in the manuscript:

Abstract and Introduction:
In the abstract, the statement "therapeutically targeting cohesin mutant cancer cells are unknown" could be rephrased to largely unknown. It would also be good, in my opinion, to mention that the work in this study give some explanations to the counterintuitive finding that STAG2 is essential for replication fork progression and at the same time inactivating mutations in STAG2 are often found in

various cancers, that are in general fast proliferating.

In several places, all through the manuscript, the language needs to be carefully edited. Some sentences are extremely long and complicated, leaving many readers lost if they are at all unfamiliar with the subject. For example, the sentence in the introduction starting on Line 36, with "The cohesion subunits form,… and ends on Line 42!

Line 37, A minor comment; in mammalian cells cohesin is loaded already in telophase.

Line 66, I believe that Liu J et al senior author JD Krantz showed conserved transcriptional dysregulation of gene expression in CdLS patients already 2009 (PMID: 19468298) which could be a better reference here.

Line 72, should be: in four or more

Results:
In general, all through the manuscript, the authors claim that they are using primary human cells. The RPE cells is likely the best cell culture model for studies like this, but are they really non-transformed? They are immortilized with the hTERT. An active telomerase is what characterize many Cancer cells. Therefore, I suggest to be less strong about the statement that non-transformed primary cells are used.

Line 103, Another major concern is the fact that STAG2 is claimed to be a required gene. Regardless of this, experiments are done on cells that have been without STAG2 for at least 8 days after shRNA treatment? As can be judged from the Western blot the knock down is also extremely efficient. How many things are not happening in these cells during the 8 days. How many cell divisions would the cells go through normally and what is the genomic characteristics after this time in the absence of STAG2? This should at least be discussed or somehow tested? How soon is the senescent stage or proliferative arrest appearing? How can the cells be amplified for collection of genomic DNA for sequencing if they are not dividing?

Line 107 and onwards, it is claimed that STAG2 is inactivated by shRNA in three cell lines. Results are shown only for RPE cells, where are the data from BJ and SVG p12 cells, that are claimed to show the same result? Also, the time point for FACS analysis and microscopy should be indicated (as is stated in M&M).
Line 132 and onwards, give a reference for the accumulation of RPAp34 on stalled replication forks
Line 157, potentially a weak MCM3 band is actually seen in the NIPBL IP Blot Figure S2, in RPE cells, comment?

Line 160, I would say that the interaction between SMC3 and PolD is reduced not disrupted.
Line 175 and onwards, indicate which cell line is used for the G1 arrest exp. Also show a FACS profile for the degree of G1 arrest and S phase accumulation after release from CDK4/6 inhibitor.

Line 179-180, why is it important to say that STAG2 depletion in multiple non-transformed human cell lines resulted in ….? When results are shown from two potentially not so non-transformed cell lines?

Figure 3D, Probably more than one blot needed to be run to detect all these proteins? Could the same extract be used for all? How can otherwise one loading control be used?

Line 262 and onwards plus Fig S6F, is the labelling of the plate reversed from the graph H4 STAG2 KI seems to be surviving better (forming more colonies).

Discussion:
The synthetic lethality between KO of SA1 in combination with SA2 could be discussed.
What is the proposed mechanism for TP53 in activation of the intra-S-phase arrest – discuss or suggest.

Reviewer #4 (Remarks to the Author):

In this manuscript Mondal et al explore de function of the STAG2 cohesin subunit in human cells. Authors construct STAG2 null cells and observe a defect in S-phase progression accompanied by cellular senescence. Replication fork defects are evidenced in these cells, in which alterations in the interactions between cohesin subunits and factors involved in replication origin licensing and DNA synthesis are observed. Fork defects correlate with increased signalling of DNA damage, presumably owing to fork collapse. Authors also observe that cell cycle progression defects and senescence of STAG null cells are dependent on p53 function and that Smc3 acetylation, an essential event for sister chromatid entrapment occuring during replication, is defective in these lines. Lastly, a synthetic effect on survival is observed when STAG2 is inactivated in combination with genes involved in the repair of DNA breaks and sensitivity of STAG2 mutant cells to treatment with chemotherapeutic agents inducing DNA damage or involved in DNA repair is reported.

Based on this evidence, the authors propose a role for Stag2 in promoting replication fork progression and underline the opportunity to exploit this phenotype for the treatment of cancers related to cohesin mutations. In recent years, the involvement of cohesin in controlling replication fork dynamics has emerged. The present report reveals an important aspect of Stag2 involvement in this function, also providing insight on the potential exploitation of cohesin defects for cancer treatment. Hence, this work is of potential interest for researchers working in chromosome dynamics, DNA replication and cancer biology fields. The manuscript is well-written and the results are in general clear. However, there are some shortcomings in the consideration of the background of cohesin functions at replications forks and the interaction of cohesin subunits with replication factors that would need to be addressed before publication in Nature Communications.

Specific comments:

- Relevant previous literature on cohesin recruitment to replication sites and functions in replication fork progression/stability (e.g. Tittle-Elmer et al. 2012, Ribeyre et al. 2016, Frattini et al. 2017, Carvajal-Maldonado et al. 2018) is not considered in the manuscript. These important background references should be mentioned in the introduction and taken into consideration when discussing the manuscript findings.

- The interpretation of the experiments analysing interactions between replication proteins and cohesin subunits is confusing. Authors consider MCM3 and MCM5 as members of the pre-replication complex, but they also form part of the CMG replicative helicase that is the core component of active replication forks. In addition, members of the replication machinery analysed (PCNA and Pol delta) are also involved in different DNA repair pathways. Hence, the conclusion that in STAG2 null cells cohesin interacts less with replication machineries is unclear, as for instance a reduced interaction between SMC3 and MCM3 and MCM5 would be expected. Authors should repeat these experiments analysing factors univocally associated to replication forks (such as Pol epsilon subunits, CDC45 or AND1) to clarify this point. In addition, Co-IP experiments should be performed in cells not traversing S-phase (arrested at the G1/S transition or in mitosis) to define if these effects are S-phase specific (for instance interaction with pre-replication complexes at DNA should only happen in G1).

- On these lines, the representation of replication factors in the model is misleading, as PCNA and DNA pol delta are represented in front of the MCM complex which associates to the leading strand template. These two proteins associate to carry out lagging strand synthesis on the ssDNA template provided by helicase unwinding. In addition, leading and lagging strand distribution should be inverted at each side of the replication origin. The manuscript would benefit from a more careful description of replication factors and their interactions with cohesin (a recent review by Villa-Hernandez et al. might be helpful in this task).

- Authors state in the abstract that fork stalling and collapse upon STAG2 inactivation is “due to disruption of interaction between the cohesin ring and the replication machinery along with failure to stablish SMC3 acetylation”. Besides the interactions between cohesin and fork factors needing clarification, the relationship between fork defects, cohesin-replication factors interaction and SMC3 acetylation is merely correlative. The conclusion that fork defects are due to the other two is not supported by data and should not be stated as such in the manuscript.

We thank the reviewers for their careful consideration of our manuscript and thoughtful suggestions for improvement. Please find below a detailed point-by-point response to each of the concerns and suggested modifications that were made. Incorporating these additional data and revisions has resulted in a substantially improved manuscript that we now believe is suitable for publication in *Nature Communications*.

Reviewer #1

1. My concern is with the non-transformed STAG2 depleted cell lines used throughout the paper. The STAG2 depleted cells shown in Fig. 1D and E have a strong S phase arrest, limited growth, and B-gal positive staining, taken as an indicator of senescence. What day of growth are these cells at? The growth defect seems so severe, it's hard to imagine how these cells can be passaged for 60 Days as shown in Fig. 1C and how they can provide enough material for the experiments shown? These cells look like they are dying. It would be important to know at what day of growth each experiment is done and how these cells come out of S phase arrest to cycle.

STAG2 knockout by CRISPR cleavage or shRNA depletion induces an intra-S-phase cell cycle arrest leading to cellular senescence. These cells stop dividing, acquire morphologic features of senescence, and display beta-galactosidase activity that is a classic marker for cellular senescence (Figure 1E-F). The intra-S-phase cell cycle arrest can be observed by flow cytometry starting as early as 48 hours after shRNA lentiviral transduction with both pLKO and pGIPZ vector systems (Supplementary Figure 1B and 1H). The decreased cellular proliferation is apparent as early as 48 hours after shRNA lentiviral transduction (Supplementary Figure 1C-E and G). Morphologic features of senescence become apparent approximately 5-8 days after shRNA lentiviral transduction with both pLKO and pGIPZ vector systems (Supplementary Figure 1C-E and G). The cells remain arrested within S-phase over an extended period of time (15 days after shRNA lentiviral transduction shown in Supplementary Figure 1B). The cells remain senescent and viable over an extended period of time (morphology through 29 days after shRNA lentiviral transduction shown in Supplemental Figure 1C-E). Western blot of lysate harvested from the senescent cells at 8 days, 35 days, and 60 days after shRNA lentiviral transduction confirms that there is stable knockdown of greater than 99% of STAG2 protein (Figure 1C). We have carefully annotated in the results, figures, and/or figure legends the time points at which the data were collected.

2. These cells are described as senescent. Usually senescence is a DNA damage induced G1 arrest. These cells may be stressed and inducing a DNA damage response, but it is not clear what the B-gal stain signifies. Can the authors provide an explanation?

Beta-galactosidase is a classic marker for cellular senescence. Cell cycle exit and senescence can occur upon DNA damage in any phase of the cell cycle. Upon *STAG2* inactivation, we show that this cell cycle arrest is occurring within S-phase and leads to a senescence phenotype both morphologically and with associated beta-galactosidase activity.

3. For the IPs in Figure 2F, it seems that all proteins tested co-IP with SMC3. It is possible that IP with SMC3 brings down anything that is associated with DNA. The only protein that does not co-IP is ORC1, but since the blot is blank it is not clear that the antibody is even working (and it is also not shown in the input blots in Fig. S2 H). To confirm that these factors co-IP with SMC3 and the cohesin ring through protein interaction and not DNA, the IPs should be done in the presence of ethidium bromide. This would rule out that the IPs are simply bringing down all proteins associated with stalled forks.

We agree with the reviewer that it is important to evaluate whether the interaction between the cohesin complex and replication factors is dependent on chromatin or fork stalling. We have performed multiple additional experiments to address this issue that are presented in the revised manuscript. First, we show in Figure 3A that the interaction of cohesin with the pre-replication complex and the replication machinery occurs across multiple cell lines cultured asynchronously in the absence of replication stress. The interaction between cohesin and the pre-replication complex and the replication machinery is specific, as immunoprecipitation fails to pull down components of the origin recognition complex, ORC1 and ORC3. Whole cell lysates demonstrating the presence of ORC1 and ORC3 proteins in the input lysate for these immunoprecipitation reactions is now presented in Supplementary Figure 3A of the revised manuscript. Next, in order to study the cell cycle specificity of the interaction between cohesin and replication factors, we performed immunoprecipitation using SMC3 antibodies on cells growing asynchronously (+ DMSO vehicle), arrested in G1 phase of the cell cycle (+ palbociclib), arrested in S-phase (+ aphidicolin), or arrested in mitosis (+ colcemid). These results shown in Figure 3D demonstrate that the interaction between cohesin and the replication factors is enriched during S-phase arrest, is absent during mitotic arrest, and is diminished during G1 arrest. Next, to demonstrate if the cohesin interaction with replication factors is chromatin dependent, we performed immunoprecipitation with SMC3 antibodies in the presence of either DNase or MNase treatment (Figure 3E). These nuclease treatments did not prevent the interaction of SMC3 with multiple components of the replication machinery (e.g. MCM5 and PCNA).

4. It is hard to reconcile why the STAG2-depleted non-transformed cells are so sick. The authors show that knockdown of p53 has a dramatic effect reversing the growth defect in STAG2 depleted cells and suggest that the effect helps to explain co-occurrence of STAG2 and p53 mutation in Ewing sarcoma. However, there are many examples of STAG2 tumors without p53 mutation - how do these cells grow so well?

Yes, it is true as the reviewer notes that while *STAG2* and *TP53* mutations frequently co-occur in Ewing sarcoma, *STAG2* mutations in papillary urothelial carcinoma of the urinary bladder commonly occur in association with *TERT* promoter mutation and *FGFR3* activation via either mutation or fusion in tumors that are often *TP53* wildtype. It is currently unclear how these tumors proliferate with *STAG2* mutation despite absence of identifiable *TP53* alterations, but we speculate that these cancers have otherwise subverted the intra-S-phase cell cycle arrest via other cellular mechanisms. This indicates that while *TP53* inactivation is one mechanism by which cancer cells can subvert the intra-S-phase cell cycle arrest that is caused by STAG2 deficiency, there are likely to be other mechanisms by which this can occur in *TP53* wildtype cancers.

5. The synthetic lethality screen and the subsequent tests of survival using genotoxic agents is very convincing; it is done with four isogenic cell lines and is very reproducible. However, a previous study (Bailey et al, Mol Cancer Ther 2014) showed that STAG2 tumor cells are sensitive to PARP inhibitors and other DNA-damaging agents. In fact, they used the same 2 (of 4) cell lines (42MGBA and H4) used in the current study. While the current study is extensive and well done, the previous work detracts from the novelty.

While this prior study has identified that glioblastoma cells harboring *STAG2* mutational inactivation have increased sensitivity to PARP inhibition, our study for the first time describes a mechanistic basis for this synthetic lethality between *STAG2* mutation and DNA double-strand break repair genes. Furthermore, we demonstrate that this synthetic lethality is not limited to glioblastoma, but also occurs in Ewing sarcoma cells and an epithelial cell line modeling carcinoma as well. Additionally, we

demonstrate that this increased sensitivity extends beyond PARP inhibition, and also includes ATR inhibition and cytotoxic chemotherapeutic agents whose mechanism is induction of DNA double-strand breaks.

Reviewer #2

Major points:

1. shRNAs, in particular pLKO based constructs, are notorious for off-target effects and have often led to wrong conclusions. The lack of experiments demonstrating decisively on-target effect of perturbations is worrying. This could be rectified by expression of shRNA-insensitive transgenes or engineering of shRNA target sites in the genome. These experiments should be conducted for STAG2 in one or two key experiments in replication progression. Similarly, rescue experiments should be conducted for at least some candidates (e.g: ATR, BRCA1, RAD51, XRCC5 and PRKDC) in the viability setting.

We understand the reviewer's concern regarding possible off-target effects of the experiments performed in our manuscript using lentiviral shRNA depletion. However, in all instances throughout the manuscript, we have used two independent shRNA sequences against *STAG2*, the other cohesin subunits, and the DNA repair genes selected for study. Performing experiments using two independent shRNA sequences has been the accepted standard for scientific publication in reputable journals such as *Nature*, *Science*, and *Cell* over the past decade. Additionally, we note that the same cellular phenotype was observed for the two independent shRNA sequences across multiple cell lines (RPE, BJ, and SVG p12 cells for the cohesin shRNA knockdown experiments; H4, 42MGBA, TC-106, and RPE cells for the DNA repair gene shRNA knockdown experiments), providing further credibility for the results. Furthermore, for the DNA repair gene shRNA synthetic lethality screen, these results were consistent with small molecule inhibitors targeting the encoded proteins (both ATR and PARP), providing further credibility for the results. For the replication arrest and senescence phenotype observed after lentiviral shRNA knockdown of *STAG2*, this was also observed after CRISPR-mediated inactivation of *STAG2* in both RPE and BJ cells, providing further credibility for the results.

Nonetheless, in order to provide further evidence that *STAG2* knockdown and not off-target effects are responsible for the intra-S-phase cell cycle arrest and cellular senescence we observed after CRISPR knockout or shRNA knockdown using the pLKO vector system, we have now performed shRNA depletion of *STAG2* using the pGIPZ lentiviral system using three independent shRNA sequences that are distinct from those in the pLKO knockdown experiments. As shown in Supplemental Figure 1F, G, and H in the revised manuscript, all three of these pGIPZ shRNA's directed against *STAG2* caused >99% reduction in STAG2 protein, induced an identical intra-S-phase cell cycle arrest, and morphologic features of senescence, which were not observed after lentiviral transduction with empty pGIPZ control vector.

2. The very strong selective effect of ATR, BRCA1, RAD51, XRCC5 and PRKDC in STAG2 ko vs STAG2 wild-type cells is quite striking (Figure 5A-E and suppl Figs). In addition to the on/off-target issue (see above), these experiments lack key controls: what is the effect of the depletion of pan-essential genes across the cells used? Without these data and demonstrating the impact of gene depletions of pan essential genes in the wild-type setting, it is hard to conclude selective effects in mutants.

We have now performed experiments examining the effects of *SMC3*, *SMC1A*, *RAD21*, and *STAG1* depletion in *STAG2* +/- isogenic glioblastoma, Ewing sarcoma, and RPE cells. We show in Figure 6 of the revised manuscript that *SMC3*, *SMC1A*, and *RAD21* are required genes in all four isogenic pairs, with cell death uniformly observed in both STAG2 proficient and deficient cells. In contrast, *STAG1* depletion had

no significant growth inhibition on STAG2 proficient cells, whereas *STAG1* depletion uniformly resulted in cell death across all four of the STAG2 deficient cells.

3. The recent study by Liu et al., did not detect enhanced sensitivity of STAG2 mutated cells to cisplatin or PARP inhibitors without depleting STAG1 in addition. How can this be reconciled?

The study by Liu et al did not perform any experiments in *STAG2* +/- isogenic cell systems to test the response to PARP inhibitors or DNA damaging chemotherapeutic agents. They only took *STAG2* mutant cancer cell lines and assessed for differential sensitivity to PARP inhibitors +/- *STAG1* shRNA knockdown, where they found that *STAG1* knockdown caused increased sensitivity. Our data in multiple isogenic *STAG2* +/- cell lines from different tumor types demonstrates that STAG2 deficiency alone causes significantly enhanced sensitivity to PARP inhibition, ATR inhibition, and other DNA damaging chemotherapeutic agents. As shown in Figure 6 of our revised manuscript, our newly added results indicate that lentiviral shRNA depletion of *STAG1* in *STAG2* mutant cancer cell lines results in cell death, which is independent of adding a PARP inhibitor or DNA damaging agent. This is a similar result to the recent published studies by Benedetti et al (*Oncotarget* 2017) and van der Lelij et al (*Elife* 2017). It is likely that incomplete knockdown of *STAG1* in the study by Liu et al (see their supplemental figures showing only partial *STAG1* depletion) did not result in complete cell death, resulting in a viable cell population that they then treated with PARP inhibitors and cisplatin. This is in contrast to the >99% depletion of *STAG1* we obtained in our *STAG2* mutant cells (Supplemental Figure 1I of our revised manuscript), where we saw complete cell death independent of adding a PARP inhibitor or DNA damaging agent (Figure 6 of our revised manuscript).

Benedetti L, Cereda M, Monteverde L, Desai N, Ciccarelli FD. Synthetic lethal interaction between the tumour suppressor STAG2 and its paralog STAG1. *Oncotarget* 2017 Jun 6;8(23):37619-37632. PMID: 28430577

van der Lelij P, Lieb S, Jude J, Wutz G, Santos CP, Falkenberg K, Schlattl A, Ban J, Schwentner R, Hoffmann T, Kovar H, Real FX, Waldman T, Pearson MA, Kraut N, Peters JM, Zuber J, Petronczki M. Synthetic lethality between the cohesin subunits STAG1 and STAG2 in diverse cancer contexts. *Elife* 2017 Jul 10;6. pii: e26980. PMID: 28691904

4. The co-immunoprecipitation of cohesin with DNA replication factors in Figure 2F is not well controlled and described. Using unspecific antibodies as controls is not sufficient. Using cells stably expressing tagged cohesin subunits and untagged cells as controls would improve the experiment and impact of the data. What is the relative IP and CoIP efficiency of the individual proteins? Showing lysate, flow through and precipitated fractions would help here. Is the interaction indirectly mediated by DNA (nuclease digestion)?

In order to address this reviewer's concern, we have performed several additional experiments to confirm and extend our identification of interaction between cohesin and the replication machinery. First, as recommended by Reviewer #4, we have now assessed and demonstrate interaction of cohesin with CDC45, AND1, and PolE, all of which are factors unequivocally present at replication forks (see Figure 3A of the revised manuscript). This interaction of cohesin with the pre-replication complex and the replication machinery occurs across multiple cell lines cultured asynchronously in the absence of replication stress. The interaction between cohesin and the pre-replication complex and the replication machinery is specific, as immunoprecipitation fails to pull down components of the origin recognition complex, ORC1 and ORC3 (Figure 3A). Whole cell lysates demonstrating the presence of ORC1 and ORC3

proteins in the input lysate for these immunoprecipitation reactions is presented in Supplementary Figure 3A. Additionally, we have studied the cell cycle specificity of the interaction between cohesin and replication factors in RPE cells growing asynchronously (+ DMSO vehicle), arrested in G1 phase of the cell cycle (+ palbociclib), arrested in S-phase (+ aphidicolin), or arrested in mitosis (+ colcemid). These results shown in Figure 3D of the revised manuscript demonstrate that the interaction between cohesin and replication factors is enriched during S-phase arrest, is absent during mitotic arrest, and is diminished or absent during G1 arrest. Next, to demonstrate if the cohesin interaction with replication factors is chromatin dependent, we performed immunoprecipitation with SMC3 antibodies in the presence of either DNase or MNase treatment (Figure 3E of the revised manuscript). These nuclease treatments did not prevent the interaction of SMC3 with multiple components of the replication machinery (e.g. MCM5 and PCNA).

The immunoprecipitation reactions demonstrating interaction between cohesin and replication factors were performed using well characterized antibodies that have been used in several prior studies. We demonstrate co-immunoprecipitation of MCM3 with each of the core cohesin complex subunits (e.g. SMC3, SMC1A, RAD21, and STAG2) in Figure 3B, Figure 3C, and Supplemental Figure 3B of the revised manuscript. The cohesin regulatory factor PDS5A also co-immunoprecipitated with MCM3, but the cohesin loading factor NIPBL did not. We did not detect interaction between Securin or MAD2 with MCM3 (Figure 3C and Supplemental Figure 3B of the revised manuscript). We believe that our results demonstrating this interaction of the endogenous cohesin proteins with endogenous replication factor proteins across multiple human cell lines has produced definitive, unquestionable results. We do not believe there is a need to exogenously overexpress epitope-tagged versions of the cohesin or replication proteins in order to confirm this interaction that we definitively demonstrate to exist using well characterized antibodies against the endogenous proteins.

5. If S phase arrest and fork instability in non-transformed that lack STAG2 is dependent on TP53, this would suggest that STAG2 does not play a direct role in fork stability but rather that lack of STAG2 in non-transformed cells causes genome instability events that require TP53 action for impact. How does this relate to the described binding of STAG2 to replication factors and is the title of the paper still accurate? Across TCGA datasets, is there a significant association of deleterious STAG2 mutations with TP53 alterations?

We have now performed experiments examining the mechanism by which *TP53* inactivation allows bypass of the intra-S-phase arrest in *STAG2* mutant cells. As shown in Figure 5H of the revised manuscript, we demonstrate that *TP53* inactivation in the context of *STAG2* knockdown in RPE cells enables bypass of the intra-S-phase arrest by restoring the interaction of cohesin with the replication factors PCNA, PolE, and PolD, as well as blocking the enhanced binding of cohesin with MCM3, MCM5, CDT1, and Geminin. However, *TP53* inactivation did not block the enhanced interaction of cohesin with RPAp34 or CDC6. Thus, while p53 is a critical regulator of replication fork procession in *STAG2* mutant cells, there are perturbations of the interaction between cohesin and replication factors that are independent of p53 control (e.g. RPAp34 and CDC6). We present these new results and discuss their implications in the revised manuscript.

Regarding the association of *STAG2* mutation and *TP53* mutations in human cancers, there are four major cancer types in which *STAG2* mutations are frequent: glioblastoma, Ewing sarcoma, urothelial carcinoma, and myeloid leukemia. While *STAG2* and *TP53* mutations frequently co-occur in Ewing sarcoma, *STAG2* mutations in papillary urothelial carcinoma of the urinary bladder commonly occur in association with *TERT* promoter mutation and *FGFR3* mutation/fusion in tumors that are often *TP53*

wildtype. There also is not a strong association of cohesin mutations and *TP53* inactivation in myeloid leukemia. It is currently unclear how these tumors proliferate with *STAG2* mutation despite absence of identifiable *TP53* alterations, but we speculate that these cancers have otherwise subverted the intra-S-phase cell cycle arrest via other cellular mechanisms. This indicates that while *TP53* inactivation is one mechanism by which cancer cells can subvert the intra-S-phase cell cycle arrest that is caused by STAG2 deficiency, there are likely to be other mechanisms by which this can occur in *TP53* wildtype cancers.

Minor points:

1. Abstract: "… STAG2 is essential for DNA replication fork progression …": this statement should be altered as STAG2 is only required for "normal" fork progression and that only in the absence of TP53.

We agree that this sentence requires modification. We have now revised this sentence to state, "Here we show that *STAG2* is essential for DNA replication fork progression <u>in non-transformed cells</u>, whereby *STAG2* inactivation leads to…".

2. Page 3 intro: "… no direct DNA binding motifs within the core cohesin subunits …"; this statement should be modified as a recent paper has identified a DNA binding site in cohesin (PMID: 30109982).

We thank the reviewer for pointing out this new study. While this study structurally elucidates the interaction of cohesin with chromatin, there remains no nucleotide sequence specificity to this interaction that was identified, in contrast to how transcription factors recognize specific DNA motifs. In order to clarify our point about how DNA binding regulatory proteins such as CTCF are responsible for positioning cohesin along the genome, we have modified this sentence in the revised manuscript to the following, "While cohesin forms a ring-like structure that encircles chromatin, <u>no DNA binding motifs with nucleotide sequence specificity</u> have been identified within the core cohesin subunits. However, emerging studies have shown that cohesin is enriched at specific chromatin loci including active transcriptional sites and pericentric heterochromatin, suggesting cohesin localization is directed by specific DNA-binding regulatory proteins. The CCCTC-binding factor (CTCF) has been identified as a direct binding partner of STAG2 that is dispensable for cohesin loading onto chromatin but is required for cohesin enrichment at specific enhancer regulatory loci throughout the genome."

3. Figure 2D: RPAp34 foci should be quantified

These data have been quantified as requested with results shown in Figure 2E of the revised manuscript.

4. Figure S6F: H4 STAG2 KI cells are more strongly affected by cyclophosphamide than H4 parental cells. Is this a wrong labeling?

We thank the reviewer for noticing this labeling error in Supplemental Figure 6F, which has now been corrected in the revised manuscript (is now Supplemental Figure 8F in the revised manuscript). We have carefully checked that all figures are correctly labeled in the revised manuscript, as well as have improved the labeling in select panels to improve clarity for the readers (e.g. now says "H4 parental (*STAG2* mutant)" throughout instead of just "H4 parental").

5. The role of the lack of SMC3 lysine acetylation in STAG2 mutant cells is unclear given that H4 cells lack STAG2 but show SMC3 lysine acetylation.

Following ablation of STAG2 in RPE cells via shRNA depletion or TC-106 Ewing sarcoma cells via CRISPR recombination, we observe a complete loss of SMC3 lysine acetylation (Figure 3G and Figure 5J). However, in H4 glioblastoma cells with STAG2 mutation, they have significantly reduced (but not absent) quantity of SMC3 lysine acetylation compared to their isogenic counterpart after reconstitution of wildtype STAG2 by homologous recombination.

6. Supplementary figures are spread over multiple pages

Yes, this is correct. We have chosen to display our supplemental data in 8 supplemental figures, with each supplemental figure showing data that is relevant to the corresponding figure in the main manuscript (e.g. Supplemental Figure 1 contains all the relevant supplemental data for Figure 1, Supplementary Figure 2 contains all the relevant supplemental data for Figure 2, etc.).

7. Given the different origin of cell lines (glioblastoma, RPE cells) used the dose-response experiments in Figure S6, the consistent curve shapes obtained for many drugs across the different cell models is remarkable and surprising. Drug response profiles for a given drug across models are usually impacted by drug exporter activities, genetic background alterations present and the metabolic parameters of cell lines and rarely that consistent.

We agree that the consistency with which select cytotoxic chemotherapeutic agents as well as ATR and PARP inhibitors cause the selective death of *STAG2* mutant but not *STAG2* widltype cells across the four sets of isogenic glioblastoma, Ewing sarcoma, and RPE cells is quite striking and remarkable. We look forward to working cooperatively with oncologists to translate this selective vulnerability of *STAG2* mutant cancer cells into clinical trials with these targeted agents for affected patients.

Reviewer #3

Abstract and Introduction:

In the abstract, the statement "therapeutically targeting cohesin mutant cancer cells are unknown" could be rephrased to largely unknown.

We agree and have modified this sentence as suggested in the abstract of the revised manuscript.

It would also be good, in my opinion, to mention that the work in this study give some explanations to the counterintuitive finding that STAG2 is essential for replication fork progression and at the same time inactivating mutations in STAG2 are often found in various cancers, that are in general fast proliferating.

We agree and have added text to the discussion section of the revised manuscript to address this point.

In several places, all through the manuscript, the language needs to be carefully edited. Some sentences are extremely long and complicated, leaving many readers lost if they are at all unfamiliar with the subject. For example, the sentence in the introduction starting on Line 36, with "The cohesion subunits form,… and ends on Line 42!

We apologize for the complexity of some sentences in the initial submission and have split up a few of the longer sentences into smaller and more easily comprehensible sentences as suggested in the revised manuscript.

Line 37, A minor comment; in mammalian cells cohesin is loaded already in telophase.

We agree with the reviewer. We have updated this sentence in the revised manuscript as follows, "which is loaded onto chromatin in early G1 phase of the cell cycle immediately following cytokinesis".

Line 66, I believe that Liu J et al senior author JD Krantz showed conserved transcriptional dysregulation of gene expression in CdLS patients already 2009 (PMID: 19468298) which could be a better reference here.

We agree and have added this reference here as suggested in the revised manuscript.

Line 72, should be: in four or more

Yes, this should state "in four or more". This has been corrected in the revised manuscript.

Results:
In general, all through the manuscript, the authors claim that they are using primary human cells. The RPE cells is likely the best cell culture model for studies like this, but are they really non-transformed? They are immortilized with the hTERT. An active telomerase is what characterize many Cancer cells. Therefore, I suggest to be less strong about the statement that non-transformed primary cells are used.

RPE and BJ cells are hTERT immortalized primary human retinal pigmented epithelial cells and foreskin fibroblasts. While these cells have been immortalized by overexpression of hTERT so that they can grow indefinitely in cell culture, they are both non-transformed as they lack activation of oncogenes (e.g. Ras) and inactivation of tumor suppressor genes (e.g. p53, Rb) required for transformation. Both of these RPE and BJ cells do not form colonies in soft agar, nor do they form tumors when subcutaneously injected into immunodeficient mice, which are the two classic markers of cellular transformation.

Line 103, Another major concern is the fact that STAG2 is claimed to be a required gene. Regardless of this, experiments are done on cells that have been without STAG2 for at least 8 days after shRNA treatment? As can be judged from the Western blot the knock down is also extremely efficient. How many things are not happening in these cells during the 8 days. How many cell divisions would the cells go through normally and what is the genomic characteristics after this time in the absence of STAG2? This should at least be discussed or somehow tested? How soon is the senescent stage or proliferative arrest appearing? How can the cells be amplified for collection of genomic DNA for sequencing if they are not dividing?

In order to clarify these questions, we have added several additional supplementary data to Supplemental Figure 1 in the revised manuscript and have also carefully annotated in the results, figures, and/or figure legends the time points at which the data were collected. *STAG2* knockout by CRISPR cleavage or shRNA depletion induces an intra-S-phase cell cycle arrest leading to cellular senescence. The intra-S-phase cell cycle arrest can be observed by flow cytometry starting as early as 48 hours after shRNA lentiviral transduction with both pLKO and pGIPZ vector systems (Supplementary Figure 1B and 1H). The decreased cellular proliferation is apparent as early as 48 hours after shRNA lentiviral transduction for RPE, BJ, and SVG p12 cells (Supplementary Figure 1C-E and G). Morphologic features of senescence become apparent approximately 5-8 days after shRNA lentiviral transduction with both pLKO and pGIPZ vector systems (Supplementary Figure 1C-E and G). The cells remain arrested within S-phase over an extended period of time (15 days after shRNA lentiviral transduction shown in

Supplementary Figure 1B). The cells remain senescent and viable over an extended period of time (morphology through 29 days after shRNA lentiviral transduction shown in Supplemental Figure 1C-E). Western blot of lysate harvested from the senescent cells at 8 days, 35 days, and 60 days after shRNA lentiviral transduction confirms that there is stable knockdown of greater than 99% of STAG2 protein (Figure 1C).

Line 107 and onwards, it is claimed that STAG2 is inactivated by shRNA in three cell lines. Results are shown only for RPE cells, where are the data from BJ and SVG p12 cells, that are claimed to show the same result? Also, the time point for FACS analysis and microscopy should be indicated (as is stated in M&M).

We now show results for STAG2 shRNA depletion in RPE, BJ, and SVG p12 cells in the revised manuscript (Supplementary Figure 1C-E). These data demonstrate decreased cellular proliferation that is apparent as early as 48 hours after shRNA lentiviral transduction across all three cell lines, and that all three cell lines remain senescent and viable over an extended period of time (morphology through 29 days after shRNA lentiviral transduction shown. As requested, we have also carefully annotated in the results, figures, and/or figure legends the time points at which the data were collected throughout the revised manuscript.

Line 132 and onwards, give a reference for the accumulation of RPAp34 on stalled replication forks

As requested, we have added a reference here for the prior study which demonstrated that GFP-tagged RPAp34 does not visibly accumulate at replication sites under normal S-phase conditions, but focally accumulates at stalled replication forks induced by the DNA polymerase inhibitor aphidicolin (Solomon et al *J Cell Biol* 2004).

Solomon DA, Cardoso MC, Knudsen ES. Dynamic targeting of the replication machinery to sites of DNA damage. *J Cell Biol* 2004 Aug 16;166(4):455-63. PMID: 15314062

Line 157, potentially a weak MCM3 band is actually seen in the NIPBL IP Blot Figure S2, in RPE cells, comment?

Upon longer exposure of this Western blot following immunoprecipitation using NIPBL antibodies, we do not see evidence of a definitive band for MCM3.

Line 160, I would say that the interaction between SMC3 and PolD is reduced not disrupted.

We agree and have changed this to reduced in the revised manuscript. In response to suggestions by Reviewer #4, please note that we have also studied the interaction between SMC3 and PolE. We see an even greater reduction in interaction for PolE following lentiviral shRNA depletion of STAG2.

Line 175 and onwards, indicate which cell line is used for the G1 arrest exp. Also show a FACS profile for the degree of G1 arrest and S phase accumulation after release from CDK4/6 inhibitor.

This experiment was performed in U87MG human glioma cells as described in the Methods and the experimental schematic in Supplemental Figure 4A of the revised manuscript, although similar results were also found in RPE cells (not shown). FACS profiles of RPE cells arrested in G1 phase of the cell cycle

using the CDK4/6 inhibitor palbociclib is shown in both Supplemental Figure 1A and Supplemental Figure 5B in the revised manuscript.

Line 179-180, why is it important to say that STAG2 depletion in multiple non-transformed human cell lines resulted in ….? When results are shown from two potentially not so non-transformed cell lines?

The 53BP1 foci accumulation and activation of DNA damage checkpoint signaling following *STAG2* lentiviral shRNA depletion was observed in three hTERT-immortalized, non-transformed human cell lines (RPE, BJ, and SVG p12). As previously stated, these cells are non-transformed as they do not form colonies in soft agar or tumors in immunodeficient mice. We chose to specify that these cellular phenotypes were observed specifically in non-transformed human cells, as potentially different outcomes would be expected in transformed human cancer cells. Indeed, *STAG2* depletion or inactivation in human cancer cells with *TP53* inactivation does not cause an intra-S-phase arrest, activation of checkpoint signaling, and induction of cellular senescence.

Figure 3D, Probably more than one blot needed to be run to detect all these proteins? Could the same extract be used for all? How can otherwise one loading control be used?

All of the Western blots shown in Figure 3D of the initial submission (Figure 4D of the revised manuscript) are from lysate collected from RPE and SVG p12 cells following lentiviral infection with either empty pLKO.1 vector, two independent *STAG2* shRNAs, or mock infected control. A single set of lysates were used for all of these Western blots. The loading control used for these Western blots is beta-actin, with additional blots for total ATR and total BRCA1 protein levels also shown, indicating that a very similar quantity of protein is present for each of the four lanes.

Line 262 and onwards plus Fig S6F, is the labelling of the plate reversed from the graph H4 STAG2 KI seems to be surviving better (forming more colonies).

We thank the reviewer for noticing this labeling error in Supplemental Figure 6F, which has now been corrected in the revised manuscript (is now Supplemental Figure 8F in the revised manuscript). We have carefully checked that all figures are correctly labeled in the revised manuscript, as well as have improved the labeling in select panels to improve clarity for the readers (e.g. now says "H4 parental (*STAG2* mutant)" throughout instead of just "H4 parental").

Discussion:
The synthetic lethality between KO of SA1 in combination with SA2 could be discussed.

We have now performed experiments examining the effects of *SMC3*, *SMC1A*, *RAD21*, and *STAG1* depletion in *STAG2* +/- isogenic glioblastoma, Ewing sarcoma, and RPE cells. We show in Figure 6 of the revised manuscript that *SMC3*, *SMC1A*, and *RAD21* are required genes in all four isogenic pairs, with cell death uniformly observed in both STAG2 proficient and deficient cells. In contrast, *STAG1* depletion had no significant growth inhibition on STAG2 proficient cells, whereas *STAG1* depletion uniformly resulted in cell death across all four of the STAG2 deficient cells.

What is the proposed mechanism for TP53 in activation of the intra-S-phase arrest – discuss or suggest.

We have now performed experiments examining the mechanism by which *TP53* inactivation allows bypass of the intra-S-phase arrest in *STAG2* mutant cells. As shown in Figure 5H of the revised

manuscript, we demonstrate that *TP53* inactivation in the context of *STAG2* knockdown in RPE cells enables bypass of the intra-S-phase arrest by restoring the interaction of cohesin with the replication factors PCNA, PolE, and PolD, as well as blocking the enhanced binding of cohesin with MCM3, MCM5, CDT1, and Geminin. However, *TP53* inactivation did not block the enhanced interaction of cohesin with RPAp34 or CDC6. Thus, while p53 is a critical regulator of replication fork procession in *STAG2* mutant cells, there are perturbations of the interaction between cohesin and replication factors that are independent of p53 control (e.g. RPAp34 and CDC6). We present these new results and discuss their implications in the revised manuscript.

Reviewer #4

1. Relevant previous literature on cohesin recruitment to replication sites and functions in replication fork progression/stability (e.g. Tittle-Elmer et al. 2012, Ribeyre et al. 2016, Frattini et al. 2017, Carvajal-Maldonado et al. 2018) is not considered in the manuscript. These important background references should be mentioned in the introduction and taken into consideration when discussing the manuscript findings.

We thank this reviewer for his/her in-depth knowledge of the DNA replication process, especially as it relates to the emerging role of cohesin in regulating replication fork stability. We have added additional text to the revised manuscript discussing these prior studies as appropriate.

2. The interpretation of the experiments analysing interactions between replication proteins and cohesin subunits is confusing. Authors consider MCM3 and MCM5 as members of the pre-replication complex, but they also form part of the CMG replicative helicase that is the core component of active replication forks. In addition, members of the replication machinery analysed (PCNA and Pol delta) are also involved in different DNA repair pathways. Hence, the conclusion that in STAG2 null cells cohesin interacts less with replication machineries is unclear, as for instance a reduced interaction between SMC3 and MCM3 and MCM5 would be expected. Authors should repeat these experiments analysing factors univocally associated to replication forks (such as Pol epsilon subunits, CDC45 or AND1) to clarify this point. In addition, Co-IP experiments should be performed in cells not traversing S-phase (arrested at the G1/S transition or in mitosis) to define if these effects are S-phase specific (for instance interaction with pre-replication complexes at DNA should only happen in G1).

We thank this expert reviewer for his/her informative comments about DNA replication and our mechanistic investigation into the role of cohesin in this process. Per the reviewer's suggestion, we have now assessed the interaction of cohesin with CDC45, AND1, and PolE (Figure 3A of the revised manuscript), as well as analyzed the interaction of cohesin with these replication factors in the presence of *STAG2* depletion (Figure 3F of the revised manuscript). Additionally, we have studied the cell cycle specificity of the interaction between cohesin and replication factors as recommended by immunoprecipitation using SMC3 antibodies on RPE cells growing asynchronously (+ DMSO vehicle), arrested in G1 phase of the cell cycle (+ palbociclib), arrested in S-phase (+ aphidicolin), or arrested in mitosis (+ colcemid). These results shown in Figure 3D of the revised manuscript demonstrate that the interaction between cohesin and the replication factors is enriched during S-phase arrest, is absent during mitotic arrest, and is diminished or absent during G1 arrest.

3. On these lines, the representation of replication factors in the model is misleading, as PCNA and DNA pol delta are represented in front of the MCM complex which associates to the leading strand template. These two proteins associate to carry out lagging strand synthesis on the ssDNA template provided by

helicase unwinding. In addition, leading and lagging strand distribution should be inverted at each side of the replication origin. The manuscript would benefit from a more careful description of replication factors and their interactions with cohesin (a recent review by Villa-Hernandez et al. might be helpful in this task).

We thank the reviewer for his/her informative comments about the model that we generated for this manuscript. We have extensively modified this model that is now Figure 9 of the revised manuscript in accordance with the suggestions of this expert reviewer.

4. Authors state in the abstract that fork stalling and collapse upon STAG2 inactivation is "due to disruption of interaction between the cohesin ring and the replication machinery along with failure to stablish SMC3 acetylation". Besides the interactions between cohesin and fork factors needing clarification, the relationship between fork defects, cohesin-replication factors interaction and SMC3 acetylation is merely correlative. The conclusion that fork defects are due to the other two is not supported by data and should not be stated as such in the manuscript.

We agree that the replication fork stalling is almost certainly not caused by the failure to establish SMC3 acetylation, as concurrent *STAG2* and *TP53* inactivation allows DNA replication to proceed despite failure to establish SMC3 acetylation (Figure 5I and J). We do speculate that the replication fork defect observed in *STAG2* mutant cells is very likely to be directly caused by disruption of the interaction between cohesin and replication factors. However, given that our current data do not unequivocally prove a causal relationship, we have revised the text in the manuscript accordingly. For instance, the referenced sentence in the abstract has been modified in the revised manuscript to, "whereby STAG2 inactivation leads to replication fork stalling and collapse <u>with</u> disruption of interaction between the cohesin ring and the replication machinery as well as failure to establish SMC3 acetylation."

Reviewers' comments:

Reviewer #1 (Remarks to the Author):

The authors have satisfactorily addressed all my points raised in the previous round of review. I feel the manuscript is now acceptable for publication in Nature Communications.

Reviewer #2 (Remarks to the Author):

The authors have conducted a series of additional experiments to address the concerns of the referees. In particular the additional control depletion experiments with shRNAs targeting pan-essential genes were an important addition for interpreting the differential effects of DNA damage gene depletion in isogenic models.

While the authors did not conduct transgenic rescue experiments for shRNA depletion effects, I agree with the argument authors that the effects shown with multiple shRNAs and gRNAs are very likely on target.

Following the added data and text changes in the revised version of the MS, the paper is suitable for publication in my opionion.

Reviewer #3 (Remarks to the Author):

In this revised version of the manuscript the authors have, together with the point by point rebuttal, answered the questions I had to the initial submission. I am thus content with this version of the manuscript.

Reviewer #4 (Remarks to the Author):

The revised manuscript by Mondal et al. includes some text changes and experiments aimed at addressing the points raised by this reviewer regarding the role of cohesin in promoting fork progression and the impact of STAG2 silencing of the interaction of cohesin with replisome factors. The changes provided however fall short to substantiate the main conclusion that STAG2 is required for interaction of cohesin with active replication machineries. In addition, the context of cohesin functions during DNA replication is still not appropriately considered and discussed.

- Authors performed experiments addressing the interaction between cohesin and replication factors in different cell cycle stages that are convincing. They also addressed the effect of the loss of STAG2 in the interaction of SMC3 by co-inmunoprecipitation with additional replication proteins (i.e CDC45, AND1 and POLE). However, these new results are contradictory, as in STAG2 interfered cells interaction with PolE is reduced (similarly to what previously observed for PCNA and PolD) while interaction with CDC45 and AND1 is unchanged. In addition, interaction with factors belonging to pre-replication complexes is increased. The interpretation of these results is complex and certainly the evidence presented does not provide clear support to the conclusion that loss of STAG2 disrupts the association of cohesin rings with active replication machineries. The data point at STAG2 silencing both increasing interaction of cohesin with pre-replication complexes (directly or indirectly as more pre-RCs may accumulate as a consequence of origin firing repression due to the intra-S phase arrest)

and reducing association with some replisome components (which may reflect the dissociation of these factors from chromatin following fork collapse).

- Discussion of thedata regarding replication is very confusing in the manuscript. What do authors mean by “uncoupling of the pre-replication complex with DNA replication factors”? What is the rational for cohesin engagement of geminin and disengagement from replisome factors as PCNA, PolD and PolE contributing to fork stalling or collapse? How the obervations that cohesin loses interaction with some replisome factors while retaining association to others are reconciled?

- In this respect, the relevant previous literature providing insight on the function of cohesin proteins in promoting replication fork progression has not been appropriately considered. It would be helpful for lay readers that a background on cohesin function in replication is provided and that the findings presented in this work are discussed in the light of previous insight.

- While the schematic model has been improved, pre-RC factors (CDC6 and CDT1, GEMININ) are placed at replication forks, where they should not be present.

The above points are essential to understand STAG2 function in promoting replication and should be clarified prior to publication of the manuscript.

Reviewer #1
The authors have satisfactorily addressed all my points raised in the previous round of review. I feel the manuscript is now acceptable for publication in Nature Communications.

Reviewer #2
Following the added data and text changes in the revised version of the MS, the paper is suitable for publication in my opinion.

Reviewer #3
In this revised version of the manuscript the authors have, together with the point by point rebuttal, answered the questions I had to the initial submission. I am thus content with this version of the manuscript.

Reviewer #4
1. Authors performed experiments addressing the interaction between cohesin and replication factors in different cell cycle stages that are convincing. They also addressed the effect of the loss of STAG2 in the interaction of SMC3 by co-inmunoprecipitation with additional replication proteins (i.e. CDC45, AND1, and POLE). However, these new results are contradictory, as in STAG2 interfered cells interaction with PolE is reduced (similarly to what previously observed for PCNA and PolD) while interaction with CDC45 and AND1 is unchanged. In addition, interaction with factors belonging to pre-replication complexes is increased. The interpretation of these results is complex and certainly the evidence presented does not provide clear support to the conclusion that loss of STAG2 disrupts the association of cohesin rings with active replication machineries. The data point at STAG2 silencing both increasing interaction of cohesin with pre-replication complexes (directly or indirectly as more pre-RCs may accumulate as a consequence of origin firing repression due to the intra-S phase arrest) and reducing association with some replisome components (which may reflect the dissociation of these factors from chromatin following fork collapse).

We thank this reviewer for his/her expert knowledge of the DNA replication process, especially as it relates to the emerging role of cohesin in regulating replication fork stability. We agree that we have over-simplified the interpretation of our results within the Discussion section of the manuscript. While STAG2 inactivation disrupts binding of cohesin with some components of the active replication machinery (e.g. PCNA, PolD, and PolE), the interaction of cohesin with other subunits of the active replication machinery (e.g. CDC45 and AND1) is not significantly perturbed. In addition, we find a robust increase in the binding of cohesin with the single stranded DNA binding protein RPA2/p34 and the replication licensing factor Geminin following STAG2 depletion; however, we observe only a modest increase in the binding of cohesin with MCM helicase subunits and replication licensing factors CDC6 and CDT1 following STAG2 depletion. These findings indicate that the interaction of the cohesin ring with the pre-replication complex and the active replication machinery appears to be a complex and dynamic process. We have modified the relevant text in the Discussion section of the revised manuscript to account for this complexity that this reviewer astutely pointed out.

2. Discussion of the data regarding replication is very confusing in the manuscript. What do authors mean by "uncoupling of the pre-replication complex with DNA replication factors"? What is the rational for cohesin engagement of geminin and disengagement from replisome factors as PCNA, PolD, and PolE contributing to fork stalling or collapse? How the observations that cohesin loses interaction with some replisome factors while retaining association to others are reconciled?

As discussed in the comment above, we agree with the reviewer that the Discussion section of our manuscript would be improved by an expansion of the text regarding the role of cohesin at the replication fork. We observe a robust increase in the binding of cohesin with the replication licensing factor Geminin following STAG2 depletion, a protein known to be essential for preventing genome reduplication. In contrast, we find a marked reduction in the binding of cohesin with some components of the active replication machinery (e.g. PCNA, PolD, and PolE) but not others (e.g. CDC45 and AND1). We speculate that the enhanced binding of cohesin with Geminin following STAG2 depletion represents a critical mediator of an intra-S-phase checkpoint to ensure establishment of sister chromatid cohesion during DNA replication. The disruption of cohesin binding with the replication factors PCNA, PolD, and PolE upon STAG2 depletion may represent either lack of recruitment to the active replication fork or instability and dissociation from the stalled replication fork. Further mechanistic investigation is required to definitively address these questions. As requested, we have expanded the Discussion section of our revised manuscript to incorporate these points and have modified the statement regarding the "uncoupling of the pre-replication complex with DNA replication factors" that this reviewer found confusing/inaccurate.

3. In this respect, the relevant previous literature providing insight on the function of cohesin proteins in promoting replication fork progression has not been appropriately considered. It would be helpful for lay readers that a background on cohesin function in replication is provided and that the findings presented in this work are discussed in the light of previous insight.

As requested, we have added a paragraph of text to the Introduction section of the revised manuscript highlighting the previous literature regarding the role of the cohesin complex at the replication fork.

4. While the schematic model has been improved, pre-RC factors (CDC6 and CDT1, Geminin) are placed at replication forks, where they should not be present.

As suggested, we have modified the model to more accurately demonstrate that pre-RC factors CDC6, CDT1, and Geminin remain present at the origin recognition complex following STAG2 depletion.

We sincerely thank Reviewer #4 for his/her careful consideration of our manuscript and thoughtful suggestions for improvement. We greatly appreciate his/her dedication in helping to substantially improve the clarity and accuracy with which we have presented and discussed our results.

REVIEWERS' COMMENTS:

Reviewer #4 (Remarks to the Author):

The authors have introduced changes in the manuscript to address the comments from this reviewer.

- Text has been added to the discussion, which does not provide an explanation for the changes in the interaction of cohesin with replication proteins upon STAG2 interference, but acknowledges the complexity of their interpretation. Some statements are still somewhat confusing/hard to interpret (e.g. "We speculate that biding of cohesin with Geminin represents a critical mediator of an intra-S-phase checkpoint to ensure the establishment of sister chromatid cohesion during DNA replication". Authors are not measuring cohesion here. On what evidence is this speculation based? How would interaction between geminin and cohesin contribute to sister chromatid cohesion?).

- Authors have included a couple of sentences on how DNA replication is important to establish sister chromatid cohesion during S-phase in the introduction, but failed to provide a background on the role of cohesin in promoting replication fork progression/stability (set mostly by studies mentioned by this referee in the first round of revision).

- The accuracy of the model regarding the replication mechanism has been improved.

- Authors might want to use "replication fork progression" instead of "procession" in the abstract.

In my opinion, the manuscript is suitable for publication provided previous studies demonstrating a role for cohesin in promoting stable fork progression are discussed to set the background of the field for lay readers.

Reviewer #4

1. Text has been added to the discussion, which does not provide an explanation for the changes in the interaction of cohesin with replication proteins upon STAG2 interference, but acknowledges the complexity of their interpretation. Some statements are still somewhat confusing/hard to interpret (e.g. "We speculate that biding of cohesin with Geminin represents a critical mediator of an intra-S-phase checkpoint to ensure the establishment of sister chromatid cohesion during DNA replication". Authors are not measuring cohesion here. On what evidence is this speculation based? How would interaction between geminin and cohesin contribute to sister chromatid cohesion?).

The relevant sentences of the Discussion have been modified in the revised manuscript as requested.

2. Authors have included a couple of sentences on how DNA replication is important to establish sister chromatid cohesion during S-phase in the introduction, but failed to provide a background on the role of cohesin in promoting replication fork progression/stability (set mostly by studies mentioned by this referee in the first round of revision).

An additional sentence of background and two additional references have been added to the Introduction section of the revised manuscript as requested.
